# Procalcitonin testing combined with NEWS2 evaluation compared with usual care based on NEWS2 for identification of sepsis and antibiotic initiation in the emergency department in England and Wales (PRONTO): a multicentre, randomised, controlled, open-label, phase 3 trial

**DOI:** 10.1016/S2213-2600(25)00433-3

**Published:** 2026-05

**Authors:** Stacy Todd, Joanne Euden, Jennifer Condie, Stephen Aston, Gavin Barlow, Lucy Brookes-Howell, Julie Carman, Enitan D Carrol, Stephanie Gilbert, Philip Howard, Kerenza Hood, Matthew Inada-Kim, Martin J Llewelyn, Wakunyambo Maboshe, Fiona McGill, Sarah Milosevic, Emmanuel Nsutebu, Paul Schmidt, Andrew Tabner, David Taylor-Robinson, Abin Thomas, Emma Thomas-Jones, Ingeborg D Welters, Philip Pallmann, Neil French, PRONTO Trial Group, Neil French, Enitan D Carrol, David Taylor-Robinson, Stephen Aston, Ingeborg Welters, Emma Thomas-Jones, Joanne Euden, Wakunyambo Maboshe, Stephanie Gilbert, Philip Pallmann, Abin Thomas, Jennifer Condie, Lucy Brookes-Howell, Jackie Hughes, Sarah Milosevic, Sam Clarkstone, Mahuampi Perez-Alijas, Lena Meister, Alexandra Rollinson-Salter, Kerenza Hood, Luke Vale, Gabriella Culina-Jones, Emmanuel Nsutebu, David Lugo Palacios, Philip Howard, Matthew Inada-Kim, Julie Carman, Stacy Todd, Stephen Aston, Mary Brodsky, Dorothy Culpa, Rebecca Denton, Allayna Doherty, Amy Doyle, Claire Duffy, Jennifer Entwistle, Rachael Fergusson, Louise Fowler, Aaron Geoghegan, Sharon Glynn, Jessica Goncalves, Alvyda Gureviciute, Bindu Harikumar, Megan Howard, Emily Llangrath, Clare Jones, Oliver Jones, Jack Maher, Kate Maitland, Danielle Mclaughlan, Terrence McLoughlin, Nathalie Nicholas, Wafae Ouarch, Ian Quayle, Emma Richardson, Violet Ruhumbika, Deborah Scanlon, Nathan Sullivan, Eleanor Taylor-Barr, Shay Willoughby, Gavin Barlow, Fahed Bangash, Nathan Blott, Llucia Cabral-Ortega, Katie Drury, Angela Good, Donna Gotts, Rachel Harris, Matthew Hines, Claire Jones, Diana Kluczna, Patrick Lillie, Rosa McGing, Victoria Martinson, Isabel Mortimer, Mohammed Muddassir, Tanaraj Perinpanathan, Alexander Richards, Charlotte Smith, Debra Smith, Neil Smith, William Smith, Elizabeth Stones, Joseph Suich, Thomas Taynton, Gemma Walker, Karen Winter, Fiona McGill, Angelique Aspinwall, Showna Benjamin, Rebekah Burnham, Emma Carter, Kiran Chana, Sam Charlton, Suzie Colquhoun, Abu Hassan, Taj Hassan, Honorine Jobain, Gary Lamont, Tadas Mazeika, Michael Padden, Isabelle Rogers, Gaushiya Saiyad, Razan Saman, Wioletta Sobacka, Oliver Wordsworth, Robert White, Paul Schmidt, Sean Beech, Gyles Brown, Preeya Chauhan, Helen Claridge, Zoe Daly, Gemma Dixon, Sally Gosling, Andrew Gribben, Karen Hudson, Claudia Lameirinhas, Angela Nown, Steve Rose, Kerrie Scott, Susan Taylor, Chinazom Ugwueze, Martin Llewelyn, Chetan Trivedy, Raquel Akieme, Dina Alimari, Sara Appasamy, Geraldine Bassett, Carla Clegg, Raghavendra Devisetty, Mohamed Elouby, Kay Franklin, Jane Gaylard, Monica Gil, Andrew McGregor, Justyna Nowak, Laura Ortiz-Ruit de Godoa, Caroline Paley, Maya Perry, Denise Skinner, Nicola Skipper, Keely Stewart, Liam Todd, Penny Traviss, Elohor Uwadiogbu, Alan Wallace, Jonathan Underwood, Jade Cole, Nicholas Manville, Non Smith, Lauren Thomas, Rhys Thomas, Rajeev Madan, Raiiq Ridwan, Caitlin Adeniyi-Jones, Audrey Campbell, Emma Clark, Katie Coupe, Beverley Dickinson, Georgina Gosney, Susie Hardwick, Teresa Lareza, Pragya Mallick, Kerry Meynell, Alessandra Tidona, Rajeev Sharma, Maria Bokhari, James Harvey, Lillian Lee, Mehr Mehmood, Beverley McClelland, Jenny Ritzema, Helen Wild, Ann Wilson, Emma Tilley, Gemma Baldwin, Jennifer Bates, Madeleine Benson, Michael Connelly, Hary Coulton, Rebekah Da Silva Teixeira, Isabel Evans, Jennifer Griffiths, Jessica Hassell, Paula Hilltout, Anne-Marie Joyce, Jennie Lowdell, Amanda Tyler, Nick Vallotton, Deborah Ward, Carys Whitby, John-Paul Williamson, Jack Haslam, Louise Howard-Sandy, Grainne O'Connor, Georgia Moth, Sheila Munt, Jennifer Philbin, Sarah Warran, Sarah Winnard, Tanya Baron, Muhammad Faisal, Sally Beer, Joyce Chan, Phoebe Cherrington-Walker, Benjamin Clare-Gray, George Corby, Celia Diaz-Uribe, Karen Dineen, Abdala Espinosa, Alexis Espinosa, Dominique Georgiou, Katerina Gramm, Elizabeth Hatton, Ralph Hovet, Aimee Jeffs, Jacinta Kynaston, Hanxiao Li, Martina Iorio, Rosie Lynch, Rufino Magallano, Jose Martinez, Anna Mikanik, Alison Monk, Jane Okumoku, Tine Panduro, Tinelly Sambo, Nayanika Sreejith, Hannah Thraves, Jack Wilson, Joao Ziroldo, Mark Jadav, Benita Adams, Jane Agard, Monica Ayestaran, Charlotte Barker-Kirby, Sharon Botfield, Charlotte Bowyer, Jenna Datson, Eve Fletcher, Chrissie Hall, Fiona Hammonds, Claire James, Lily Jenkins, Sandra Kessly, Lindsay Knight, Catherine Lee-Kim-Koon, Cathal Murphy, Tara Murray, Kate Ralph, Peter Thomas, Sally Thomas, Leanne Trehouan, Richard Procter, Jonathan Bennett, Kerry Colling, Abigail List, Joanne Morley, Tracy Ruddick, Dean Wilkinson, Andrew Tabner, Graham Johnson, Suzanna Ballard, Elisha Cousins, Alison Fletcher, Charlotte Griffiths, Paolyn Guiling, Gareth Hughes, Ainsley MacShannon, Alison Matthews, Lucy-May Moulden, Alba Roberts, Alison Rockey, Hannah Scrafton, Thomas Ward, Lianne Wright, Heather Wroblewski, Marcelina Zawadzka, Roberta Branisteanu, Andrea Annoni, Imran Azeez, Bibi Badal, Martin Bailey, Louise Barnard, Joyce Benny, Ayeda Emran, Joanne Finn, Anna Geevasghese, Steven Hart, Karen Ixer, Rezma Miah, Victoria Mackenzie, Hannah Mugford, Patricia Nabayego, Julie Payne, Isma Rafiq, Camilla Ramus, Preyeta Saha, Nazreen Sahbeer, Ezaldeen Shareah, Abigail Smith, Jenny Styles, Jane Smith, Debbie Thomas, Shreevatsa Udupa, Abdalla Wheiba, David Martin, Chloe Bascombe, Nina Barratt, Matthew Bayliss, Faith Beecham, Karen Chapman, Hayley Chapple, Gary Cumberbatch, Yasmin De'ath, Rebecca Fletcher, Annette Fraine, Kathleen Horan, Charlotte Humphrey, Elizabeth Hurdidge, Emma Langridge, Cheryl Lindsay, Helen McHale, Rebecca Miln, Mirela Mukaj, Claire Osey, Karen O'Toole, Jodie Pinnock, Sally Pitts, Javen Ramsami, Suzanne Roffe, Joanna Samways, Sarah Savage, Natasha Tamoidi, Heather Tiller, Rebecca Troke, Luke Vamplew, James White, Annamaria Wilce, Ayaz Abbasi, Victor Ameh, Joshua Cooper, Sarah Liderth, Emma Robinson, Natalia Waddington

**Affiliations:** a Tropical and Infectious Disease Unit, Liverpool University Hospitals NHS Foundation Trust, Liverpool, UK; b Centre for Trials Research, College of Biomedical and Life Sciences, Cardiff University, Cardiff, UK; c Institute of Systems, Molecular and Integrative Biology, University of Liverpool, Liverpool, UK; d Hull York Medical School, University of York, UK; e Hull University Teaching Hospitals NHS Trust, Hull, UK; f UK Sepsis Trust, Walsall, UK; g Institute of Infection, Veterinary and Ecological Sciences, University of Liverpool, Liverpool, UK; h NHS England and NHS Improvement North-East and Yorkshire, Leeds, UK; i College of Biomedical and Life Sciences, Cardiff University, Cardiff, UK; j Hampshire Hospitals NHS Foundation Trust, Basingstoke, UK; k University of Southampton, Southampton, UK; l University Hospitals Sussex NHS Foundation Trust, Brighton, UK; m Brighton and Sussex Medical School, University of Sussex, Brighton, UK; n Leeds Teaching Hospitals NHS Trust, Leeds, UK; o Sheikh Shakhbout Medical City, Abu Dhabi, United Arab Emirates; p Portsmouth Hospitals University NHS Trust, Portsmouth, UK; q University Hospitals of Derby and Burton NHS Foundation Trust, Derby, UK; r School of Medicine, University of Nottingham, Queen's Medical Centre, Nottingham, UK; s Department of Public Health, Policy and Systems, University of Liverpool, Liverpool, UK; t Institute for Life Course and Medical Sciences, University of Liverpool, Liverpool, UK

## Abstract

**Background:**

Sepsis is a common and serious condition, defined as a dysregulated host response to infection, that leads to life-threatening organ dysfunction. In emergency department settings, accurate diagnosis can be challenging, as many non-infectious conditions have similar presenting features and there is no gold standard diagnostic test, which can lead to misdirected use of antibiotics. Procalcitonin is a well characterised biomarker that responds rapidly and with high specificity to the presence of bacterial infection. We aimed to investigate whether supplementing current practice with rapid procalcitonin testing would improve recognition of sepsis and allow reduced antibiotic prescribing with at least no change in overall mortality.

**Methods:**

A parallel, two-arm, open-label, individually randomised controlled trial was done in 20 hospital emergency departments within 17 National Health Service (NHS) Trusts or Health Boards across England and Wales. Patients aged 16 years and older with suspected sepsis were randomly assigned to either usual care or procalcitonin-guided care in a 1:1 ratio via a centrally controlled web-based randomisation programme. Participants, research staff, those assessing outcomes, and statisticians analysing the data were not masked to group assignment. Participants in the usual care group were assessed according to standard clinical management based on National Early Warning Score 2 (NEWS2). In the procalcitonin-guided care group, rapid procalcitonin testing was used in combination with NEWS2 assessment by use of a guidance-only algorithm for clinicians. This algorithm for clinical management was used for both usual care and procalcitonin-guided care groups and clinicians were free to use, ignore, or deviate from the algorithm. The co-primary endpoints were intravenous antibiotic initiation at 3 h (superiority) and 28-day mortality (non-inferiority) from triage assessment, assessed in all randomly assigned consenting participants with data for both co-primary outcomes available. Non-inferiority was concluded for 28-day mortality if the upper bound of the 90% CI was below a 2·5% margin on the risk difference scale. All patients who were randomly assigned to one of the two groups and who consented to data collection at baseline were included in adverse event analysis for the period they were included in the study. The study was registered with ISRCTN (ISRCTN54006056) and is complete.

**Findings:**

Between Nov 20, 2020, and Nov 1, 2023, a total of 7667 patients were recruited and randomly assigned to the usual care group (n=3836) or the procalcitonin-guided care group (n=3831). 5453 patients (2748 female, 2703 male, 1 non-binary, 1 data for gender missing) were included in the primary analysis population (2715 usual care, 2738 procalcitonin-guided care). The last 28-day follow-up was on Nov 29, 2023. There was no difference in intravenous antibiotic initiation at 3 h from triage assessment between the two groups: 48·4% (1325/2738 participants) in the procalcitonin-guided care group versus 48·2% (1308/2715 participants) in the usual care group (adjusted risk difference –0·08 percentage points, 95% CI –2·58 to 2·42; p=0·95). Mortality at 28 days was lower in the procalcitonin-guided care group: 13·6% (372/2738 participants) in the procalcitonin-guided care group versus 16·6% (450/2715 participants) in the usual care group (adjusted risk difference –3·12 percentage points, 90% CI –4·68 to –1·57; p=0·0009). The upper bound of the 90% CI was below the non-inferiority margin of 2·5 percentage points and the point of no effect, implying both non-inferiority and superiority at the one-sided 5% level. In the primary analysis population, the procalcitonin test result was considered in clinical decision making in 64·7% (1771/2738) of participants in the procalcitonin-guided care group. Improved mortality was not explained by findings of planned subgroup, sensitivity, or secondary analyses. A total of 180 adverse events were recorded, 53·3% (96 events in 57 [1·9%] of 2968 participants) in the usual care group and 46·7% (84 events in 66 [2·2%] of 3042 participants) in the procalcitonin-guided care group. One (<1%) of 2042 participants in the procalcitonin-guided care group reported a serious adverse event probably or definitely attributable to the procalcitonin test.

**Interpretation:**

Making a procalcitonin-guided algorithm available to clinicians in emergency departments did not change intravenous antibiotic initiation at 3 h in patients managed as suspected sepsis, but a decrease in 28-day mortality was seen and further research is needed to understand this finding.

**Funding:**

National Institute for Health and Care Research.

## Introduction

Sepsis is defined as life-threatening organ dysfunction caused by a dysregulated host response to infection^1^ and is a medical emergency requiring prompt antimicrobial therapy and physiological support. Delivering high-quality sepsis care in an emergency department setting remains challenging despite over 25 years of international guidance.^2^ Physiological-based scoring systems such as the Quick Sepsis Related Organ Failure Assessment (qSOFA) or National Early Warning Score 2 (NEWS2) have replaced systemic inflammatory response syndrome (SIRS) as screening tools, but these are non-specific and no gold standard diagnostic test exists. In an emergency department context, there is often diagnostic uncertainty, but delays in treatment have been shown to lead to poorer outcomes.^3^ The non-specific clinical presentation of sepsis is exacerbated by heterogeneous infection presentations (including non-bacterial causes) and many non-infection sepsis mimics, leading to recommendations that where sepsis is suspected prompt treatment should begin.^2^, ^4^ In line with international recommendations, UK National Institute for Health and Care Excellence (NICE) sepsis guidelines mandate the administration of intravenous antibiotics within 1 h to patients assessed to be at high risk for deterioration, admission to an intensive care unit (ICU) or high-dependency unit (HDU), or death.^4^ However, up to 30% of patients initially managed as sepsis in the emergency department do not have a discharge diagnosis of infection.^5^, ^6^, ^7^ The current approach to diagnosing sepsis leads to overuse of antibiotics with the associated risks of antimicrobial resistance, antibiotic-related drug reactions, *Clostridium difficile* infection, as well as extended lengths of hospital stay.^8^, ^9^

**Research in context**

**Evidence before this study**

In 2015, the UK National Institute for Health and Care Excellence (NICE; Westwood and colleagues) did a systematic review and cost-effectiveness analysis to evaluate the role of procalcitonin to guide antibiotic treatment of sepsis in intensive care settings and for suspected bacterial infection in emergency department settings. Although the review concluded that the addition of a procalcitonin algorithm could be a viable strategy to reduce antibiotic exposure and potentially reduce length of hospital stay, it showed heterogeneity in trial design and study populations. In particular, studies in emergency departments were heavily biased to those with a respiratory presentation rather than broader suspected bacterial infection and sepsis. We searched PubMed using the terms “sepsis” AND “procalcitonin” for systematic reviews or meta-analyses published between Jan 1, 2016, and Nov 30, 2024. The search was limited to publications in English. No formal appraisal of quality was undertaken. Updated reviews and patient-level meta-analyses from Wirz and colleagues (2018), Meier and colleagues (2019), Evans and colleagues (2021), and Papp and colleagues (2023 have reported mixed results—including potential for reduction in antibiotic prescribing and reduction of mortality in the intensive care setting and in subsets of sepsis patients with confirmed bacteraemia—but have not definitively shown the benefits of early biomarker-guided care. International sepsis guidance (including UK guidance) continues to recommend against adoption of procalcitonin in combination with clinical assessment for antibiotic initiation in suspected sepsis due to low quality of evidence and unclear costs to health systems. Addressing the role of biomarkers in the early identification and management of sepsis remains a high research priority.

**Added value of this study**

To the best of our knowledge, the PRONTO trial is the largest randomised controlled trial to evaluate the use of a procalcitonin-guided algorithm in the management of adults (16 years and older) presenting to emergency departments with suspected sepsis. The trial was designed to be as inclusive as possible, with a deferred consent model to ensure maximum participation across a range of disease severities and to minimise the impact of research-specific procedures. Procalcitonin testing was incorporated in an algorithm with clinical assessment that clinicians were free to use or ignore. The results confirmed that the procalcitonin-guided algorithm did not reduce intravenous antibiotic initiation at 3 h, but resulted in an a 3·12% absolute risk reduction in 28-day mortality, which persisted to 90 days.

**Implications of all the available evidence**

Adjustments to clinical management driven by the procalcitonin-guided algorithm do not fundamentally alter antibiotic initiation frequencies, but our findings indicate that they can improve short-term mortality rates. The evidence supports the value of early and rapid diagnostics and indicates a need for further biomarker and algorithm development. Uptake of procalcitonin-guided care into health systems will depend on greater understanding of the mechanism of effect, further health economic evaluations, and robust implementation frameworks.

Procalcitonin is a well characterised biomarker that responds rapidly and with high specificity to the presence of bacterial infection. At the point of the current trial design, procalcitonin testing systems were licensed for use in Europe and North America, had the most evidence available on diagnostic accuracy for identification of bacterial infections, and were commercially available through point-of-care devices. Procalcitonin-guided antibiotic initiation or discontinuation had been assessed in several trials across critical care and emergency departments. A NICE-funded systematic review and cost-effectiveness analysis published in 2015 evaluated the role of procalcitonin to guide antibiotic treatment of sepsis in intensive care settings and for suspected bacterial infection in emergency department settings.^10^ Although the study concluded that the addition of a procalcitonin algorithm could be a viable strategy to reduce antibiotic exposure and potentially reduce length of hospital stay, it showed the heterogeneity in trial design and study populations. In particular, studies in emergency departments were heavily biased to those patients with a respiratory presentation rather than broader suspected bacterial infection and sepsis, with a separate Cochrane meta-analysis^11^ showing that procalcitonin guidance could safely reduce antibiotic exposure and associated side-effects. However, there was inconsistent evidence for the benefit of procalcitonin use in the management of suspected sepsis in the emergency department setting^10^ and no evidence from the UK. Updated reviews and patient-level meta-analyses from 2018,^12^ 2019,^13^ 2021,^2^ and 2023^14^ have reported mixed results for the value of procalcitonin use, including potential for reduction in antibiotic prescribing and mortality in the intensive care setting and in subsets of sepsis patients with confirmed bacteraemia, but have not definitively shown the benefits of early biomarker-guided care. International sepsis guidance (including UK guidance) continues to recommend against adoption of procalcitonin in combination with clinical assessment for antibiotic initiation in suspected sepsis due to low quality of evidence and unclear costs to health systems.^2^, ^4^ The PRONTO trial was designed to address research recommendations from three separate NICE guidelines (NG51 [2016],^4^ DG18 [2015],^15^ and NG15 [2015]^16^) and internationally recognised research priorities^17^ to establish whether NEWS2 in combination with procalcitonin can improve the recognition of sepsis and facilitate prompt and appropriate antibiotic therapy in adults presenting to the emergency department.

The primary aim of PRONTO was to establish whether a risk stratification algorithm for clinicians based on point-of-care procalcitonin measurement in combination with NEWS2 leads to a reduction in intravenous antibiotic initiation at 3 h from the point of triage assessment, with no increase in 28-day mortality, compared with risk stratification by NEWS2 alone in patients with suspected sepsis presenting to hospital emergency departments in England and Wales.

## Methods

### Study design and participants

PRONTO was a parallel, two-arm, open-label, individually randomised controlled trial with two co-primary endpoints and group-sequential stopping rules for effectiveness. The trial protocol has been published previously and protocol deviations are reported in the appendix (p 15).^18^ Participants were recruited from 20 hospital emergency departments within 17 National Health Service (NHS) Trusts or Health Boards across England and Wales (appendix p 8). All sites had a locally adapted sepsis care pathway that was based on NICE suspected sepsis guideline NG51 (2016).^4^ In brief, this guideline requires clinically diagnosed suspicion of infection as a cause of illness with physiological compromise, but evidence of organ dysfunction or systemic inflammation is not essential. Sepsis pathways in UK emergency departments are typically initiated by triage nurses and usually before laboratory results are available. Although all sites used NEWS2 in accordance with NICE guideline NG51,^4^ sites were permitted to use additional tools (eg, UK Sepsis Trust Red Flag features, which include prompts such as clinical concern) in accordance with local policies to identify sepsis. No formal changes to local sepsis care pathways or the threshold at which local teams initiated sepsis pathways were required as part of the trial. All sites received standardised training and training slides were made available to local clinical and research teams to share information regarding use of the algorithm and trial procedures.

All patients aged 16 years or older who were managed by clinical teams as suspected sepsis using their local sepsis care pathway were potentially eligible for recruitment, irrespective of NEWS2. Exclusion criteria were: receiving intravenous antibiotics at the point of trial screening; current immunosuppressive chemotherapy; solid organ transplantation or allogeneic bone marrow or stem cell transplantation within 3 months before emergency department attendance; and known to require urgent surgical intervention. Patients receiving end-of-life care or those with an advance directive to withhold life-sustaining treatment that included antibiotic treatment were excluded, although those deemed to be unsuitable for cardiopulmonary resuscitation could be eligible if they were receiving other active treatments. Screening logs of eligible patients and randomisation were kept at each site and baseline participant characteristics including age, self-reported gender (options provided were female, male, or non-binary), and self-reported ethnicity were recorded.

Patients were identified at emergency department triage or initial assessment by clinical teams according to local pathways. The site trial team confirmed eligibility and, if no exclusion criteria applied, the patient was enrolled and randomly assigned. Given the urgent nature of the intervention, a deferred consent model was used (appendix p 10). The protocol was approved by the Wales REC 2 Research Ethics Committee (20/WA/0058) and the UK Health Research Authority and had oversight from an independent data monitoring committee (IDMC) and trial steering committee. The study was registered with ISRCTN (ISRCTN54006056) and is complete. Reporting follows the CONSORT guidance and relevant extensions.^19^, ^20^ There was active patient and public involvement at all stages of design, conduct, and reporting of the trial as detailed in the published protocol.^18^

### Randomisation and masking

**Figure 1:**
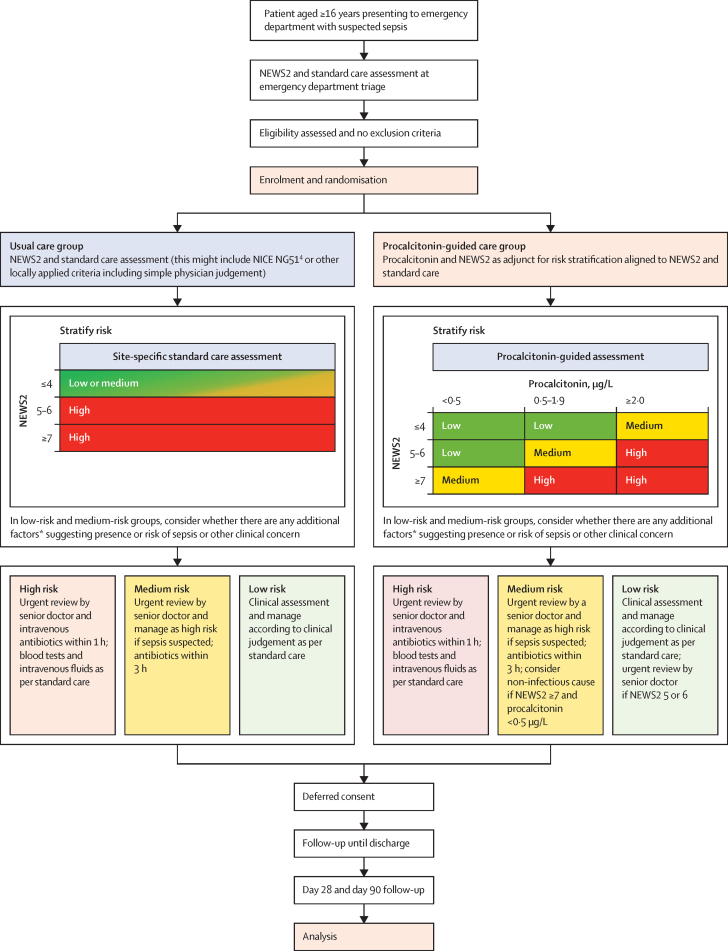
Trial schematic Modified from Euden at al.^18^ Recommendations for different risk groups based on NICE suspected sepsis guideline NG51.^4^ NEWS2=National Early Warning Score 2. NICE=National Institute for Health and Care Excellence. *Additional risk factors include: single NEWS2 parameter of 3; non-blanching rash or mottled, ashen, or cyanotic skin; reduced responsiveness; not passed urine in 18 h or reduced urine output; lactate greater than 2 mmol/L.

Participants were randomly assigned to standard clinical management with risk assessment based on NEWS2 scoring (usual care; control group) or clinical management based on NEWS2 and procalcitonin-guided risk assessment (procalcitonin-guided care; intervention group) in a 1:1 ratio (figure 1). Minimisation with NEWS2 (ie, ≤4, 5–6, or ≥7) and site as stratification factors was used with an added random element (80% chance of being allocated to the group minimising covariate imbalance) to reduce the risk of subversion.^21^ Allocation was determined via a secure web-based randomisation programme accessible 24 h by delegated research staff at all sites and controlled centrally by the Centre for Trials Research (CTR) at Cardiff University (Cardiff, UK). Participants, research staff, those assessing outcomes, and the statisticians analysing the data were not masked to group assignment.

### Procedures

In the procalcitonin-guided care group, procalcitonin was measured by use of one of two commercially available, CE-marked, point-of-care assays with results from both devices available in less than 20 min. The trial design was agnostic to device. BRAHMS PCT direct (ThermoFisher Diagnostics, Altrincham, UK) was used between Nov 20, 2020, and May 31, 2023, when the manufacturer removed it from the market. PathFast BRAHMS PCT (AB Scientific, London, UK) was used from Feb 24, 2023, to Nov 1, 2023. Eight sites used BRAHMS PCT direct from Nov 20, 2020, to May 31, 2023, and then changed to use of PathFast BRAHMS PCT between February, 2023, and May, 2023, and continued recruitment to November, 2023. 12 sites only used BRAHMS PCT direct and stopped recruiting on May 31, 2023. A blood sample was obtained from either venous blood draw (for both BRAHMS PCT direct and PathFast) or capillary sampling via finger prick (for BRAHMS PCT direct only). Although the BRAHMS PCT direct and PathFast BRAHMS tests have not been evaluated in a head-to-head study, both have been validated and approved for use on the basis of laboratory comparison studies.^22^, ^23^ The PathFast BRAHMS had a lower limit of quantitation, but both tests were validated for use at the procalcitonin thresholds incorporated in the trial algorithm (<0·5 μg/L, 0·5–1·9 μg/L, and ≥2·0 μg/L).^24^

In the procalcitonin-guided care group, the procalcitonin test was used in combination with NEWS2 assessment by use of a guidance-only algorithm for clinicians designed by the trial team and provided to sites as part of training. The algorithm for clinical management for both the usual care group and the procalcitonin-guided care group was based on NICE guideline NG51 (2016)^4^ and associated NHS England guidance,^25^ and categorised patients into low, medium, and high risk of progression to sepsis and death. The recommendations included whether antibiotics should be given within 1 h (for patients at high risk), up to 3 h (for patients at medium risk), or after clinical assessment (for patients at low risk; figure 1).^18^ Clinicians were permitted discretion to deviate from the algorithm at all times in both groups.

Details of patient disposition, antibiotic usage, adverse events, and microbiological investigations were captured via daily health record review from triage assessment until hospital discharge or day 28, whichever was sooner. Initial and discharge diagnoses were based on clinician medical notes. Mortality data to derive the co-primary outcome measure for safety (28-day mortality) and secondary outcome measure (90-day mortality) were sourced from health-care records. After a request from the IDMC, ethical and regulatory approval was obtained from Wales REC 2 and the UK Health Research Authority to allow the reporting of Trust-level numerical counts of mortality status by trial group of those who withdrew from the trial. Individual participant-level mortality status and all other data were collected only for those who actively consented into the trial. Safety information, including readmission, was recorded from health-care notes to day 90.

Planned day 28 and day 90 visits were done via telephone, or in person if a participant remained as an inpatient. Data collection at these visits focused on health-related quality of life and health-care resource utilisation questionnaires for use in health economic analysis. Qualitative interviews were done to aid understanding of the feasibility and acceptability of the trial intervention and research processes.

### Outcomes

The co-primary outcomes were intravenous antimicrobial initiation at 3 h from triage (assessing superior effectiveness) and 28-day mortality (assessing non-inferior safety). Secondary outcomes were: time until initiation of intravenous antibiotic therapy; late intravenous antibiotic initiation (ie, commenced after 3 h); number of days on intravenous antibiotics, broad spectrum antibiotics (defined as watch or reserve groups as per WHO AWaRe Classification),^26^ and any antibiotic during admission and total over the first 28 days; admission to ICU or HDU and length of stay in ICU or HDU; length of hospital stay; adverse antibiotic outcomes; readmission to hospital within 90 days; mortality within 90 days and time until death; health utility (EQ-5D-5L) at 28 and 90 days; health resource usage; feasibility of implementing procalcitonin testing alongside NEWS2 scoring in emergency departments; and acceptability of implementing procalcitonin testing alongside NEWS2 scoring to patients, carers, and clinicians. The health economic and qualitative outcomes will be reported separately.

Adverse events potentially attributable to the procalcitonin test were collected as part of routine follow-up at 28 days. Specific recording of antibiotic adverse events was included in adverse event recording and was also reported within secondary outcomes. Serious adverse event reporting was only required if events were probably or definitely attributable to the procalcitonin test, and resulted in persistent or significant disability or incapacity, or in a congenital anomaly or birth defect. Post-hoc analysis was done on data prospectively collected by sites but not included in the protocol planned primary or secondary analysis. This analysis included the timing and content of care: time between emergency department admission and triage assessment, triage assessment and randomisation, triage assessment and clinical risk assessment, clinical risk assessment and antibiotics prescription, antibiotics prescription and administration, and clinical risk assessment and review by senior doctor; type of antibiotic received within 12 h of triage assessment; ten most frequently initiated antibiotics within 12 h of triage assessment; 28-day mortality by type of antibiotic received within 12 h of triage assessment; oxygen therapy; grade of most senior clinician doing assessment; initial and discharge diagnoses; blood cultures taken within 24 h of triage assessment; 28-day mortality by discharge diagnosis; and 90-day mortality by discharge diagnosis.

### Statistical analysis

The statistical analysis plans for both the interim and the final analyses were completed before data lock and are available in the appendix (pp 35–75). The sample size was calculated based on the two co-primary outcomes. Calculations for the non-inferiority endpoint assumed a 28-day mortality of 15% in patients managed as suspected sepsis treated in the emergency department according to usual care.^5^, ^27^ Any increase to not more than 17·5% with procalcitonin-guided care was considered non-inferior. For 90% power and one-sided 5% significance level, the sample size required was 7002 participants, assuming there would be no difference in 28-day mortality between groups.^5^, ^28^ At the time of trial design, 90% of patients managed as suspected sepsis were receiving intravenous antibiotics within 3 h (Royal Liverpool and Broadgreen University Hospitals NHS Trust, unpublished data). As a non-infectious sepsis-mimic condition was expected in more than 20% of recruited patients,^5^, ^6^, ^7^ reducing antibiotic initiation to 80% or less was identified as a target for success. To detect such an effect with 90% power and a two-sided 5% significance level, the sample size required was 532 participants. Choosing the larger of these two sample sizes, including non-binding group-sequential stopping rules (one interim analysis after 50% of participants provided co-primary outcome data, by use of O'Brien–Fleming boundaries), and accounting for 5% dropout led to a total recruitment target of 7676 participants.

The primary analysis was performed based on a modified intention-to-treat population, including all consenting participants who were randomly assigned with available outcome data for both co-primary endpoints. Separate two-level logistic regression models (patients nested within sites) were fitted to both primary outcomes (antibiotic initiation and mortality), with fixed effects for randomised allocation and baseline NEWS2 (minimisation factor) and random site effects, to estimate adjusted odds ratios, which were then transformed into risk differences by use of standardisation. With the delta method as implemented in the Stata command adjrr,^29^ a 90% CI was derived for 28-day mortality, to conclude non-inferiority if its upper bound was below the margin of 2·5% on the risk difference scale, or superiority if the upper bound was below 0% on the risk difference scale. A 95% CI was derived for intravenous antibiotic initiation at 3 h, to conclude superiority if its upper bound was below 0% on the risk difference scale. Procalcitonin-guided care was only to be ruled better than usual care if it reduced antibiotic initiation in combination with non-inferior safety, or if superior safety was observed (appendix p 11). Prespecified subgroup analyses were performed by: organ system of infection (including non-infection); baseline NEWS2 category; management as suspected COVID-19; positive COVID-19 test result at time of admission (within 5 days of triage assessment); procalcitonin point-of-care device used; recruitment date; and amount of emergency department crowding. This subgroup analysis was done by fitting regression models with and without subgroup–arm interaction terms, and comparing them using a likelihood ratio test.

All secondary outcomes were also analysed in all randomly assigned consenting patients with available outcome data by use of two-level models appropriate to the type of outcome: Cox regression for time-to-event outcomes; negative binomial regression for over-dispersed count data (eg, days in hospital, days on antibiotics); and logistic regression for binary outcomes, with fixed effects for randomised allocation and baseline NEWS2, and random site effects. Time-to-event outcomes were additionally presented by use of Kaplan–Meier plots. All randomly assigned patients with consent up to day 28 were included in the analysis of adverse events. The frequency of adverse events and serious adverse events was tabulated overall and by trial arm and compared using a χ^2^ test.

Primary and secondary analyses were repeated with additional adjustment for age, gender, and the Charlson Comorbidity Index. These covariates were selected a priori based on associations with adverse outcomes in sepsis. Older age and higher comorbidity burden have been associated with worse outcomes in sepsis.^30^ The direction of gender effect varies across studies.^31^ A supplementary complier average causal effect (CACE) analysis estimated the difference in outcomes between participants whose treating clinicians complied with the intervention and those whose treating clinicians would have complied had they been allocated to the intervention (appendix p 10). In a sensitivity analysis, missing observations were replaced with multiple imputations via chained equations. Changes from initial to discharge diagnosis were presented with Sankey diagrams. The methods for post-hoc analyses were recorded within the statistical analysis plan (appendix pp 35–75) and were in line with primary and secondary analysis methods. All statistical analyses were done in Stata version 17 (interim analysis) or 18 (final analysis).

### Role of the funding source

The funder of the study had no role in study design, data collection, data analysis, data interpretation, or writing of the report.

## Results

**Figure 2:**
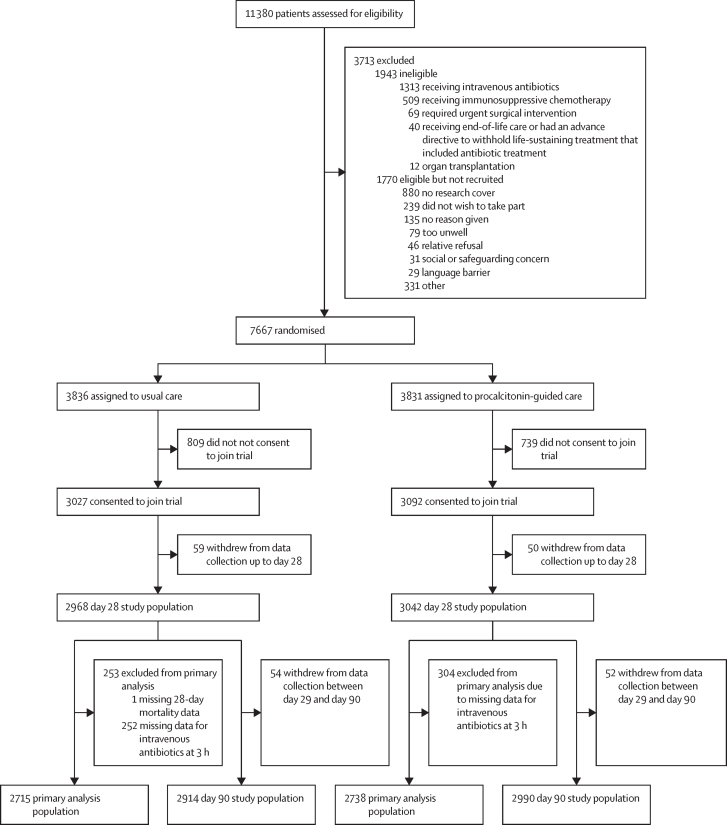
Trial profile

From a total of 11 380 patients screened for eligibility, 7667 were randomly assigned to the usual care group or the procalcitonin-guided care group between Nov 20, 2020, and Nov 1, 2023, with the last 28-day follow-up on Nov 29, 2023, and last 90-day follow-up on Jan 30, 2023. 1548 participants did not consent to join the trial or withdrew at a later point, leaving 6119 eligible for analysis at baseline (figure 2). 109 withdrew from data collection after baseline but within 28 days, leaving 6010 eligible for analysis up to day 28, of whom 5453 had data on both co-primary outcomes (primary analysis population). 106 withdrew from data collection between day 29 and day 90, leaving 5904 eligible for analysis up to day 90. The primary analysis of the co-primary endpoints was done on 5453 participants with complete data on both intravenous antibiotic initiation at 3 h and 28-day mortality: 2738 participants in the procalcitonin-guided care group and 2715 participants in the usual care group. Participants who did not have complete data for either endpoint were excluded from the primary analysis. The primary analysis population demographics and baseline characteristics were balanced across trial groups and are summarised in table 1, with full details of primary analysis population and baseline characteristics of all participants provided in the appendix (pp 12–15). In total, 30 protocol deviations were recorded, and these are summarised by categorisation in the appendix (p 15).

**Table 1:** Participant demographics and baseline characteristics of the primary analysis population

		**Procalcitonin-guided care (n=2738)**	**Usual care (n=2715)**
Age, years (median [IQR])		72 (56–82)	73 (58–82)
Gender			
	Male	1349 (49·3%)	1354 (49·9%)
	Female	1387 (50·7%)	1361 (50·1%)
	Non-binary	1 (<1%)	0
	Missing or not answered	1 (<1%)	0
Time between emergency department attendance and triage assessment, min (median [IQR])		19·8 (7·8–46·8)	16·8 (7·2–40·2)
Duration of symptoms before enrolment, h (median [IQR])		48 (24–96)	48 (18–96)
NEWS2			
	≤4	1036 (37·8%)	1051 (38·7%)
	5–6	737 (26·9%)	719 (26·5%)
	≥7	965 (35·2%)	945 (34·8%)
Ethnicity			
	White	2314 (84·5%)	2314 (85·2%)
	Asian	64 (2·3%)	45 (1·7%)
	Black	27 (1·0%)	23 (0·8%)
	Mixed	17 (0·6%)	17 (0·6%)
	Other	36 (1·3%)	28 (1·0%)
	Missing or not answered	280 (10·2%)	288 (10·6%)
History of oral antibiotics in the 14 days before admission			
	No	2046 (74·7%)	2021 (74·4%)
	Yes	628 (22·9%)	649 (23·9%)
	Missing or not answered	64 (2·3%)	45 (1·7%)
Charlson Comorbidity Index			
	Mean (SD)	3·9 (2·7)	4·0 (2·7)
	Median (IQR)	4 (2–6)	4 (2–6)
Diabetes		623 (22·8%)	651 (24·0%)
COPD		624 (22·8%)	615 (22·7%)
Moderate to severe CKD (stage 3 or above)		371 (13·6%)	372 (13·7%)
Heart failure		338 (12·3%)	378 (13·9%)
Ischaemic heart disease		287 (10·5%)	314 (11·6%)
Stroke or TIA		282 (10·3%)	292 (10·8%)
Dementia		253 (9·2%)	266 (9·8%)
Physical disability		249 (9·1%)	239 (8·8%)
Solid tumour malignancy		206 (7·5%)	204 (7·5%)
Myocardial infarction		180 (6·6%)	187 (6·9%)
CRP levels, mg/L (median [IQR])		68 (21–155)	61 (20–145)

Data are n (%) unless otherwise specified. CKD=chronic kidney disease. COPD=chronic obstructive pulmonary disease. CRP=C-reactive protein. NEWS2=National Early Warning Score 2. TIA=transient ischaemic attack.

**Figure 3:**
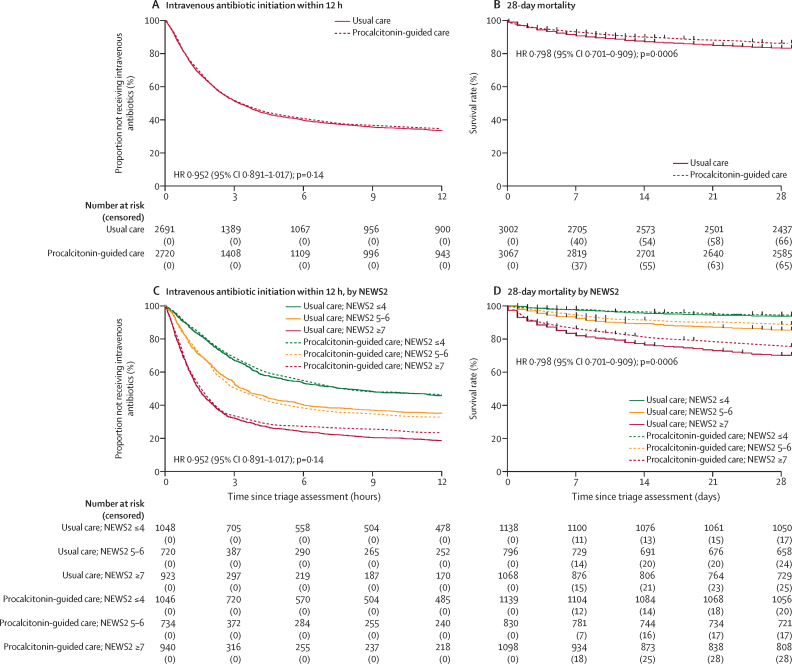
Kaplan–Meier survival estimates and Cox regression HRs of co-primary outcomes, adjusted for NEWS2 for all participants with relevant data and consent Populations for (A) and (C) include 5411 of 6119 participants who consented to join the trial; 708 participants were excluded due to missing start time of intravenous antibiotic initiation. (B) and (D) include 6069 participants: primary analysis population (n=5453) and enrolled participants with censored data (n=616 including withdrawals) during the first 28 days. HR=hazard ratio. NEWS2=National Early Warning Score 2.

There was no difference in proportion of participants receiving intravenous antibiotics within 3 h from triage assessment by group: 48·4% (1325/2738 participants) in the procalcitonin-guided care group versus 48·2% (1308/2715 participants) in the usual care group; estimated adjusted risk difference was –0·08 percentage points (95% CI –2·58 to 2·42; p=0·95). After additional adjustment for age, gender, and Charlson Comorbidity Index, the estimated adjusted risk difference was 0·15 percentage points (95% CI –2·37 to 2·66; p=0·91; appendix p 16). O'Brien–Fleming-adjusted CI bounds were similar to regular ones. Kaplan–Meier curves show no evidence of divergence of time to initiation of antibiotic by group over the first 12 h (figure 3).

Mortality at 28 days from triage assessment was lower in the procalcitonin-guided care group than in the usual care group: 13·6% (372/2738 participants) in the procalcitonin-guided care group versus 16·6% (450/2715 participants) in the usual care group; estimated adjusted risk difference was –3·12 percentage points (90% CI –4·68 to –1·57; p=0·0009). After additional adjustment for age, gender, and Charlson Comorbidity Index, the estimated adjusted risk difference was –2·35 percentage points (90% CI –3·85 to –0·86; p=0·0094). The upper bounds of the 90% confidence intervals from both analyses were below both the non-inferiority margin of 2·5 percentage points and the point of no effect, implying both non-inferiority and superiority at the one-sided 5% level (figure 3; appendix p 16).

None of the prespecified subgroup analyses yielded significant differences in the co-primary treatment effect on formal test for interaction (appendix p 16). The subgroup analysis of 28-day mortality based on whether participants had received a positive COVID-19 test result within 5 days of triage assessment could not be performed, as no deaths occurred in the usual care group, preventing convergence of the analysis model (appendix p 16). Mortality outcome in several clinically relevant subgroups was not statistically significant (appendix pp 17–18).

Co-primary outcome effects were similar across all planned sensitivity analyses. Use of the complete case populations for each of the co-primary outcomes separately resulted in similar effect estimates (appendix p 19). After adjustment, mortality within 28 days from triage assessment was lower in the procalcitonin-guided care group compared with the usual care group (–3·00 percentage points, 90% CI –4·50 to –1·50; p=0·0010), but there was no difference in intravenous antibiotic initiation within 3 h between the two groups (–0·22 percentage points, 95% CI –2·70 to 2·26; p=0·99). The co-primary analyses after imputing missing data by use of multiple imputation provided results that were also consistent with these findings (appendix p 19).

A planned interim analysis was undertaken after 43% of target recruitment was reached, followed by an IDMC-initiated second interim analysis after 57% target recruitment. Although both analyses triggered the stopping rule for superiority, the IDMC recommended continuing recruitment to the originally planned target (appendix p 20).

Crude analysis of deaths revealed lower mortality in withdrawn participants than in consented participants: 90-day mortality rate was 9·1% (141/1548 withdrawn participants), with 74 deaths in the procalcitonin-guided care group and 67 deaths in the usual care group (appendix p 20). There was no evidence of a systematic difference between groups in withdrawn participants.

For the 3092 participants assigned to the procalcitonin-guided care group who consented to join the trial, a procalcitonin test result was available for 94·8% (2930/3092) of participants (table 2). Of the 1944 classified as high risk by NEWS2 alone, addition of procalcitonin reclassified 479 (24·6%) as low risk and 641 (33·0%) as medium risk (appendix p 21). For the procalcitonin-guided care group, a procalcitonin test result was considered in clinical decision making (ie, a procalcitonin test result was available and seen by the clinician) in 63·5% (1962/3092) of participants, and for 46·2% (1430/3092) of participants the clinician reported compliance with the algorithm (ie, the clinician had seen the result and agreed with the recommendation arising from the algorithm risk score). In the primary analysis population, a procalcitonin test result was available and seen by the clinician in 64·7% (1771/2738) of participants and the clinician had seen the procalcitonin result and agreed with the recommendation arising from the algorithm risk score in 47·2% (1293/2738) of participants. CACE estimates based on these definitions of rates of adherence are consistent with the conclusion of superiority in terms of 28-day mortality and no evidence of effect in terms of intravenous antibiotic initiation within 3 h (table 3).

**Table 2:** Analysis populations defined by adherence to the procalcitonin-guided care algorithm

	**Procalcitonin-guided care (n=3092)**
**Procalcitonin test was done and a result was available**	
Yes	2930 (94·8%)
No	162 (5·2%)
Missing	0 (0·0%)
**Procalcitonin result was available and the clinician had seen the result**	
Yes	1962 (63·5%)
No	1056 (34·2%)
Missing	74 (2·4%)
**Procalcitonin result was available and the clinician had seen the result and agreed with the recommendation arising from the algorithm risk score**	
Yes	1430 (46·2%)
No	1558 (50·4%)
Missing	104 (3·4%)

Data are n (%).

**Table 3:** CACE analyses

		**28-day mortality (n=5940)**	**Intravenous antibiotic initiation at 3 h (n=5482)**
Complete case OR		0·818 (0·702–0·953)	0·999 (0·892–1·119)
Levels of adherence, CACE OR			
	Procalcitonin test was done and a result was available	0·790 (0·700–0·892)	0·999 (0·892–1·118)
	Procalcitonin result was available and the clinician had seen the result	0·779 (0·637–0·953)	1·017 (0·867–1·194)
	Procalcitonin result was available and the clinician had seen the result and agreed with the recommendation arising from the algorithm risk score	0·695 (0·522–0·924)	1·027 (0·819–1·287)

Data are OR (95% CI). Analysis method was two-level logistic regression models. Covariates were baseline NEWS2, age, gender, and Charlson Comorbidity Index. For this analysis, the population for 28-day mortality included 3000 participants from the procalcitonin-guided care group and 2940 participants from the usual care group, and the population for intravenous antibiotic initiation at 3 h included 2744 participants from the procalcitonin-guided care group and 2738 participants from the usual care group (appendix p 10). CACE=complier average causal effect. OR=odds ratio.

In the planned secondary outcome analysis, there was no difference in intravenous antibiotic initiation within 3 h across baseline NEWS2 (figure 3), although antibiotics were more frequently prescribed in higher NEWS2 groups. Mortality at 90 days was lower in the procalcitonin-guided care group, with 20·0% (598/2988) of participants dying within 90 days compared with 23·8% (693/2909) in the usual care group (estimated adjusted risk difference –3·08 percentage points, 90% CI –4·70 to –1·45; p=0·0018; table 4). Time to death was also significantly different between groups. A significant interaction was estimated between deprivation decile and procalcitonin-guided care for mortality outcome, with the largest benefits in those in the most deprived groups (appendix p 22). There was no statistically significant treatment effect on any other secondary outcome (table 4). Days on antibiotics (any, intravenous, and broad spectrum) were similar in both treatment groups (table 4; appendix pp 23–24). There were more ICU and HDU admissions in the procalcitonin-guided care group, but the difference between groups was not statistically significant. A total of 180 adverse events were recorded over 28 days, 53·3% (96 events in 57 [1·9%] of 2968 participants) in the usual care group and 46·7% (84 events in 66 [2·2%] of 3042 participants) in the procalcitonin-guided care group (χ^2^ [df 1]=1·47, p=0·23 for difference between groups; appendix p 25). Specific adverse events recorded in secondary analysis included antibiotic adverse outcomes and *C difficile* infection (table 4). One serious adverse event (persistent or significant disability or incapacity) was reported in the procalcitonin-guided care group (appendix p 25).

**Table 4:** Planned secondary outcome analyses

	**Procalcitonin-guided care**	**Usual care**	**Treatment effect estimate**	**p value**
Hours until initiation of intravenous antibiotic therapy*				
Primary	1·82 (0·78–4·03); n=1918	1·83 (0·78–4·05); n=1921	HR 0·953 (0·895–1·016)	0·14
Adjusted	1·81 (0·78–4·03); n=1892	1·83 (0·78–4·03); n=1898	HR 0·964 (0·905–1·028)	0·27
**Intravenous antibiotic initiation after 3 h**†				
Primary	625/2675 (23·4%)	640/2636 (24·3%)	OR 0·955 (0·839–1·086)	0·48
Adjusted	614/2635 (23·3%)	631/2606 (24·2%)	OR 0·966 (0·848–1·101)	0·61
**Days on intravenous antibiotics during admission**‡				
Primary	3 (0–6); n=2958	3 (1–6); n=2884	IRR 1·000 (0·937–1·068)	0·99
Adjusted	3 (0–6); n=2915	3 (1–6); n=2855	IRR 1·000 (0·936–1·067)	0·99
**Days on intravenous antibiotics during first 28 days**‡				
Primary	3 (0–6); n=2958	3 (1–6); n=2884	IRR 0·990 (0·930–1·054)	0·76
Adjusted	3 (0–6); n=2915	3 (1–6); n=2855	IRR 0·991 (0·931–1·055)	0·78
**Days on any antibiotic during admission**‡				
Primary	5 (1–9); n=2965	5 (1–9); n=2900	IRR 1·018 (0·960–1·079)	0·56
Adjusted	5 (1–9); n=2922	5 (1–9); n=2871	IRR 1·013 (0·956–1·074)	0·66
**Days on any antibiotic during first 28 days**‡				
Primary	5 (1–9); n=2965	5 (1–9); n=2900	IRR 1·000 (0·948–1·055)	1·00
Adjusted	5 (1–9); n=2922	5 (1–9); n=2871	IRR 1·005 (0·952–1·060)	0·86
**Days on broad-spectrum antibiotics (intravenous and oral) during admission**‡				
Primary	1 (0–6); n=2956	1 (0–5); n=2883	IRR 1·065 (0·975–1·162)	0·16
Adjusted	1 (0–6); n=2914	1 (0–5); n=2853	IRR 1·050 (0·961–1·146)	0·28
**Days on broad-spectrum antibiotics (intravenous and oral) during first 28 days**‡				
Primary	1 (0–6); n=2956	1 (0–5); n=2883	IRR 1·007 (0·927–1·095)	0·87
Adjusted	1 (0–6); n=2914	1 (0–5); n=2853	IRR 1·006 (0·925–1·094)	0·89
**ICU or HDU admission**†				
Primary	195/2966 (6·6%)	161/2876 (5·6%)	OR 1·182 (0·950–1·470)	0·13
Adjusted	192/2925 (6·6%)	161/2847 (5·7%)	OR 1·139 (0·913–1·420)	0·25
Days until ICU or HDU admission (excluding those who died)*				
Primary	1 (0–2); n=136	1 (0–1); n=105	HR 1·186 (0·919–1·530)	0·19
Adjusted	1 (0–2); n=134	1 (0–1); n=105	HR 1·169 (0·905–1·510)	0·23
Days until ICU or HDU admission (including those who died)*				
Primary	1 (0–1); n=194	1 (0–1); n=161	HR 1·164 (0·945–1·435)	0·15
Adjusted	1 (0–2); n=191	1 (0–1); n=161	HR 1·125 (0·912–1·388)	0·27
**Length of ICU or HDU stay (excluding those who died), days**‡				
Primary	7 (4–11); n=102	5 (3–9); n=88	IRR 1·045 (0·775–1·409)	0·77
Adjusted	7 (4–11); n=101	5 (3–9); n=88	IRR 1·029 (0·765–1·383)	0·85
**Length of ICU or HDU stay (including those who died), days**‡				
Primary	6 (3–13); n=160	5 (3–9); n=143	IRR 1·189 (0·944–1·496)	0·14
Adjusted	7 (3–13); n=158	5 (3–9); n=143	IRR 1·215 (0·966–1·528)	0·10
**Length of hospital stay (excluding those who died), days**‡				
Primary	6 (2–12); n=2319	6 (2–12); n=2180	IRR 0·973 (0·917–1·034)	0·38
Adjusted	6 (2–12); n=2283	6 (3–12); n=2162	IRR 0·976 (0·921–1·035)	0·42
**Length of hospital stay (including those who died), days**‡				
Primary	6 (3–13); n=2922	6 (3–13); n=2882	IRR 0·983 (0·933–1·036)	0·52
Adjusted	6 (3–13); n=2881	6 (3–13); n=2853	IRR 0·992 (0·942–1·044)	0·75
**Adverse antibiotic outcomes**†				
Primary	56/2798 (2·0%)	53/2764 (1·9%)	OR 1·050 (0·718–1·538)	0·80
Adjusted	56/2768 (2·0%)	53/2738 (1·9%)	OR 1·056 (0·721–1·547)	0·78
**Hospital-acquired infections**†				
Primary	163/2878 (5·7%)	152/2802 (5·4%)	OR 1·056 (0·839–1·328)	0·65
Adjusted	163/2846 (5·7%)	151/2775 (5·4%)	OR 1·079 (0·856–1·359)	0·52
**Clostridium difficile cases**†				
Primary	46/2578 (1·8%)	44/2526 (1·7%)	OR 1·044 (0·685–1·591)	0·84
Adjusted	46/2543 (1·8%)	44/2501 (1·8%)	OR 1·071 (0·702–1·635)	0·75
**Hospital readmission within 90 days**†				
Primary	124/2971 (4·2%)	136/2907 (4·7%)	OR 0·883 (0·687–1·135)	0·33
Adjusted	122/2928 (4·2%)	135/2877 (4·7%)	OR 0·884 (0·686–1·138)	0·34
**Mortality within 90 days**†				
Primary	598/2988 (20·0%)	693/2909 (23·8%)	OR 0·776 (0·682–0·883)	0·0001
Adjusted	593/2947 (20·1%)	682/2879 (23·7%)	OR 0·804 (0·701–0·922)	0·0018
Time until death, days*				
Primary	12 (4–34); n=598	12 (4–33); n=693	HR 0·803 (0·720–0·896)	0·0001
Adjusted	12 (4–35); n=593	12 (4–34); n=682	HR 0·835 (0·748–0·932)	0·0013

Data are median (IQR) or n/N (%) unless otherwise specified. Effect estimates (HR, OR, or IRR) are presented with 95% CI in parentheses. Analysis methods were mixed effects with Cox regression, logistic regression, negative binomial regression, or linear regression. Covariate in primary models was baseline NEWS2. Covariates in adjusted models were baseline NEWS2, age, gender, and Charlson Comorbidity Index. HDU=high-dependency unit. HR=hazard ratio. ICU=intensive care unit. IRR=incidence rate ratio. NEWS2=National Early Warning Score 2. OR=odds ratio.

* Cox regression.

† Logistic regression.

‡ Negative binomial regression.

To further explore the potential mechanism behind the co-primary outcome findings, several additional post-hoc analyses were done investigating the timing and content of care. Timings of participant flow through the emergency department from admission to triage assessment, randomisation, and antibiotic prescription and administration were similar between groups (appendix p 26). Primary route of antibiotics (either broad or narrow spectrum) in the first 12 h was intravenous in both groups but the distribution of route (or no antibiotic) varied across baseline NEWS2 category and trial group (appendix p 27). The ten most frequently initiated antibiotics within 12 h were similar between groups (appendix p 27). 28-day mortality by initial antibiotic route and baseline NEWS2 showed variation between trial groups (appendix p 28). Oxygen was required in around half of patients in both groups, with no significant difference detected between groups (appendix p 29). No significant difference in co-primary outcomes was detected by subgroup analysis for grade of clinician performing the initial assessment (appendix p 29).

Initial and discharge diagnoses were similar across both groups, with the most common discharge diagnosis being lower respiratory tract infection or community-acquired pneumonia (appendix pp 30–31). Change in diagnosis between initial and discharge diagnosis occurred in both groups, with the largest changes reported for initial diagnosis categories of sepsis (unknown source) and not infection. The number and timing of blood cultures taken were similar across groups (appendix p 31). Of those who initially consented to join the trial, a blood culture grew a bacterial species in 207 (6·7%) of 3092 participants in the procalcitonin-guided care group and 197 (6·5%) of 3027 participants in the usual care group. Primary causative bacteria were *Escherichia coli* (n=204), *Staphylococcus aureus* (n=79), *Klebsiella pneumoniae* (n=43), and *Streptococcus pneumoniae* (n=40). Mortality was lower among those in the procalcitonin-guided care group for both infection and non-infection discharge diagnoses (appendix pp 32–33).

## Discussion

In this large trial of patients presenting to emergency departments managed as suspected sepsis, the addition of point-of-care procalcitonin testing to current NEWS2-based risk assessment did not lead to a reduction in intravenous antibiotic use, but was associated with a significant reduction in 28-day mortality that extended to 90 days. Lower mortality was seen in patients with both infection and non-infection discharge diagnoses.

The absence of demonstrable effectiveness in reducing antibiotic use could, in part, be explained by a wider change in antibiotic prescribing driven by national antimicrobial stewardship efforts to improve targeting of therapy. Overall rates of intravenous antibiotics initiation in the first 12 h in the usual care group were 65·5% (1789/2691 participants), less than our pre-trial expected 90% usage at 3 h in the usual care group and ou r pre-trial specified superiority target rate of 80%. During the time from trial conception to completion, there has been a strong emphasis on antimicrobial stewardship within the NHS,^32^ and during the COVID-19 pandemic, there was improved recognition of viral sepsis without bacterial co-infection.^33^ In addition, the Surviving Sepsis Campaign Guidelines 2021 were updated to allow up to 3 h for initial assessment to optimise antimicrobial choice or to identify non-infectious mimics in patients with suspected sepsis without shock, whereas the previous recommendation for immediate antibiotics (within 1 h) was instead targeted to those with septic shock.^2^ This recommendation was adopted in the NICE guideline in 2024, after PRONTO trial recruitment was completed;^4^ however, these changes were already present in the Academy of Medical Royal Colleges guidance from 2022, and are therefore likely to have affected UK practice.^34^ Overall, the capacity of the trial intervention to show a difference in antibiotic prescribing might have been largely removed. We did not specify type and duration of antibiotic to be used in our protocol and expected antibiotics to be prescribed in accordance with hospital formularies. Similarly, clinicians were free to use further biomarkers during hospital stay to inform management based on local policy and clinician preference. Procalcitonin was not part of sepsis management guidelines at sites during the study, with the exception of one site (which recruited 158 [2·1%] of 7667 participants). This site incorporated procalcitonin measurement into their sepsis pathway in March, 2023, and finished recruiting in May, 2023.

The reduction in mortality among those who received procalcitonin-guided care was considered a possible trial outcome, as noted in earlier published reports.^10^, ^11^, ^12^ However, a clear mechanism to explain this mortality reduction was not identified. This was an open-label study, raising the possibility of both conscious and unconscious bias in clinical decision making through the Hawthorne effect.^35^ The low heterogeneity of the mortality effects across sites makes this an unlikely explanation (appendix p 33). We know that there was variation in the staff groups involved in implementation of the intervention across sites, which would be expected to increase heterogeneity by site, whereas the extent of improvement from usual care reached by a Hawthorne effect would be unlikely to be similar across a range of sites with different clinical pressures and performance levels. A by-chance finding is also unlikely. The CACE analysis shows a strengthening of effect with increasing adherence to the algorithm, supporting a true effect. Additionally, the modification of the observed effect by deprivation—in which patients in the most deprived areas have the greatest mortality benefit from procalcitonin-guided care—further supports a true and context-specific effect. This finding might reflect a plausible mechanism, whereby procalcitonin-supported care functions as a levelling-up intervention, helping to streamline care pathways and overcome barriers to access that are more prevalent among disadvantaged populations.^36^

Adherence to the recommendations of the procalcitonin-guided care algorithm was reported in 1430 (46·2%) of 3092 participants who consented to join the procalcitonin-guided care group, whereas 1130 (36·5%) of 3092 participants were managed without consideration of the procalcitonin result (ie, procalcitonin test result was unavailable or result was available but clinician did not see the result). The finding that the procalcitonin result was reviewed but the algorithm was not followed in 532 (17·2%) of 3092 participants suggests an absence of confidence or poor utility of the algorithm (ie, the algorithm did not address clinical uncertainty in a proportion of patients). The uptake of the algorithm was higher than for similar interventions,^37^, ^38^ but there are complex reasons why clinicians might or might not follow the algorithm. Analysis of interviews with patients and clinicians will be reported separately. The low adherence could be one reason why the algorithm did not identify a difference in antibiotic prescribing, although the CACE analysis would suggest little difference in prescribing practice whether the algorithm was fully applied or not. However, increased use of the algorithm could improve mortality outcomes further.

This trial has several strengths and limitations. The trial was large and pragmatic. Despite unprecedented pressures in the UK NHS due to the COVID-19 pandemic and emergency department overcrowding, the recruitment target was reached. The co-primary outcomes captured both effectiveness and safety, including mortality. The trial used a deferred consent model, which had both benefits and disadvantages. Patients being managed as suspected sepsis in emergency departments are a heterogeneous group in terms of severity of illness, medical needs, and communication requirements. Use of deferred consent allowed us to include this important variety. However, we did have a higher-than-expected rate of non-consent to join the trial after randomisation (1548 [20·2%] of 7667 participants), although the withdrawal rate is consistent with that of other studies that have used this approach.^39^ With ethics approval, we were able to count deaths by study arm in the withdrawn group and confirm that no major bias was created by this effect. Mortality was lower in the withdrawn group, but this result should be interpreted cautiously as we had no active follow-up of these withdrawn participants and mortality was based on hospital reports only; deaths within the community could have been missed at the time of data lock. The trial was undertaken in a variety of emergency departments across England and Wales, representing different demographic groups and excluding only those with identified severe immunosuppressive conditions. There was under-representation of non-White ethnicity, in part a consequence of the best-recruiting sites having lower ethnic diversity than the UK as a whole. Procalcitonin response has not been shown to differ by ethnicity,^40^ but this should be considered in further evaluations.

We hypothesise that the use of the procalcitonin-guided algorithm leads to multiple subtle changes in clinician behaviour that we were unable to fully measure in this trial. The mortality benefits were evident across conditions that were finally diagnosed as both infection and non-infection, indicating that some component of improved care was created by use of the procalcitonin-guided algorithm that was not restricted to improved infection management. Unfortunately, the process evaluation of this intervention has not provided a clear mechanism to explain the findings. Future work will need to focus on the characteristics and timeliness of further investigations and interventions relevant to the discharge diagnosis. Making a procalcitonin-guided algorithm available to clinicians in emergency departments did not change intravenous antibiotic initiation in patients managed as suspected sepsis, but a decrease in mortality was seen and further research is needed to understand this finding.

**This online publication has been corrected. The corrected version first appeared at thelancet.com/respiratory on March 25, 2026**

### Contributors

### Data sharing

The data are held by the Centre for Trials Research, Cardiff, UK. Applications to share the deidentified and anonymised trial data with other investigators for use in future research will be considered, subject to review of the aims and scientific methods of the application, and any contractual obligations required by organisations involved in the study. Requests for data sharing or collaboration should be made to the corresponding author.

## Declaration of interests

ST is Co-Chief Investigator for the National Institute for Health and Care Research (NIHR) Health Technology Assessment (HTA) grant PRONTO (17/136/13), was co-applicant for an Innovate UK grant (10026942), and was Co-Investigator and received NIHR HTA funding for the PEACH study (NIHR132254). JE was Co-Investigator and received NIHR HTA funding for the PEACH study (NIHR132254) and PROTECT study (NIHR156664). LB-H was Co-Investigator and received NIHR HTA funding for the PEACH study (NIHR132254) and the NIHR HTA BATCH trial (NIHR15/188/42). EDC was Co-Chief Investigator and received NIHR HTA funding for the PROTECT acceleration award (NIHR156664); was Chief Investigator and received NIHR HTA funding for the BATCH trial (NIHR15/188/42); was Co-Chief Investigator and received Medical Research Council (MRC)–NIHR Efficacy and Mechanism Evaluation (EME) funding for the PRECISE study (NIHR129960); has received scientific advisory board consulting fees from ThermoFisher Scientific, bioMerieux, and Danaher (paid directly to University of Liverpool); was a member of the NIHR Invention for Innovation panel (from November, 2011, to November, 2023); was a member of the National Institute for Health and Care Excellence (NICE) Diagnostic Advisory Committee (from April, 2014, to September, 2020); was a member of the MRC Developmental Pathway Funding Scheme panel (from March, 2020 to March, 2023); was a member of MRC COVID-19 AGILE panel (from July, 2020, to September, 2021); is a member of the NIHR HTA Programme Funding Committee (January, 2025, to present); and is an NIHR Senior Investigator. KH was a member of the NIHR HTA General Committee from 2016 to 2022. MI-K has received payments for speaking at Healthcare Conferences UK Deteriorating Patient Summits (all payments donated to charities); is NHS England National Clinical Director for Infection and Antimicrobial Resistance (payment for seconded time); is a UK Health Security Agency honorary fellow (unpaid); and is Royal College of Physicians Clinical Lead for Deterioration and NEWS (unpaid). MJL has received NIHR grants for the following projects: ARK Digitally Enabled Sustainable Implementation (NIHR206517); Staphylococcus Aureus Network Adaptive Platform (SNAP Trial; NIHR133719); and Duration of Antibiotic Treatment in Urinary Tract Infection (DurATIon-UTI Trial; NIHR134854). MJL is also an Independent Data Monitoring Committee member of the NIHR-funded SHORTER trial (NIHR134101). PS was Chief Investigator and received Presymptom Health funding for the PRECISION study and has received consulting fees from Presymptom Health. DT-R is funded by the NIHR on a Research Professorship (NIHR302438); has received grant funding from NIHR (NIHR204000), Wellcome Trust (217067/Z/19/Z and 226630/Z/22/Z), Health Foundation (FR-0002996), and UK Cystic Fibrosis Trust (SRC 027); and was paid a consultancy fee for preparing a report and presenting evidence at the UK COVID inquiry. ET-J was a Co-Investigator and received NIHR HTA funding for the PEACH study (NIHR132254), PROTECT study (NIHR156664), BATCH trial (NIHR15/188/42), and MRC–NIHR EME funding for the PRECISE study (NIHR129960). PP was a Co-Investigator and received NIHR HTA funding for the BATCH trial (NIHR15/188/42) and PEACH study (NIHR132254), was Co-Chief Investigator for the PROTECT study (NIHR156664), and received MRC–NIHR EME funding for the PRECISE study (NIHR129960). NF, as Chief Investigator for the PRONTO trial, was provided with BRAHMS PCT direct readers from ThermoFisher Scientific and PathFast BRAHMS PCT machines from AB Scientific for the trial. He is an investigator on grants from MRC (APP43907), Innovate UK (971554), Wellcome Trust (223502/Z/21/Z and 316932/Z/24/Z), the Gates Foundation (OPP1208803), and an NIHR Health Protection Research Unit (NIHR200907). He has received grants from Seqirus and GSK on topics unrelated to diagnostics, and has received consulting fees from Seqirus for advice on respiratory viral vaccines, paid to the University of Liverpool. The Centre for Trials Research (JE, JCo, LB-H, SG, WM, SM, ATh, ET-J, and PP) receives infrastructure funding from Health and Care Research Wales. All other authors declare no competing interests.
