# Supplementary material for: Procalcitonin testing combined with NEWS2 evaluation compared with usual care based on NEWS2 for identification of sepsis and antibiotic initiation in the emergency department in England and Wales (PRONTO): a multicentre, randomised, controlled, open-label, phase 3 trial
Source: Lancet Respir Med. 2026 May;14(5):417–31. doi: 10.1016/S2213-2600(25)00433-3 (PMC13125698; doi:10.1016/S2213-2600(25)00433-3)
Supplement: Supplementary appendix [file mmc1.pdf]

THE LANCET
Respiratory Medicine

Supplementary appendix

This appendix formed part of the original submission and has been peer reviewed. We post it as supplied by the authors.

Supplement to: Todd S, Euden J, Condie J, et al. Procalcitonin testing combined with NEWS2 evaluation compared with usual care based on NEWS2 for identification of sepsis and antibiotic initiation in the emergency department in England and Wales (PRONTO): a multicentre, randomised, controlled, open-label, phase 3 trial. *Lancet Respir Med* 2026; published online March 22. https://doi.org/10.1016/S2213-2600(25)00433-3

Procalcitonin and NEWS2 evaluation for Timely identification of sepsis and Optimal use of antibiotics in the Emergency Department (PRONTO): A multi-centre, randomised, controlled, open-label, phase 3 trial

Supplementary Material
10 February 2026

Table of Contents

1. PRONTO Trial Group....................4
The PRONTO Study Team (Collaborative Authorship)....................4
Table s1 PRONTO Trial Sites....................8
2. Methods....................9
Figure s1 Trial Schema (Format Supplied to Sites)....................9
s2.1 Deferred consent procedures....................10
s2.2 Complier Average Causal Effect (CACE) analysis....................10
Table s2 Decision matrix for co-primary outcomes....................11
3. Results....................12
Table s3 Full List of Participant Demographics and Baseline Characteristics (Primary Analysis Population) 12
Table s4 Participant Demographics and baseline Characteristics (All Participants)....................14
Table s5 Protocol non-compliances....................15
Table s6 Co-primary outcome analysis....................16
Table s7 Subgroup analysis of Co-Primary Outcomes (primary analysis population)....................16
Table s8 28-day mortality by arm for those who received a positive COVID-19 test result within 5 days of ED admission....................16
Figure s2 Forest Plot of Subgroup Analysis for IV Antibiotics Initiation at 3 hours (Co-Primary Outcome). . 17
Figure s3 Forest Plot of Subgroup analysis for 28 Day Mortality (Co-Primary Outcome)....................18
Table s9 Complete case analysis of the co-primary outcomes....................19
Table s10 Co-primary analyses after multiple imputation using chained equations....................19
s3.1 Interim analysis....................20
Table s11 Planned Interim Analyses after 43% of participants recruited....................20
Table s12 Unplanned Interim Analyses requested by IDMC after 57% of participants recruited....................20
Table s13 Mortality to Day 90 in withdrawn subjects....................20
Table s14 NEWS2 score by PCT score in the PCT-guided care arm....................21
Table s15 NEWS2 score by PCT-guided algorithm risk score....................21
Table s16 Post Hoc subgroup analysis of Index of Multiple Deprivation (IMD) score on coprimary outcomes (primary analysis population)....................22
Figure s4 Forest Plot of Subgroup Analysis for 28-day mortality by IMD decile....................22
Figure s5 Histogram of days on any antibiotics over first 28 days (zero days included)....................23
Figure s6 Histogram of days on IV antibiotics over first 28 days (zero days included)....................23
Figure s7 Histogram of days on broad spectrum (Watch/Reserve) antibiotics over first 28 days (zero days included)....................24
Table s17 Adverse Events by Arm....................25
Table s18 Serious Adverse Events by Arm....................25
Table s19 Participant timeline/pathway (primary analysis population)....................26
Table s20 Participant timeline/pathway (all participants)....................26
Table s21 Type of antibiotic received between 0-12 hours from triage assessment (primary analysis population)....................27
Table s22 Type of antibiotic received between 0-12 hours from triage assessment (all participants)....................27
Table s23 Top ten antibiotics of those initiated within 12-hours from triage....................27
Table s24 28-day mortality by type of antibiotic received between 0-12 hours from triage assessment (all participants)....................28
Figure s8 Change in A) Proportion of Antibiotic at 12 hours B) 28 Day Mortality by Antibiotic Route at 12 hours and Baseline NEWS2....................28

Table s26 Oxygen therapy (secondary outcome analysis population) 29
Table s28 Subgroup analysis for the grade of the most senior clinician conducting assessment (primary analysis population) 29
Figure s9 Change in Diagnosis Category from Initial Assessment to Final Diagnosis by Arm 30
Table s29 Initial and final diagnosis by trial arm (primary analysis population) 31
Table s30 Blood cultures taken within 24 hours of admission 31
Table s31 28-day mortality by final diagnosis (primary analysis population) (where final diagnosis was a single non-infectious diagnosis) 32
Table s32 28-day mortality by final diagnosis (primary analysis population) (where final diagnosis had more than one diagnosis listed and included a non-infectious diagnosis) 32
Table s33 90-day mortality by final diagnosis (all participants) (where final diagnosis was a single non-infectious diagnosis) 32
Table s34 90-day mortality by final diagnosis (all participants) (where final diagnosis had more than one diagnosis listed and included a non-infectious diagnosis) 32
Table s35 Site level heterogeneity by estimating the variance of the random intercept for site in the co-primary outcome models (primary analysis population) 33
4. Supplementary Material References 33
Appendix A Participant Informed Consent Form 34
Appendix B Statistical Analysis Plan 35

1. PRONTO Trial Group

The PRONTO Study Team (Collaborative Authorship)

PARTNER: University of Liverpool, UK (Sponsor)
Co-Chief Investigator: Neil French[1]

Co-Investigator: Enitan D Carrol[1,2,3]
Co-Investigator: David Taylor-Robinson[4]
Co-Investigator: Stephen Aston[5,6]
Co-Investigator: Ingeborg Welters[7]

Author Affiliations:
1. Department of Clinical Infection, Microbiology and Immunology, Institute of Infection, Veterinary and Ecological Sciences, University of Liverpool, Liverpool, UK.
2. Department of Infectious Diseases, Alder Hey Children's NHS Foundation Trust, East Prescot Road, Liverpool, UK.
3. NIHR Alder Hey Clinical Research Facility, Alder Hey Children's Hospital, Liverpool, UK
4. Department of Public Health, Policy and Systems, University of Liverpool, Liverpool, UK
5. Institute of Systems, Molecular and Integrative Biology, University of Liverpool, UK
6. Liverpool University Hospitals, NHS Foundation Trust, Liverpool, UK
7. Institute for Life Course and Medical Sciences, University of Liverpool, Liverpool, UK

PARTNER: Cardiff University, UK (Coordinating Centre)
Study Lead: Emma Thomas-Jones[1]
Senior Trial Manager: Joanne Euden[1]
Trial Managers: Wakunyambo Maboshe[1]
Data Manager: Stephanie Gilbert[1]
Lead Statistician: Philip Pallmann[1]
Statisticians: Abin Thomas[1], Jennifer Condie[1]
Qualitative Researchers: Lucy Brookes-Howell,[1] Jackie Hughes,[1] Sarah Milosevic,[1]
Database Developer: Sam Clarkstone[1]
Research Administrators: Mahuampi Perez-Alijas,[1] Lena Meister,[1] Alexandra Rollinson-Salter[1]
Co-investigator: Kerenza Hood[2]
Author Affiliations:
1. Centre for Trials Research, College of Biomedical Life Sciences, Cardiff University, Neuadd Meirionnydd, Heath Park, Cardiff, UK.
2. College of Biomedical Life Sciences, Cardiff University, Cardiff, UK.

PARTNER: London School of Hygiene and Tropical Medicine, UK (Health Economic Analysis)
Lead Health Economist and co-Investigator: Luke Vale[1]
Health Economists: Gabriella Culina-Jones[1], David Lugo Palacios,[1]
Author Affiliations:
1. London School of Hygiene and Tropical Medicine, Keppel Street, London, UK

PARTNER: Sheikh Shakhbout Medical City, UAE
Co-investigator: Emmanuel Nsutebu
Author Affiliations:
1. Sheikh Shakhbout Medical City, Abu Dhabi, United Arab Emirates

PARTNER: NHS England and NHS Improvement, UK
Co-investigator: Philip Howard[1]
Author Affiliations:
1. NHS England and NHS Improvement, North-East and Yorkshire, UK

PARTNER: Hampshire Hospitals NHS Foundation Trust, UK
Co-investigator: Matthew Inada-Kim[1]
Author Affiliations:
1. Department of Acute Medicine, Hampshire Hospitals NHS Foundation Trust, Basingstoke, UK

PARTNER: Patient and Public Representative, UK
Co-investigator: Julie Carman[1]

Author Affiliations:
1. UK Sepsis Trust (Volunteer), Walsall, UK

Participating NHS Trusts:
Partner: Liverpool University Hospitals NHS Foundation Trust, UK (Lead Recruiting Site)
Co-Chief Investigator: Stacy Todd[1]
Principal Investigator and co-Investigator: Stephen Aston[1,2]
Research Group (recruitment and data collection – in alphabetical order): Mary Brodsky,[1] Dorothy Culpa,[1] Rebecca Denton,[1] Allayna Doherty,[1] Amy Doyle,[1] Claire Duffy,[1] Jennifer Entwistle,[1] Rachael Fergusson,[1] Louise Fowler,[1] Aaron Geoghegan,[1] Sharon Glynn,[1] Jesica Goncalves,[1] Alvyda Gureviciute,[1] Bindu Harikumar,[1] Megan Howard,[1] Emily Llangrath,[1] Clare Jones,[1] Oliver Jones,[1] Jack Maher,[1] Kate Maitland,[1] Danielle Mclaughlan,[1] Terrence McLoughlin,[1] Nathalie Nicholas,[1] Wafae Ouarch,[1] Ian Quayle,[1] Emma Richardson,[1] Violet Ruhumbika,[1] Deborah Scanlon,[1] Nathan Sullivan,[1] Eleanor Taylor-Barr,[1] Shay Willoughby.[1]
Author Affiliations:
1. Royal Liverpool University Hospital, Liverpool University Hospitals NHS Foundation Trust, Liverpool, UK
2. Institute of Systems, Molecular and Integrative Biology, University of Liverpool, Liverpool, UK

Partner: Hull University Teaching Hospitals NHS Trust, UK (Lead Recruiting Site)
Principal Investigator: Gavin Barlow[1,2]
Associate Principal Investigator: Fahed Bangash,[1]
Research Group (recruitment and data collection – in alphabetical order): Nathan Blott,[1] Llucia Cabral-Ortega,[1] Katie Drury,[1] Angela Good,[1] Donna Gotts,[1] Rachel Harris,[1] Matthew Hines,[1] Claire Jones,[1] Diana Kluczna,[1] Patrick Lillie,[1] Rosa McGing,[1] Victoria Martinson,[1] Isabel Mortimer,[1] Mohammed Muddassir,[1] Tanaraj Perinpanathan,[1] Alexander Richards,[1] Charlotte Smith,[1] Debra Smith,[1] Neil Smith,[1] William Smith,[1] Elizabeth Stones,[1] Joseph Suich,[1] Thomas Taynton,[1] Gemma Walker,[1] Karen Winter,[1]
Author Affiliations:
1. Hull Royal Infirmary, Hull University Teaching Hospitals NHS Trust, Hull, UK
2. Hull York Medical School, University of York, Hull, UK

Partner: Leeds Teaching Hospitals NHS Trust, UK (Lead Recruiting Site)
Principal Investigator and co-Investigator: Fiona McGill[1]
Associate Principal Investigator: Gitanjali Sharma,[1]
Research Group (recruitment and data collection – in alphabetical order): Angelique Aspinwall,[1] Showna Benjamin,[1] Rebekah Burnham,[1] Emma Carter,[1] Kiran Chana,[1] Sam Charlton,[1] Suzie Colquhoun,[1] Abu Hassan,[1] Taj Hassan,[1] Honorine Jobain,[1] Gary Lamont,[1] Tadas Mazeika,[1] Michael Padden,[1] Isabelle Rogers,[1] Gaushiya Saiyad,[1] Razan Saman,[1] Wioletta Sobacka,[1] Oliver Wordsworth,[1] Robert White,[1]
Author Affiliations:
1. St James's University Hospital, Leeds Teaching Hospitals NHS Trust, Leeds, UK

Partner: Portsmouth Hospitals University NHS Trust, UK (Lead Recruiting Site)
Principal Investigator and co-Investigator: Paul Schmidt[1]
Research Group (recruitment and data collection – in alphabetical order): Sean Beech,[1] Gyles Brown,[1] Preeya Chauhan,[1] Helen Claridge,[1] Zoe Daly,[1] Gemma Dixon,[1] Sally Gosling,[1] Andrew Gribbin,[1] Karen Hudson,[1] Claudia Lameirinhas,[1] Angela Nown,[1] Steve Rose,[1] Kerrie Scott,[1] Susan Taylor,[1] Chinazom Ugwueze,[1]

Author Affiliations:
1. Queen Alexandra Hospital, Portsmouth Hospitals University NHS Trust, Portsmouth, UK

Partner: University Hospitals Sussex NHS Foundation Trust, UK (Lead Recruiting Site)
Principal Investigator and co-Investigator: Martin Llewelyn[1,2,3]
Principal Investigator: Chetan Trivedy[1]
Research Group (recruitment and data collection – in alphabetical order): Raquel Akieme,[1,2] Dina Alimari,[1] Sara Appasamy,[1] Geraldine Bassett,[1] Carla Clegg,[1,2] Raghavendra Devisetty,[2] Mohamed Elouby,[1] Kay Franklin,[1] Jane Gaylard,[1,2] Monica Gil,[1,2] Andrew McGregor,[1] Justyna Nowak,[2] Laura Ortiz-Ruit de Gordoa,[1] Caroline Paley,[1] Maya Perry,[1] Denise Skinner,[1,2] Nicola Skipper,[1] Keely Stewart,[1,2] Liam Todd,[1,2] Penny Traviss,[1] Elohor Uwadiogbu,[1,2] Alan Wallace,[1]

Author Affiliations:
1. Royal County Sussex Hospital, University Hospitals Sussex NHS Foundation Trust, Brighton, UK
2. Princess Royal Hospital, University Hospitals Sussex NHS Foundation Trust, Brighton, UK
3. Brighton and Sussex Medical School, University of Sussex, Brighton, UK

Cardiff & Vale University Health Board, UK (recruiting Site)
Principal Investigator: Jonathan Underwood[1]
Research Group (recruitment and data collection – in alphabetical order): Jade Cole,[1] Nicholas Manville,[1] Non Smith,[1] Lauren Thomas,[1] Rhys Thomas,[1]
Author Affiliations:
1. University Hospital of Wales, Cardiff & Vale University Health Board, Cardiff, UK

Cambridge University Hospitals NHS Foundation Trust (recruiting Site)
Principal Investigator: Rajeev Madan,[1]
Associate Principal Investigator: Raiiq Ridwan,[1]
Research Group (recruitment and data collection – in alphabetical order): Caitlin Adeniyi-Jones,[1] Audrey Campbell,[1] Emma Clark,[1] Katie Coupe,[1] Beverley Dickinson,[1] Georgina Gosney,[1] Susie Hardwick,[1] Teresa Lareza,[1] Pragya Mallick,[1] Kerry Meynell,[1] Alessandra Tidona,[1]

Author Affiliations:
1. Addenbrookes Hospital, Cambridge University Hospitals NHS Foundation Trust, Cambridge, UK

Gateshead Health NHS Foundation Trust (recruiting Site)
Principal Investigator: Rajeev Sharma,[1]
Research Group (recruitment and data collection – in alphabetical order): Maria Bokhari,[1] James Harvey,[1] Lillian Lee,[1] Mehr Mehmood,[1] Beverley McClelland,[1] Jenny Ritzema,[1] Helen Wild,[1] Ann Wilson,[1]
Author Affiliations:
1. Queen Elizabeth Hospital, Gateshead Health NHS Foundation Trust, Gateshead, UK

Gloucestershire Hospitals NHS Foundation Trust (recruiting Site)
Principal Investigator: Emma Tilley,[1]
Research Group (recruitment and data collection – in alphabetical order): Gemma Baldwin,[1] Jennifer Bates,[1] Madeleine Benson,[1] Michael Connelly,[1] Hary Coulton,[1] Rebekah Da Silva Teixeira,[1] Isabel Evans,[1] Jennifer Griffiths,[1]Jessica Hassell,[1] Paula Hilltout,[1] Anne-Marie Joyce,[1] Jennie Lowdell,[1] Amanda Tyler,[1] Nick Vallotton,[1] Deborah Ward,[1] Carys Whitby,[1]

Author Affiliations:
1. Gloucestershire Royal Hospital, Gloucestershire Hospitals NHS Foundation Trust, Gloucester, UK

Northern Care Alliance NHS Foundation Trust (recruiting Site)
Principal Investigator: John-Paul Williamson,[1]
Research Group (recruitment and data collection – in alphabetical order): Jack Haslam,[1] Louise Howard-Sandy,[1] Grainne O'Connor,[1] Georgia Moth,[1] Sheila Munt,[1] Jennifer Philbin,[1] Sarah Warran,[1] Sarah Winnard,[1]
Author Affiliations:
1. The Royal Oldham Hospital, Northern Care Alliance NHS Foundation Trust, Oldham, UK

Oxford University Hospitals NHS Foundation Trust, UK (recruiting Site)
Principal Investigators: Tanya Baron,[1]
Associate Principal Investigator: Muhammad Faisal,[1]
Research Group (recruitment and data collection – in alphabetical order): Sally Beer,[1] Joyce Chan,[1] Phoebe Cherrington-Walker,[1] Benjamin Clare-Gray,[1] George Corby,[1] Celia Diaz-Uribe,[1] Karen Dineen,[1] Abdala Espinosa,[1] Alexis Espinosa,[1] Dominique Georgiou,[1] Katerina Gramm,[1] Elizabeth Hatton,[1] Ralph Hovet,[1] Aimee Jeffs,[1] Jacinta Kynaston,[1] Hanxiao Li,[1] Martina Iorio,[1] Rosie Lynch,[1] Rufino Magallano,[1] Jose Martinez,[1] Anna Mikanik,[1] Alison Monk,[1] Jane Okumoku,[1] Tine Panduro,[1] Tinelly Sambo,[1] Nayanika Sreejith,[1] Hannah Thraves,[1] Jack Wilson,[1] Joao Ziroldo,[1]

Author Affiliations:
1. John Radcliffe Hospital, Horton Hospital, Oxford University Hospitals NHS Foundation Trust, Oxford, UK

Royal Cornwall Hospitals NHS Trust (recruiting Site)
Principal Investigator: Mark Jadav,[1]
Research Group (recruitment and data collection – in alphabetical order): Benita Adams,[1] Jane Agard,[1] Monica Ayestaran,[1] Charlotte Barker-Kirby,[1] Sharon Botfield,[1] Charlotte Bowyer,[1] Jenna Datson,[1] Eve Fletcher,[1] Chrissie Hall,[1] Fiona Hammonds,[1] Claire James,[1] Lily Jenkins,[1] Sandra Kessly,[1] Lindsay Knight,[1] Catherine Lee-Kim-Koon,[1] Cathal Murphy,[1] Tara Murray,[1] Kate Ralph,[1] Peter Thomas,[1] Sally Thomas,[1] Leanne Trehouan,[1]

Author Affiliations:
1. Royal Cornwall Hospital, Royal Cornwall Hospital NHS Trust, Truro, UK

South Tees Hospitals NHS Foundation Trust, UK (recruiting Site)

Principal Investigator: Richard Procter,[1]
Associate Principal Investigator: Jonathan Bennett,[1]
Research Group (recruitment and data collection – in alphabetical order): Kerry Colling,[1] Abigail List,[1] Joanne Morley,[1] Tracy Ruddick,[1] Dean Wilkinson,[1]
Author Affiliations:
1. The James Cook University Hospital, South Tees NHS Foundation Trust, Middlesbrough, UK

University Hospitals of Derby and Burton NHS Foundation Trust, UK (recruiting Site)

Principal Investigator: Andrew Tabner[1,2]
Co-Principal Investigator: Graham Johnson[1, 2]
Research Group (recruitment and data collection – in alphabetical order): Suzanna Ballard,[1] Elisha Cousins,[1] Alison Fletcher,[1,2] Charlotte Griffiths,[1] Paolyn Guiling,[1] Gareth Hughes,[1,2] Ainsley MacShannon,[1,2] Alison Matthews,[1,2] Lucy-May Moulden,[1] Alba Roberts,[1] Alison Rockey,[1] Hannah Scrafton,[1] Thomas Ward,[1] Lianne Wright,[1] Heather Wroblewski,[1] Marcelina Zawadzka,[1]

Author Affiliations:
1. Royal Derby Hospital, University Hospitals of Derby and Burton NHS Foundation Trust, Derby, UK
2. Queens's Hospital Burton, University Hospitals of Derby and Burton NHS Foundation Trust, Derby, UK
3. School of Medicine, University of Nottingham, Queen's Medical Centre, Nottingham, UK

The Princess Alexandra Hospital NHS Trust, UK (recruiting Site)

Principal Investigator Roberta Branisteanu[1]
Co-Principal Investigator: Andrea Annoni[1]
Research Group (recruitment and data collection – in alphabetical order): Imran Azeez,[1] Bibi Badal,[1] Martin Bailey,[1] Louise Barnard,[1] Joyce Benny,[1] Ayeda Emran,[1] Joanne Finn,[1] Anna Geevasghese,[1] Steven Hart,[1] Karen Ixer,[1] Rezma Miah,[1]Victoria Mackenzie,[1] Hannah Mugford,[1] Patricia Nabayego,[1] Julie Payne,[1] Isma Rafiq,[1] Camilla Ramus,[1] Preyeta Saha,[1] Nazreen Sahbeer,[1] Ezaldeen Shareah,[1] Abigail Smith,[1] Jenny Styles,[1] Jane Smith,[1] Debbie Thomas,[1] Shreevatsa Udupa,[1] Abdalla Wheiba,[1]

Author Affiliations:
1. Princess Alexandra Hospital, The Princess Alexandra Hospital NHS Trust, Harlow, UK

University Hospitals Dorset NHS Foundation Trust, UK (recruiting Site)

Principal Investigator: David Martin[1,2]
Research Group (recruitment and data collection – in alphabetical order): Chloe Bascombe,[1] Nina Barratt,[1] Matthew Bayliss,[1,2] Faith Beecham,[1] Karen Chapman,[1] Hayley Chapple,[1] Gary Cumberbatch,[1,2] Yasmin De'ath,[1,2] Rebecca Fletcher,[1] Annette Fraine,[1] Kathleen Horan,[1] Charlotte Humphrey,[1,2] Elizabeth Hurdidge,[1] Emma Langridge,[1,2] Cheryl Lindsay,[1] Helen McHale,[1] Rebecca Miln,[1,2] Mirela Mukaj,[1] Claire Osey,[1,2] Karen O'Toole,[1] Jodie Pinnock,[1,2] Sally Pitts[1] Javen Ramsami,[1] Suzanne Roffe,[1] Joanna Samways,[1] Sarah Savage,[1,2] Natasha Tamoidi,[1] Heather Tiller,[1] Rebecca Troke,[1] Luke Vamplew,[1] James White,[1] Annamaria Wilce,[1]
Author Affiliations:
1. Royal Bournemouth Hospital, University Hospitals Dorset NHS Foundation Trust, Poole, UK
2. Poole Hospital, University Hospitals Dorset NHS Foundation Trust, Poole, UK

Wrightington, Wigan & Leigh Teaching Hospitals NHS Foundation Trust (recruiting Site)

Principal Investigator: Ayaz Abbasi,[1]
Research Group (recruitment and data collection – in alphabetical order): Victor Ameh,[1] Joshua Cooper,[1] Sarah Liderth,[1] Emma Robinson,[1] Natalia Waddington,[1]
Author Affiliations:
1. The Royal Albert Edward Infirmary, Wrightington, Wigan & Leigh Teaching Hospitals NHS Foundation Trust, Wigan, UK

Table s1 PRONTO Trial Sites

Site	Number of Recruits
University Hospitals of Derby and Burton NHS Foundation Trust – Royal Derby Hospital	1031
Liverpool University Hospitals NHS Foundation Trust - Royal Liverpool University Hospital	946
Leeds Teaching Hospitals NHS Trust– St James's University Hospital	636
Oxford University Hospitals NHS Foundation Trust – John Radcliffe Hospital	578
University Hospitals Sussex NHS Foundation Trust - Royal Sussex County Hospital	492
Cambridge University Hospitals NHS Foundation Trust – Addenbrookes Hospital	355
Portsmouth Hospitals University NHS Trust - Queen Alexandra Hospital	347
Cardiff & Vale University Health Board - University Hospital of Wales	276
Hull University Teaching Hospitals NHS Trust - Hull Royal Infirmary	243
Gloucestershire Hospitals NHS Foundation Trust – Gloucestershire Royal Hospital	232
Royal Cornwall Hospital NHS Trust - Royal Cornwall Hospital	163
The Princess Alexandra Hospital NHS Trust - Princess Alexandra Hospital	158
University Hospitals Dorset NHS Foundation Trust – Royal Bournemouth Hospital	149
South Tees Hospitals NHS Foundation Trust - James Cook University Hospital	123
University Hospitals Dorset NHS Foundation Trust - Poole Hospital	105
Gateshead Health NHS Foundation Trust – Queen Elizabeth Hospital	104
University Hospitals of Derby and Burton NHS Foundation Trust - Queen's Hospital Burton	83
University Hospitals Sussex NHS Foundation Trust - Princess Royal Hospital	54
Northern Care Alliance NHS Foundation Trust - Royal Oldham Hospital	43
Wrightington, Wigan & Leigh Teaching Hospitals NHS Foundation Trust – Royal Albert Edward Infirmary	1

2. Methods

Figure s1 Trial Schema (Format Supplied to Sites)

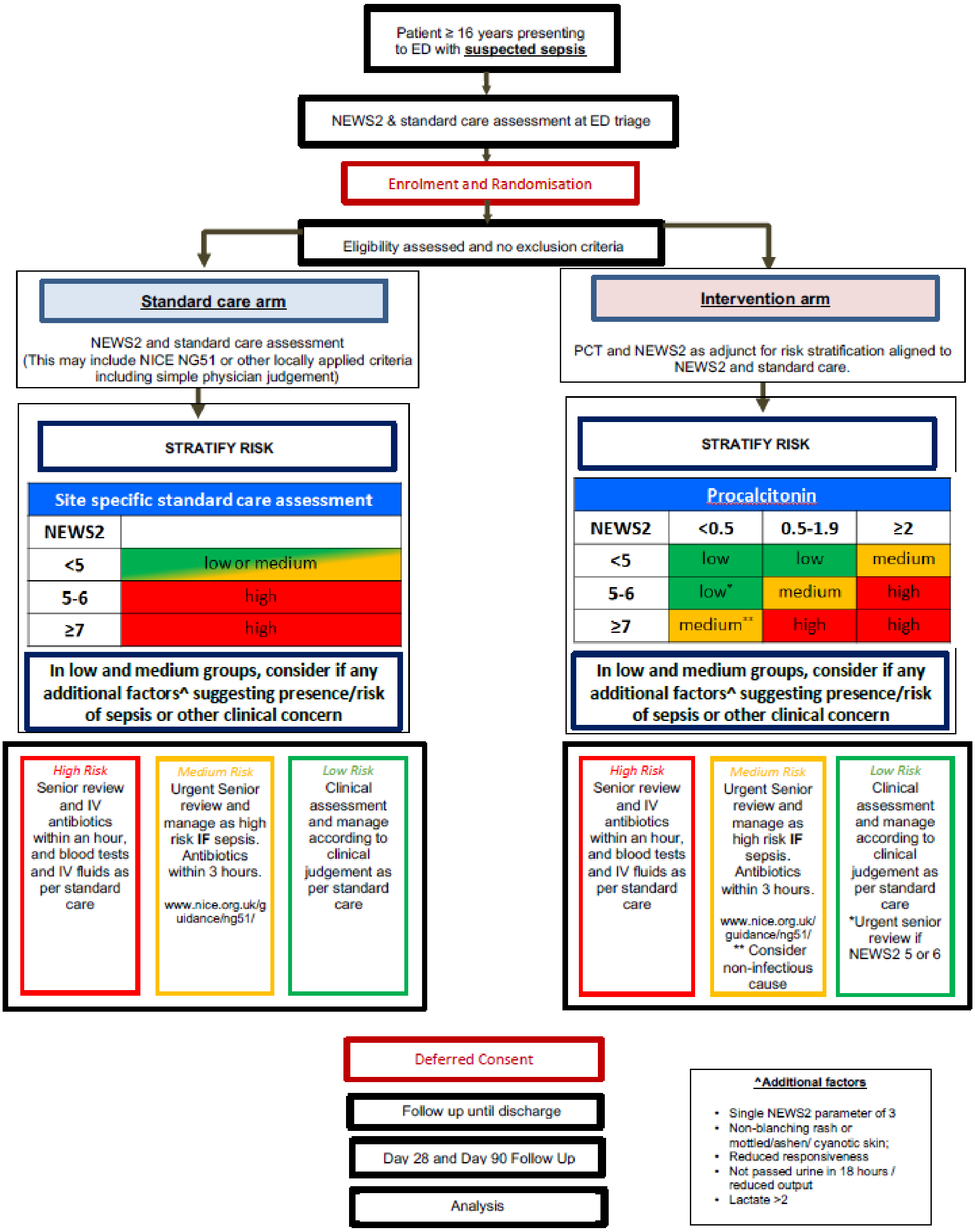

s2.1 Deferred consent procedures

In England and Wales, the law allows adults who lack capacity to take part in emergency research without prior consent from a legal representative or consulting others, if certain conditions are met (Mental Capacity Act (MCA) 2005).[1] Patients were informed that the trial was ongoing but a lengthy consent discussion was not held. Patients (or their consultee) could decline to join the trial at this stage. Following randomisation an approach to obtain written informed consent was made as soon as practicably feasible, ideally within 72 hours. A tiered consent model was used to allow participants to consent to different aspects of the trial. Patients who did not consent were withdrawn. Patients who were unable to consent for themselves due to a lack of mental capacity could still be included in the trial following discussion with either a personal or, in exceptional circumstances, a nominated consultee.[2]

s2.2 Complier Average Causal Effect (CACE) analysis

A supplementary Complier Average Causal Effect (CACE) analysis estimated the difference in outcomes between participants whose treating clinicians complied with the intervention and those whose treating clinicians would have complied had they been allocated to the intervention. Randomisation ensured that characteristics predicting compliance were balanced across arms, so the characteristics that predict compliance in the intervention arm could be used to infer likely compliance behaviour (on the clinicians' part) in the control arm.

To do this, we used a latent class approach based on generalised structural equation modelling as implemented in the Stata command gsem[20]. This method identified unobserved subgroups (latent classes), including compliers (i.e. those whose clinicians would always follow the allocated intervention, regardless of whether that is PCT or control), non-compliers (i.e. those whose clinicians would never follow the allocated intervention), and 'never-users' (i.e. those who would never have a PCT test done), based on observed covariates and intervention use in the intervention arm. These latent classes were then used to estimate the effect of the intervention among compliers, while accounting for unobserved compliance behaviour in the control arm.

We conducted three separate CACE analyses based on the following definitions of analysis populations depending on the level of compliance to the PCT-guided algorithm:

- Patients randomised to PCT-guided care in whom a PCT test was done, and a PCT result was available
- Patients randomised to PCT-guided care in whom a PCT test was done, a PCT result was available, and the clinician had seen the PCT result
- Patients randomised to PCT-guided care in whom a PCT test was done, a PCT result was available, the clinician had seen the PCT result and followed the recommendation arising from the algorithm risk score.

Table s2 Decision matrix for co-primary outcomes

	Reduced antibiotic initiation	Same or more antibiotic initiation
**Decreased mortality**	Effective	Effective
**Equivalent mortality**	Effective	Not effective
**Increased mortality**	Not effective/harmful	Not effective/harmful

3. Results

Table s3 Full List of Participant Demographics and Baseline Characteristics (Primary Analysis Population)

		PCT	Usual care
**N**		2738/5453 (50·2%)	2715/5453 (49·8%)
**Ethnicity**	White	2314/2738 (84.5%)	2314/2715 (85.2%)
	White English/Welsh/Scottish/Northern Irish/British	2258/2738 (82.5%)	2252/2715 (82.9%)
	White Irish	10/2738 (0.4%)	15/2715 (0.6%)
	Gypsy or Irish Traveller	0/2738 (0.0%)	1/2715 (0.0%)
	Any other White background	46/2738 (1.7%)	46/2715 (1.7%)
	Mixed	17/2738 (0.6%)	17/2715 (0.6%)
	White and Black Caribbean	3/2738 (0.1%)	5/2715 (0.2%)
	White and Black African	4/2738 (0.1%)	3/2715 (0.1%)
	White and Asian	3/2738 (0.1%)	2/2715 (0.1%)
	Any other mixed/multiple ethnic background	7/2738 (0.3%)	7/2715 (0.3%)
	Asian	64/2738 (2.3%)	45/2715 (1.7%)
	Indian	19/2738 (0.7%)	13/2715 (0.5%)
	Pakistani	20/2738 (0.7%)	14/2715 (0.5%)
	Bangladeshi	4/2738 (0.1%)	4/2715 (0.1%)
	Chinese	6/2738 (0.2%)	4/2715 (0.1%)
	Any other Asian background	15/2738 (0.5%)	10/2715 (0.4%)
	Black	27/2738 (1.0%)	23/2715 (0.8%)
	African	14/2738 (0.5%)	13/2715 (0.5%)
	Caribbean	4/2738 (0.1%)	1/2715 (0.0%)
	Any other Black/African/Caribbean background	9/2738 (0.3%)	9/2715 (0.3%)
	Other	36/2738 (1.3%)	28/2715 (1.0%)
	Arab	5/2738 (0.2%)	0/2715 (0.0%)
	Any other ethnic group	31/2738 (1.1%)	28/2715 (1.0%)
	Missing/not declared	280/2738 (10.2%)	288/2715 (10.6%)
**Number of Listed Comorbidities**	0	547/2738 (20.0%)	512/2715 (18.9%)
	1	559/2738 (20.4%)	563/2715 (20.7%)
	2+	1592/2738 (58.1%)	1611/2715 (59.3%)
	Missing	40/2738 (1.5%)	29/2715 (1.1%)
**Comorbidities**	Diabetes Mellitus	623/2738 (22.8%)	651/2715 (24.0%)
	COPD	624/2738 (22.8%)	615/2715 (22.7%)
	Heart Failure	338/2738 (12.3%)	378/2715 (13.9%)
	Moderate to severe CKD (Grade III or above)	371/2738 (13.6%)	372/2715 (13.7%)
	Ischaemic Heart Disease	287/2738 (10.5%)	314/2715 (11.6%)
	Stroke or TIA	282/2738 (10.3%)	292/2715 (10.8%)
	Dementia	253/2738 (9.2%)	266/2715 (9.8%)
	Physical Disability	249/2738 (9.1%)	239/2715 (8.8%)
	Solid Tumour Malignancy	206/2738 (7.5%)	204/2715 (7.5%)
	Myocardial Infarction	180/2738 (6.6%)	187/2715 (6.9%)
	Peripheral Vascular Disease	110/2738 (4.0%)	124/2715 (4.6%)
	Connective Tissue Disease	101/2738 (3.7%)	88/2715 (3.2%)
	Liver Disease	75/2738 (2.7%)	64/2715 (2.4%)
	Learning Disability	41/2738 (1.5%)	41/2715 (1.5%)
	Haematological malignancy	40/2738 (1.5%)	41/2715 (1.5%)

	Peptic Ulcer Disease	41/2738 (1.5%)	34/2715 (1.3%)
	Hemiplegia	24/2738 (0.9%)	32/2715 (1.2%)
	HIV	9/2738 (0.3%)	11/2715 (0.4%)
**Managed as suspected COVID-19**	No	1870/2738 (68.3%)	1874/2715 (69.0%)
	Yes	283/2738 (10.3%)	267/2715 (9.8%)
	Missing	585/2738 (21.4%)	574/2715 (21.1%)
**Previous positive COVID-19 test result at any time**	Yes	475/2738 (17.3%)	475/2715 (17.5%)
	No	1929/2738 (70.5%)	1932/2715 (71.2%)
	Missing	334/2738 (12.2%)	308/2715 (11.3%)

Table s4 Participant Demographics and baseline Characteristics (All Participants)

		PCT	Usual care
**N**		3092/6119 (50.5%)	3027/6119 (49.5%)
**Age (years)**	Median [IQR]	72 [57, 82]	73 [58, 82]
**Gender (%)**	Male	1533/3092 (49.6%)	1519/3027 (50.2%)
	Female	1557/3092 (50.4%)	1508/3027 (49.8%)
	Non-binary	1/3092 (0.0%)	0/3027 (0.0%)
	Missing	1/3092 (0.0%)	0/3027 (0.0%)
**Time between ED admission and triage assessment (hours)**	Median [IQR]	0.3 [0.1, 0.8]	0.3 [0.1, 0.7]
**Duration of symptoms prior to enrolment (hours)**	Median [IQR]	48 [20, 96]	48 [20, 96]
**NEWS2**	≤ 4	1148/3092 (37.1%)	1145/3027 (37.8%)
	5 – 6	834/3092 (27.0%)	805/3027 (26.6%)
	≥ 7	1110/3092 (35.9%)	1077/3027 (35.6%)
**Ethnicity**	White	2601/3092 (84.1%)	2554/3027 (84.3%)
	White English/Welsh/Scottish/Northern Irish/British	2540/3092 (82.2%)	2483/3027 (82.0%)
	White Irish	11/3092 (0.4%)	15/3027 (0.5%)
	Gypsy or Irish Traveller	0/3092 (0.0%)	1/3027 (0.0%)
	Any other White background	50/3092 (1.6%)	55/3027 (1.8%)
	Mixed	20/3092 (0.6%)	19/3027 (0.6%)
	White and Black Caribbean	4/3092 (0.1%)	5/3027 (0.2%)
	White and Black African	5/3092 (0.2%)	4/3027 (0.1%)
	White and Asian	4/3092 (0.1%)	3/3027 (0.1%)
	Any other mixed/multiple ethnic background	7/3092 (0.2%)	7/3027 (0.2%)
	Asian	68/3092 (2.2%)	51/3027 (16.7%)
	Indian	22/3092 (0.7%)	14/3027 (0.5%)
	Pakistani	20/3092 (0.7%)	16/3027 (0.5%)
	Bangladeshi	5/3092 (0.2%)	4/3027 (0.1%)
	Chinese	6/3092 (0.2%)	6/3027 (0.2%)
	Any other Asian background	15/3092 (0.5%)	11/3027 (0.4%)
	Black	28/3092 (0.9%)	23/3027 (0.8%)
	African	14/3092 (0.5%)	13/3027 (0.4%)
	Caribbean	5/3092 (0.2%)	1/3027 (0.0%)
	Any other Black/African/Caribbean background	9/3092 (0.3%)	9/3027 (0.3%)
	Other	39/3092 (1.3%)	37/3027 (1.2%)
	Arab	5/3092 (0.2%)	0/3027 (0.0%)
	Any other ethnic group	34/3092 (1.1%)	37/3027 (1.2%)
	Missing/Not declared	336/3092 (10.9%)	343/3027 (11.3%)
**History of oral antibiotics in the 14 days prior to admission**	No	2276/3092 (73.6%)	2236/3027 (73.9%)
	Yes	736/3092 (23.8%)	732/3027 (24.2%)
	Missing	80/3092 (2.6%)	59/3027 (2.0%)
**Comorbidities**	0	598/3092 (19.3%)	560/3027 (18.5%)

	1	621/3092 (20.1%)	622/3027 (20.6%)
	2+	1831/3092 (59.2%)	1815/3027 (60.0%)
	Missing	42/3092 (1.4%)	30/3027 (1.0%)
**Comorbidities**	Diabetes Mellitus	711/3092 (23.0%)	719/3027 (23.8%)
	COPD	704/3092 (22.8%)	694/3027 (22.9%)
	Moderate to severe CKD (Grade III or above)	422/3092 (13.7%)	416/3027 (13.7%)
	Heart Failure	380/3092 (12.3%)	416/3027 (13.7%)
	Stroke or TIA	336/3092 (10.9%)	335/3027 (11.1%)
	Ischaemic Heart Disease	332/3092 (10.7%)	345/3027 (11.4%)
	Physical Disability	295/3092 (9.5%)	271/3027 (9.0%)
	Dementia	284/3092 (9.2%)	307/3027 (10.1%)
	Solid Tumour Malignancy	240/3092 (7.8%)	241/3027 (8.0%)
	Myocardial Infarction	209/3092 (6.8%)	215/3027 (7.1%)
	Peripheral Vascular Disease	119/3092 (3.9%)	138/3027 (4.6%)
	Connective Tissue Disease	124/3092 (4.0%)	103/3027 (3.4%)
	Liver Disease (If yes, detail severity below)	86/3092 (2.8%)	72/3027 (2.4%)
	Learning Disability	47/3092 (1.5%)	53/3027 (1.8%)
	Haematological malignancy	48/3092 (1.6%)	44/3027 (1.5%)
	Peptic Ulcer Disease	44/3092 (1.4%)	35/3027 (1.2%)
	Hemiplegia	27/3092 (0.9%)	35/3027 (1.2%)
	HIV	11/3092 (0.4%)	12/3027 (0.4%)
**Charlson Comorbidity Index**	Mean (SD)	4.1 (2.7)	3.9 (2.7)
	Median [IQR]	4 [2, 6]	4 [2, 6]
**CRP levels (mg/L)**	Median [IQR]	70 [22, 156]	64 [22, 147]

Table s5 Protocol non-compliances

Non-compliance category	Categorisation of non-compliance	Total Number
Participant Consent	Deviation	15
Randomisation/registration	Deviation	7
Misconduct	Deviation	3
Data Collection	Deviation	1
Other	Deviation	4
**Total**		**30**

Table s6 Co-primary outcome analysis

	PCT (n=2738)	Usual care (n=2715)		Risk difference % (SE)	90% CI	P-value
**28-day mortality**	372/2738 (13·6%)	450/2715 (16·6%)	Primary	-3·12(0.94)	-4·68 to -1·57	0·0009
	367/2697 (13·6%)	439/2685 (16·4%)	Adjusted for covariates	-2·35 (0.94)	-3.85 to -0·86	0·0094
				**Risk difference % (SE)**	**95% CI**	**P-value**
**IV antimicrobial initiation at 3 hours**	1325/2738 (48·4%)	1308/2715 (48·2%)	Primary	-0·08 (0·013)	-2·58 to 2·42	0·95
	1310/2697 (48·6%)	1294/2685 (48·2%)	Adjusted for covariates	0·15 (0·013)	-2·37 to 2·66	0·91

SE = standard error, CI = confidence interval, OBF = O'Brien-Fleming. Analysis method: two-level logistic regression models. Covariates in primary models: baseline NEWS2 . Covariates in adjusted models: baseline NEWS2, age, gender, Charlson Comorbidity Index.

Table s7 Subgroup analysis of Co-Primary Outcomes (primary analysis population)

Outcome	Subgroups	n	LRT χ^2 (df)	p-value
**28-day mortality (non-inferiority)** [(a)]	Organ system of infection	4971	7.26 (8)	0.51
	Risk category based on NEWS2 score at baseline	5453	1.11 (2)	0.57
	Managed as suspected COVID-19 during admission	4294	1.00 (1)	0.32
	Positive COVID-19 test result +/- 5 days from admission*	-	-	-
	PCT machine used	5453	0.16 (1)	0.69
	Recruitment date	5453	0.93 (2)	0.63
	Level of ED crowding	4234	1.78 (1)	0.18
**IV antimicrobial initiation at 3 hours (superiority)**[(a)]	Organ system of infection	5006	12.38 (9)	0.19
	Risk category based on NEWS2 score at baseline	5453	2.18 (2)	0.34
	Managed as suspected COVID-19 during admission	4294	0.31 (1)	0.58
	Positive COVID-19 test result +/- 5 days from admission	5453	2.53 (1)	0.11
	PCT machine used	5453	0.98 (1)	0.32
	Recruitment date	5453	0.28 (2)	0.87
	Level of ED crowding	4234	0.80 (1)	0.37
**Analysis method: (a) multilevel logistic regression. Interaction tests by model comparison using likelihood ratio test. *Interaction test could not be performed due to no deaths being observed in one subgroup (see Supplementary Material Table s7).<br>Covariates in all models: baseline NEWS2.**				

Table s8 28-day mortality by arm for those who received a positive COVID-19 test result within 5 days of ED admission.

28-day mortality	Overall	PCT	Usual Care
**Yes**	4/125 (3.2%)	4/64 (6.3%)	0/61 (0.0%)
**No**	121/125 (96.8%)	60/64 (93.8%)	61/61 (100.0%)

Fisher's exact test two sided for association between intervention and death p=0.12.

Figure s2 Forest Plot of Subgroup Analysis for IV Antibiotics Initiation at 3 hours (Co-Primary Outcome).

The overall effect is plotted from the primary analysis outputs

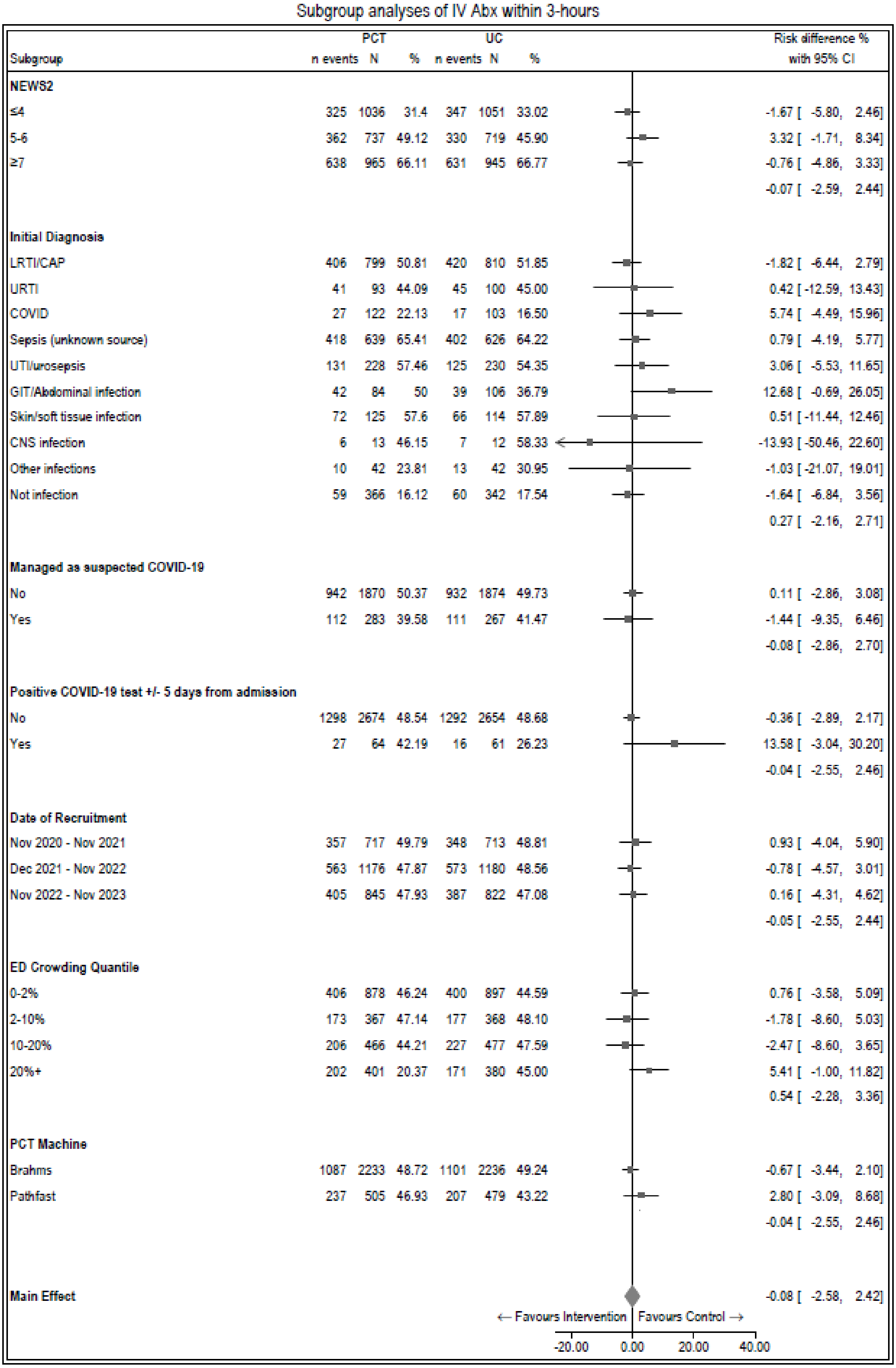

Figure s3 Forest Plot of Subgroup analysis for 28 Day Mortality (Co-Primary Outcome).
The overall effect is plotted from the primary analysis outputs

Subgroup analyses of 28-day mortality

Subgroup	PCT n events	PCT N	PCT %	UC n events	UC N	UC %	Risk difference % with 90% CI
**NEWS2**							
≤4	58	1036	5.60	65	1051	6.18	-0.58 [-2.41, 1.25]
5-6	79	737	10.72	105	719	14.60	-4.04 [-7.01, -1.07]
≥7	235	965	24.35	280	945	29.63	-5.22 [-8.51, -1.93]
							-2.21 [-3.62, -0.80]
**Initial Diagnosis**							
LRTI/CAP	128	799	16.02	163	810	20.12	-4.90 [-8.05, -1.76]
URTI	7	93	7.53	4	100	4.00	5.20 [-1.16, 11.56]
COVID	10	122	8.20	11	103	10.68	-0.54 [-6.68, 5.60]
Sepsis (unknown source)	115	639	18.00	136	626	21.73	-3.85 [-7.40, -0.30]
UTI/urosepsis	15	228	6.58	16	230	6.96	-0.25 [-3.96, 3.46]
GIT/Abdominal infection	11	84	13.10	12	106	11.32	1.82 [-5.70, 9.34]
Skin/soft tissue infection	7	125	5.60	10	114	8.77	-3.00 [-8.45, 2.44]
Other infections	3	42	7.14	8	42	19.05	-13.00 [-26.28, 0.28]
Not infection	40	366	10.93	55	342	16.08	-5.36 [-9.45, -1.27]
							-2.77 [-4.31, -1.23]
**Managed as suspected COVID-19**							
No	257	1870	13.74	320	1874	17.08	-3.73 [-5.66, -1.80]
Yes	32	283	11.31	48	267	17.98	-6.80 [-11.60, -2.00]
							-4.16 [-5.95, -2.37]
**Positive COVID-19 test +/- 5 days from admission**							
No	368	2674	13.76	450	2654	16.96	-3.30 [-4.88, -1.72]
							-3.30 [-4.88, -1.72]
**Date of Recruitment**							
Nov 2020 - Nov 2021	94	717	13.11	109	713	15.29	-2.07 [-4.88, 0.73]
Dec 2021 - Nov 2022	140	1176	11.90	187	1180	15.85	-3.95 [-6.25, -1.66]
Nov 2022 - Nov 2023	138	845	16.33	154	822	18.73	-2.97 [-5.98, 0.04]
							-3.14 [-4.67, -1.61]
**ED Crowding Quantile**							
0-2%	97	878	11.05	125	897	13.94	-3.35 [-5.85, -0.85]
2-10%	42	367	11.44	58	368	15.76	-4.21 [-8.20, -0.23]
10-20%	51	466	10.94	74	477	15.51	-4.14 [-7.63, -0.65]
20%+	65	401	16.21	61	380	16.05	0.14 [-4.14, 4.42]
							-3.15 [-4.82, -1.49]
**PCT Machine**							
Brahms	298	2233	13.35	371	2236	16.59	-3.33 [-5.05, -1.61]
Pathfast	74	505	14.65	79	479	16.49	-2.27 [-5.82, 1.28]
							-3.13 [-4.68, -1.58]
**Main Effect**							-3.12 [-4.68, -1.57]

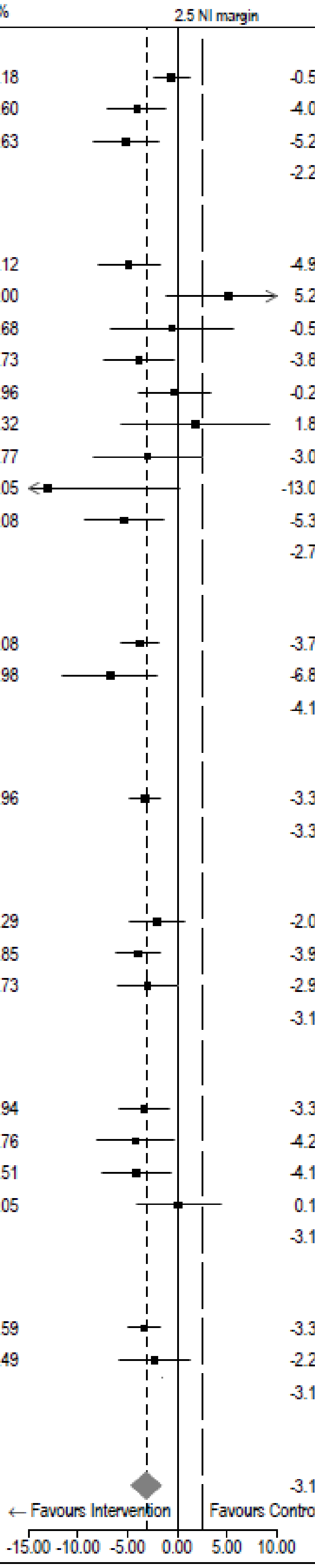

Table s9 Complete case analysis of the co-primary outcomes

		n	Risk difference (SE)	90% CI	P-value
**28-day mortality (non-inferiority)**	Primary	6013	-3.00 (0.91)	-4.50 to –1.50	0.001
	Adjusted for covariates	5940	-2.26 (0.88)	-3.71 to –0.82	0.0099
		**n**	**Risk difference (SE)**	**95% CI**	**P-value**
**IV antimicrobial initiation at 3 hours (superiority)**	Primary	5554	-0.22 (1.27)	-2.70 to 2.26	0.86
	Adjusted for covariates	5482	-0.02 (1.27)	-2.51 to 2.48	0.99

SE = standard error, CI = confidence interval. Analysis method: two-level logistic regression models. Covariates in primary models: baseline NEWS2. Covariates in adjusted models: baseline NEWS2, age, gender, Charlson Comorbidity Index.

Table s10 Co-primary analyses after multiple imputation using chained equations

		n	OR [95% CI]	P-value
**28-day mortality**	Primary	6013	0.782 [0.676 to 0.905]	0.001
	Adjusted	5940	0.818 [0.702 to 0.953]	0.010
	MI	6119	0.784 [0.678 to 0.906]	0.001
	MI adjusted	6119	0.805[0.693 to 0.936]	0.005
		**n**	**OR [95% CI]**	**P-value**
**IV antimicrobial initiation at 3 hours**	Primary	5554	0.990 [0.885 to 1.107]	0.86
	Adjusted	5482	0.999 [0.892 to 1.119]	0.99
	MI	6119	0.992 [0.881 to 1.116]	0.89
	MI adjusted	6119	0.997 [0.885 to 1.123]	0.96
MI = multiple imputation, OR = odds ratio, CI = confidence interval. Analysis method: two-level logistic regression models. Covariates in primary models: baseline NEWS2. Covariates in adjusted models: baseline NEWS2, age, gender, Charlson Comorbidity Index.				

s3.1 Interim analysis

The planned interim analysis was conducted after 3040 participants had been recruited and followed up for 28 days. A second, unplanned interim analysis was requested by the IDMC and conducted after 3973 participants had been recruited and followed up for 28 days. The interim analyses included options to stop the trial early for effectiveness using group-sequential boundaries based on OBF-type alpha spending.[3,4] We used a hierarchical approach (SAP, Appendix B) to recommend stopping for effectiveness if the PCT-guided assessment was non-inferior in terms of 28-day mortality and superior in terms of initiation of antibiotics, or if the PCT-guided assessment was superior in terms of 28-day mortality.

Table s11 Planned Interim Analyses after 43% of participants recruited

	PCT (n=1519)	Usual care (n=1521)	Risk difference % (SE)	CI	P-value
**28-day mortality**	204/1519 (13·4%)	269/1521 (17·7%)	-4.28 (1.28)	90% CI –6.38 to –2.18	0.0008
**IV antimicrobial initiation at 3 hours**	714/1519 (47·0%)	727/1521 (47·8%)	-1.11 (1.71)	95% CI –4.46 to 2.23	0.51

SE = standard error, CI = confidence interval. Analysis method: two-level logistic regression models. Covariates in model: baseline NEWS2.

Table s12 Unplanned Interim Analyses requested by IDMC after 57% of participants recruited

	PCT (n=1989)	Usual care (n=1984)	Risk difference % (SE)	CI	P-value
**28-day mortality**	283/1989 (14.2%)	357/1984 (18.0%)	-4.04 (1.14)	90% CI –5.91 to –2.17	0.0004
**IV antimicrobial initiation at 3 hours**	949/1989 (47.7%)	963/1984 (48.5%)	-1.23 (1.48)	95% CI -4.12, 1.67	0.41

SE = standard error, CI = confidence interval. Analysis method: two-level logistic regression models. Covariates in model: baseline NEWS2.

Table s13 Mortality to Day 90 in withdrawn subjects

	**Overall**	**PCT**	**Usual Care**
**Total number who did not consent to join the trial**	1548	809	739
**Deaths to day 90**	141 (9.1%)	74 (9.2%)	67 (9.1%)
**Unknown Status**	60 (3.9%)	-	-

Table s14 NEWS2 score by PCT score in the PCT-guided care arm

NEWS2 score	PCT <0.5	PCT 0.5-1.9	PCT >=2	PCT missing
<= 4	755/1148 (65.8%)	159/1148 (13.9%)	188/1148 (16.4%)	46/1148 (4.0%)
5-6	479/834 (57.4%)	127/834 (15.2%)	179/834 (21.5%)	49/834 (5.9%)
>=7	514/1110 (46.3%)	192/1110 (17.3%)	337/1110 (30.4%)	67/1110 (6.0%)

Table s15 NEWS2 score by PCT-guided algorithm risk score

NEWS2 score	Low	Medium	High	Missing
<= 4	914/1148 (79.6%)	188/1148 (16.4%)	0/1148 (0.0%)	46/1148 (4.0%)
5-6	479/834 (57.4%)	127/834 (15.2%)	179/834 (21.5%)	49/834 (5.9%)
>=7	0/1110 (0.0%)	514/1110 (46.3%)	529/1110 (47.7%)	67/1110 (6.0%)

Table s16 Post Hoc subgroup analysis of Index of Multiple Deprivation (IMD) score on coprimary outcomes (primary analysis population)

Outcome	n	LRT χ^2 (df)	p-value
28-day mortality [(a)]	4611	7.43 (1)	0.0064
IV antimicrobial initiation at 3 hours (superiority)[(a)]	4611	0.45 (1)	0.50
Analysis method: (a) multilevel logistic regression. Interaction tests by model comparison using likelihood ratio test. Covariates in all models: baseline NEWS2. IMD scores were derived using the English Indices of Deprivation 2019, with scores linked to participant postcodes using the UK Data Service's GeoConvert tool.			

Figure s4 Forest Plot of Subgroup Analysis for 28-day mortality by IMD decile

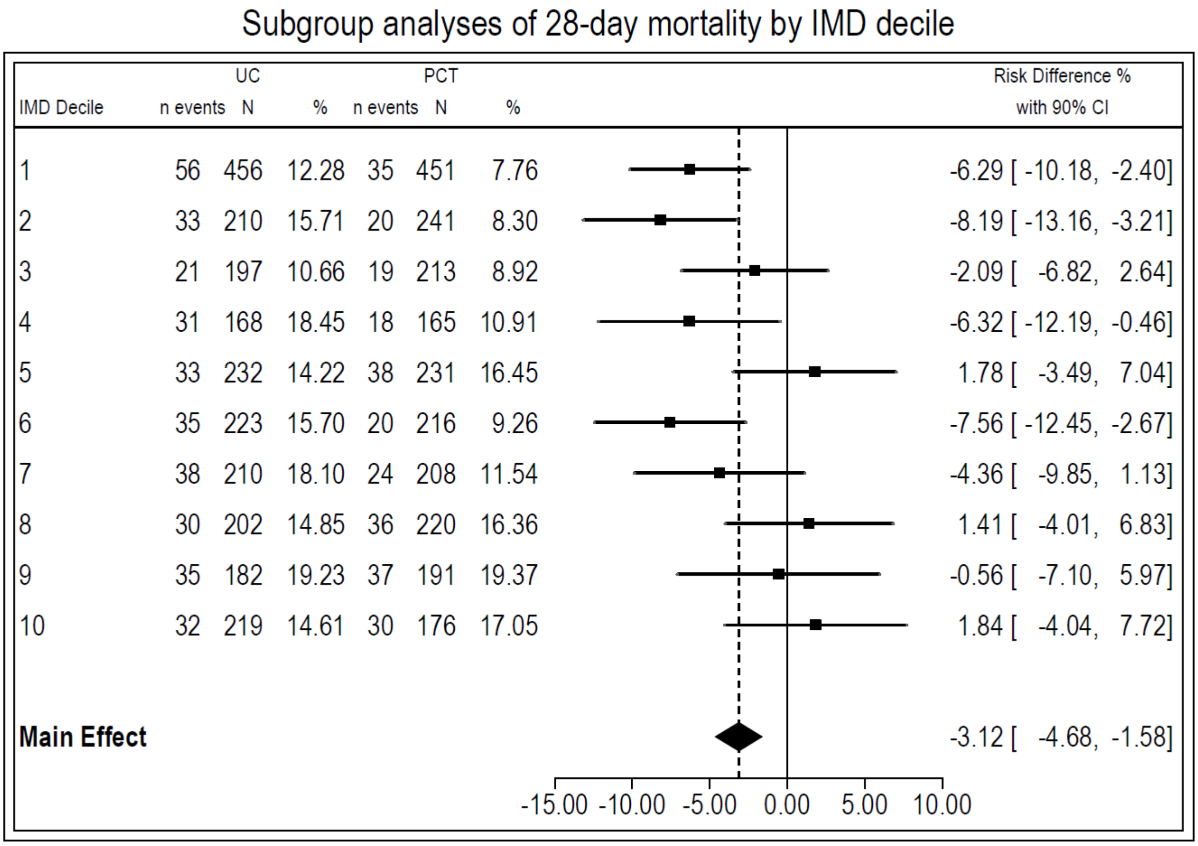

Figure s5 Histogram of days on any antibiotics over first 28 days (zero days included)

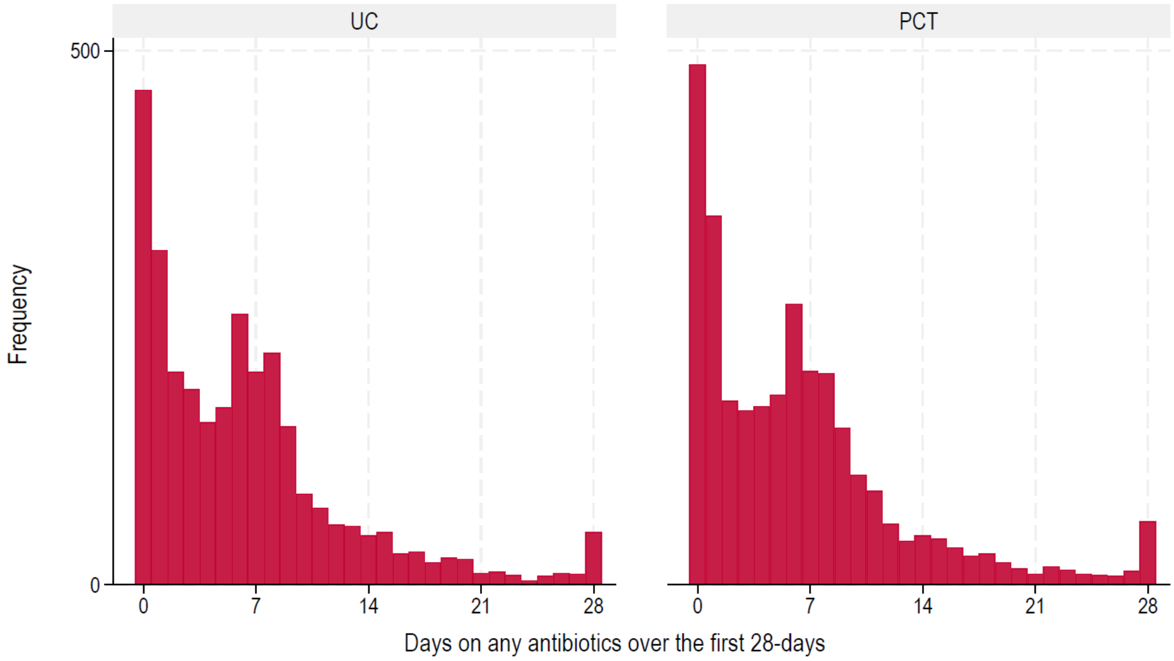

Figure s6 Histogram of days on IV antibiotics over first 28 days (zero days included)

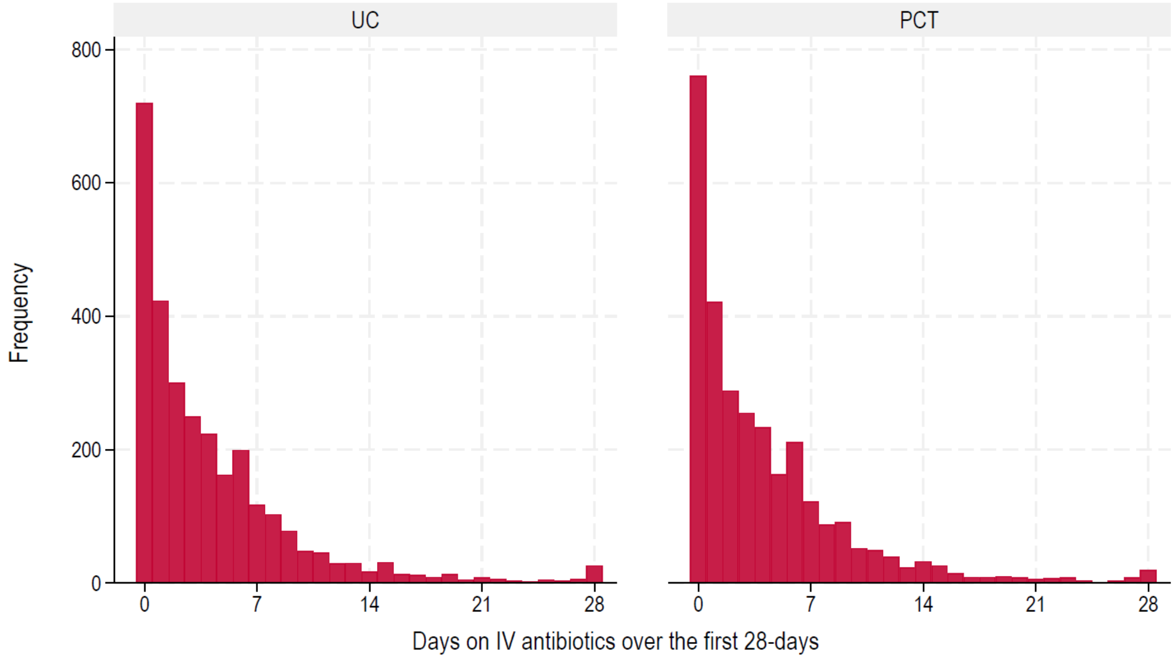

Figure s7 Histogram of days on broad spectrum (Watch/Reserve) antibiotics over first 28 days (zero days included)

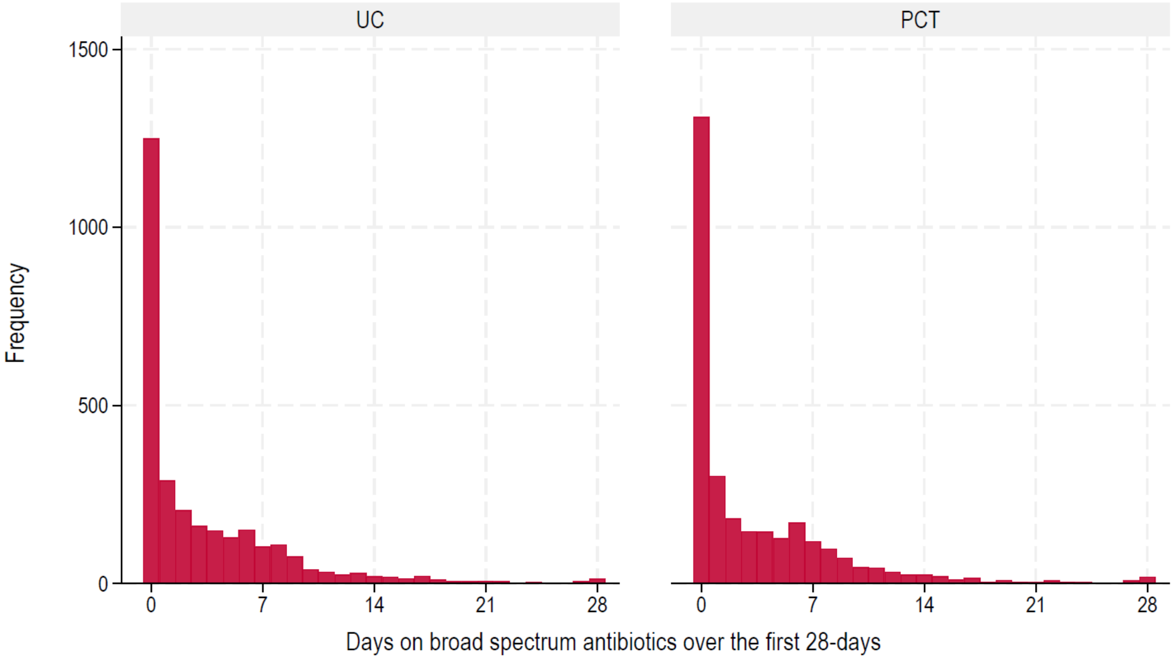

Table s17 Adverse Events by Arm

Total AEs	PCT	Usual care
0	2976/3042 (97.8%)	2911/2968 (98.1%)
1	53/3042 (1.7%)	46/2968 (1.6%)
2	9/3042 (0.3%)	5/2968 (0.2%)
3	3/3042 (0.1%)	3/2968 (0.1%)
4	1/3042 (0.0%)	1/2968 (0.0%)
6	0/3042 (0.0%)	1/2968 (0.0%)
21	0/3042 (0.0%)	1/2968 (0.0%)

Table s18 Serious Adverse Events by Arm

Total SAEs	PCT	Usual care
0	3041/3042 (99.9%)	2968/2968 (100%)
1	1/3042 (<0.01%)	0/2968 (0%)

Table s19 Participant timeline/pathway (primary analysis population)

Time between	PCT		Usual Care	
	**N**	**Median [IQR]**	**N**	**Median [IQR]**
**ED admission and triage assessment (minutes)**	2726	19 [7, 46]	2712	16 [6, 39]
**Triage assessment and randomisation (minutes)**	2726	19 [3, 41]	2712	20 [6, 47]
**Triage assessment and clinical risk assessment (minutes)**	2429	46 [11, 106]	2425	49 [15, 113]
**Clinical risk assessment and abx prescribed (minutes)**	1593	9 [-10, 49]	1584	9 [-12, 45]
**Abx prescribed and administered (minutes)**	1654	27 [13, 55]	1644	28 [15, 50]
**Clinical risk assessment and senior review (hours and minutes)**	2223	3h 12m [1h 33m, 5h 47m]	2206	3h 4m [1h 26m, 5h 53m]

Table s20 Participant timeline/pathway (all participants)

Time between	PCT		Usual Care	
	**N**	**Median [IQR]**	**N**	**Median [IQR]**
**ED admission and triage assessment (minutes)**	3063	19 [7, 44]	3008	17 [6, 39]
**Triage assessment and randomisation (minutes)**	3063	18 [3, 40]	3008	20 [6, 46]
**Triage assessment and clinical risk assessment (minutes)**	2737	44 [10, 103]	2684	46 [13, 109]
**Clinical risk assessment and abx prescribed (minutes)**	1844	9 [-10, 46]	1798	9 [-11, 45]
**Abx prescribed and administered (minutes)**	1920	28 [13, 54]	1869	28 [14, 50]
**Clinical risk assessment and senior review (hours and minutes)**	2511	3h 14m [1h 35m, 5h 52m]	2451	3h 5m [1h 27m, 5h 50m]

Table s21 Type of antibiotic received between 0-12 hours from triage assessment (primary analysis population)

NEWS2	Arm	N	No Abx within 12 hours	Oral	IV narrow	IV broad	Missing (missing all routes or start times)
≤4	UC	1051	386 (36.7%)	68 (6.5%)	284 (27.0%)	281 (26.7%)	32 (3.0%)
	PCT	1036	392 (37.8%)	69 (6.7%)	277 (26.7%)	273 (26.4%)	25 (2.4%)
5-6	UC	719	180 (25.0%)	59 (8.2%)	233 (32.4%)	227 (31.6%)	20 (2.8%)
	PCT	737	148 (20.1%)	79 (10.7%)	222 (30.1%)	272 (36.9%)	16 (2.2%)
≥7	UC	945	126 (13.3%)	50 (5.3%)	288 (30.5%)	476 (50.4%)	5 (0.5%)
	PCT	965	158 (16.4%)	61 (6.3%)	278 (28.8%)	451 (46.7%)	17 (1.7%)

Table s22 Type of antibiotic received between 0-12 hours from triage assessment (all participants)

NEWS2	Arm	N	No Abx within 12 hours	Oral	IV narrow	IV broad	Missing (missing all routes or start times)
≤4	UC	1145	409 (35.7%)	70 (6.1%)	297 (25.9%)	285 (24.9%)	84 (7.3%)
	PCT	1148	424 (36.9%)	74 (6.5%)	287 (25.0%)	279 (24.3%)	84 (7.3%)
5-6	UC	805	197 (24.5%)	64 (8.0%)	246 (30.6%)	233 (28.9%)	65 (8.1%)
	PCT	834	170 (20.4%)	83 (10.0%)	232 (27.8%)	277 (33.2%)	72 (8.6%)
≥7	UC	1077	163 (15.1%)	52 (4.8%)	293 (27.2%)	485 (45.0%)	84 (7.8%)
	PCT	1110	200 (18.0%)	65 (5.9%)	288 (26.0%)	466 (42.0%)	91 (8.2%)

Table s23 Top ten antibiotics of those initiated within 12-hours from triage

	PCT	UC
Amoxicillin/clavulanic Acid (IV)	670/3803 (17.6%)	659/3756 (17.6%)
Piperacillin/tazobactam	517/3803 (13.6%)	550/3756 (14.6%)
Clarithromycin	454/3803 (11.9%)	434/3756 (11.6%)
Gentamicin	404/3803 (10.6%)	418/3756 (11.1%)
Amoxicillin iv	292/3803 (7.7%)	286/3756 (7.6%)
Doxycycline	242/3803 (6.4%)	211/3756 (5.6%)
Metronidazole (IV)	163/3803 (4.3%)	173/3756 (4.6%)
Teicoplanin	137/3803 (3.6%)	137/3756 (3.7%)
Benzylpenicillin	111/3803 (2.9%)	81/3756 (2.2%)
Flucloxacillin	106/3803 (2.8%)	126/3756 (3.4%)
Other	705/3803 (18.5%)	681/3756 (18.1%)
Missing*	2/3803 (0.1%)	0/3756 (0.0%)

One entry per antibiotic, may be multiple entries per participant. *Start time within 12-hours but missing type of antibiotic.

Table s24 28-day mortality by type of antibiotic received between 0-12 hours from triage assessment (all participants)

NEWS 2	Arm	N	No abx within 12-hours	Oral (only)	IV narrow	IV broad	Missing (missing all routes or start times)
≤4	UC	1145	18/409 (4.4%)	4/70 (5.7%)	23/297 (7.7%)	20/285 (7.0%)	7/84 (8.3%)
	PCT	1148	19/424 (4.5%)	2/74 (2.7%)	15/287 (5.2%)	25/279 (9.0%)	3/84 (3.6%)
5-6	UC	805	28/197 (14.2%)	4/64 (6.3%)	33/246 (13.4%)	42/233 (18.0%)	8/65 (12.3%)
	PCT	834	12/170 (7.1%)	4/83 (4.8%)	24/232 (10.3%)	41/277 (14.8%)	13/72 (18.1%)
≥7	UC	1077	53/163 (32.5%)	7/52 (13.5%)	81/293 (27.7%)	147/485 (30.3%)	28/84 (33.3%)
	PCT	1110	62/200 (31.0%)	5/65 (7.7%)	56/288 (19.4%)	131/466 (28.1%)	17/91 (18.7%)

Figure s8 Change in A) Proportion of Antibiotic at 12 hours B) 28 Day Mortality by Antibiotic Route at 12 hours and Baseline NEWS2

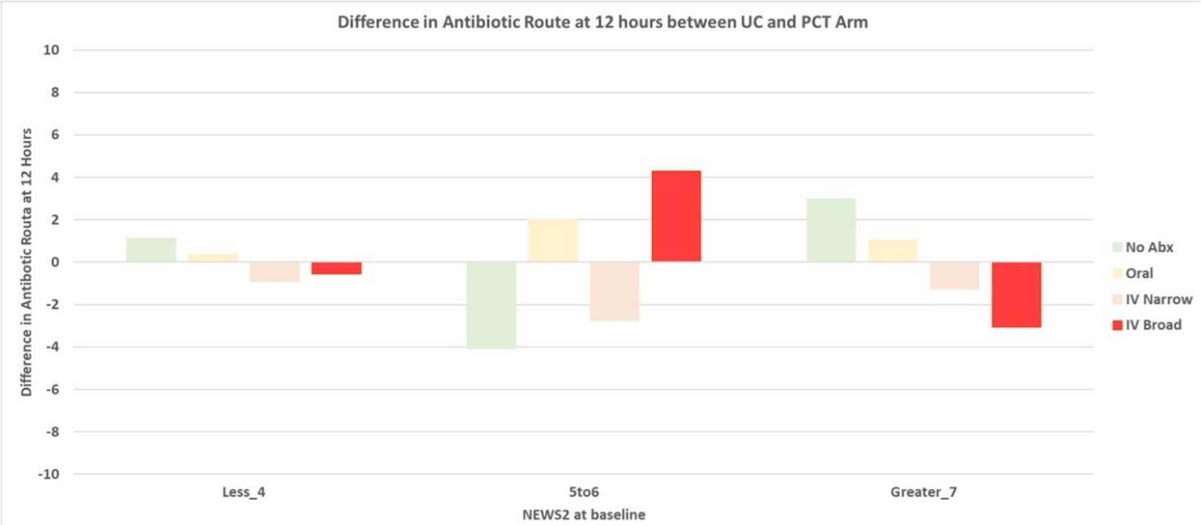

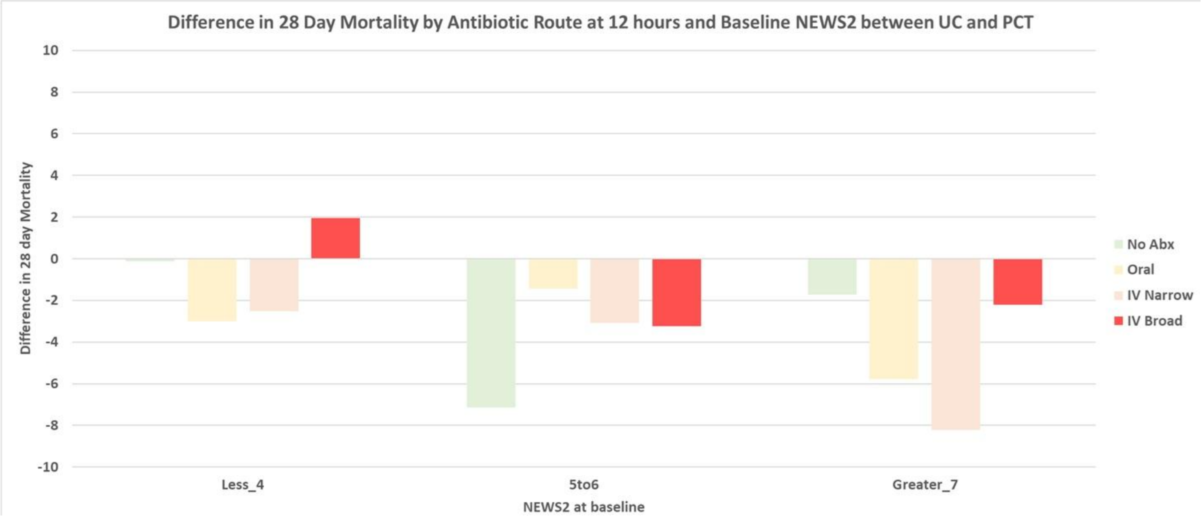

Table s25 Oxygen therapy (primary analysis population)

Outcome	PCT	UC	Analysis	n	OR [95% CI]	p-value
Required supplemental Oxygen	1353/2658 (50.9%)	1332/2624 (50.8%)	Unadjusted	5282	0.99 [0.88 to 1.11]	0.86
	1335/2619 (51.0%)	1317/2595 (50.8%)	Adjusted	5214	1.02 [0.91 to 1.16]	0.71

Table s26 Oxygen therapy (secondary outcome analysis population)

Outcome	PCT	UC	Analysis	n	OR [95% CI]	p-value
Required supplemental Oxygen	1486/2945 (50.5%)	1447/2860 (50.6%)	Unadjusted	5805	0.98 [0.87 to 1.10]	0.71
	1468/2904 (50.6%)	1432/2831 (50.6%)	Adjusted	5735	1.01 [0.90 to 1.13]	0.90

Table s27 Grade of most senior clinician conducting assessment (primary analysis population)

**Grade**	**PCT**	**Usual care**
Resident Doctor (below ST3/Registrar or equivalent)	860/2738 (31.4%)	857/2715 (31.6%)
Senior Clinical Decision Maker (ST3/Registrar or equivalent, Non-Consultant)	950/2738 (34.7%)	949/2715 (35.0%)
Senior Clinical Decision Maker (Consultant)	433/2738 (15.8%)	413/2715 (15.2%)
Advanced Clinical Practitioner (non-medical)	289/2738 (10.6%)	291/2715 (10.7%)
Other	25/2738 (0.9%)	27/2715 (1.0%)
Missing	181/2738 (6.6%)	178/2715 (6.6%)

Table s28 Subgroup analysis for the grade of the most senior clinician conducting assessment (primary analysis population)

**Outcome**	**n**	**LRT χ^2 (df)**	**p-value**
**28-day mortality (non-inferiority)** [(a)]	5094	4.67 (4)	0.32
**IV antimicrobial initiation at 3 hours (superiority)**[(a)]	5094	1.43 (4)	0.84

Analysis method: (a) multilevel logistic regression. Interaction tests by model comparison.
Covariates in all models: baseline NEWS2.

Figure s9 Change in Diagnosis Category from Initial Assessment to Final Diagnosis by Arm

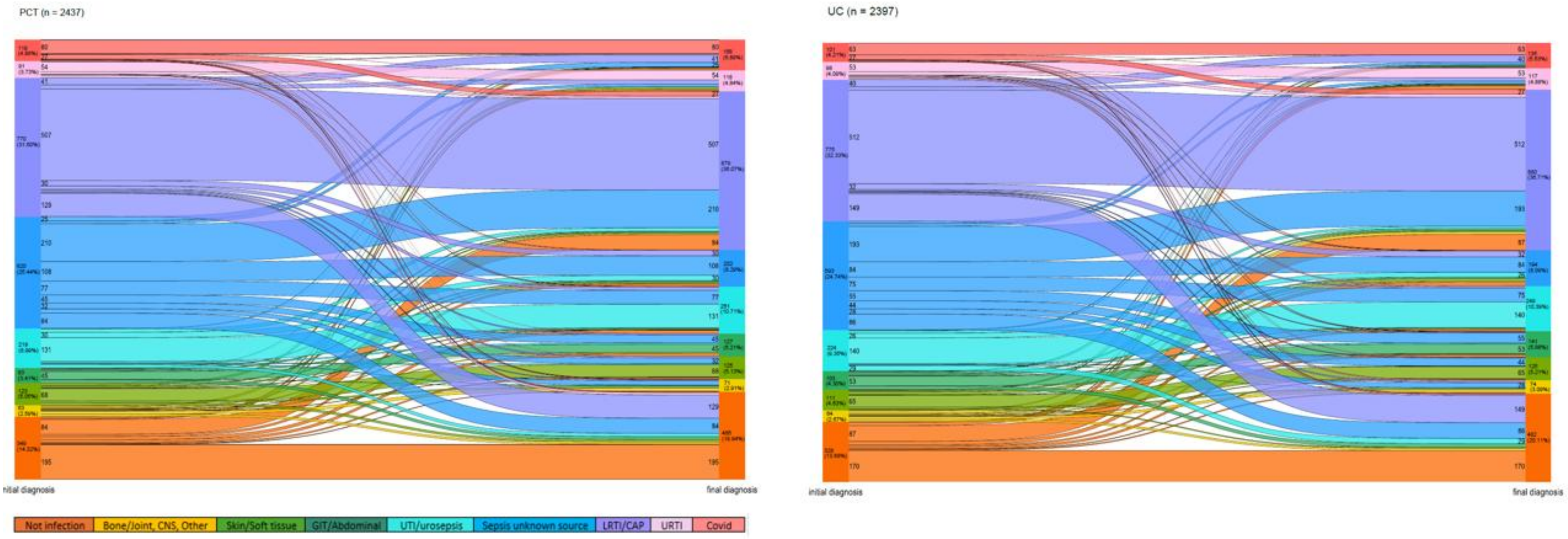

Table s29 Initial and final diagnosis by trial arm (primary analysis population)

	Initial Diagnosis		Discharge Diagnosis	
	PCT	Usual care	PCT	Usual care
LRTI/CAP	799/2738 (29·2%)	810/2715 (29·8%)	961/2738 (35·1%)	960/2715 (35·4%)
Sepsis (unknown source)	639/2738 (23·3%)	626/2715 (23·1%)	223/2738 (8·1%)	212/2715 (7·8%)
Urinary tract infection/urosepsis	228/2738 (8·3%)	230/2715 (8·5%)	276/2738 (10·1%)	276/2715 (10·2%)
Skin/soft tissue infection	125/2738 (4·56%)	114/2715 (4·2%)	134/2738 (4·9%)	136/2715 (5·0%)
COVID	122/2738 (4·5%)	103/2715 (3·8%)	189/2738 (6·9%)	151/2715 (5·6%)
URTI	93/2738 (3·4%)	100/2715 (3·7%)	120/2738 (4·4%)	121/2715 (4·5%)
GIT/Abdominal infection	84/2738 (3·1%)	106/2715 (3·9%)	139/2738 (5·1%)	146/2715 (5·4%)
Other infections	42/2738 (1·5%)	42/2715 (1·6%)	40/2738 (1·5%)	46/2715 (1·7%)
CNS infection	13/2738 (0·5%)	12/2715 (0·4%)	8/2738 (0·3%)	5/2715 (0·2%)
Bone/Joint/Muscle infection	10/2738 (0·4%)	13/2715 (0·5%)	29/2738 (1·1%)	30/2715 (1·1%)
Not infection	366/2738 (13·4%)	342/2715 (12·6%)	528/2738 (19·3%)	521/2715 (19·2%)
Missing	217/2738 (7·9%)	217/2715 (8·0%)	91/2738 (3·3%)	111/2715 (4·1%)

Table s30 Blood cultures taken within 24 hours of admission

	**PCT**	**UC**
Participants	3092	3027
No blood culture taken	1314/3092 (42.5%)	1310/3027 (43.3%)
Blood Culture – No Growth	1560/3092 (50.5%)	1499/3027 (49.5%)
Blood Culture - Positive	207/3092 (6.7%)	197/3027 (6.5%)
Date of triage or blood culture missing	11/3092 (0.3%)	21/3027 (0.7%)

Table s31 28-day mortality by final diagnosis (primary analysis population) (where final diagnosis was a single non-infectious diagnosis)

	NEWS2	PCT	UC
Infectious	≤4	36/788 (4.6%)	35/781 (4.5%)
	5-6	46/573 (8.0%)	68/552 (12.3%)
	≥7	156/758 (20.6%)	183/750 (24.4%)
Non-infectious (only)	≤4	14/228 (6.1%)	14/240 (5.8%)
	5-6	16/140 (11.4%)	19/141 (13.5%)
	≥7	39/160 (24.4%)	45/140 (32.1%)
Missing		65/91 (71.4%)	86/111 (77.5%)

Table s32 28-day mortality by final diagnosis (primary analysis population) (where final diagnosis had more than one diagnosis listed and included a non-infectious diagnosis)

	NEWS2	PCT	UC
Infectious	≤4	18/313 (5.8%)	21/326 (6.4%)
	5-6	28/211 (13.3%)	36/215 (16.7%)
	≥7	62/288 (21.5%)	80/235 (34.0%)
Non-infectious (considered)	≤4	32/703 (4.6%)	28/695 (4.0%)
	5-6	34/502 (6.8%)	51/478 (10.7%)
	≥7	133/630 (21.1%)	148/655 (22.6%)
Missing		65/91 (71.4%)	86/111 (77.5%)

Table s33 90-day mortality by final diagnosis (all participants) (where final diagnosis was a single non-infectious diagnosis)

	NEWS2	PCT	UC
Infectious	≤4	70/857 (8.2%)	84/834 (10.1%)
	5-6	85/627 (13.6%)	113/593 (19.1%)
	≥7	246/849 (29.0%)	264/814 (32.4%)
Non-infectious (only)	≤4	27/231 (11.7%)	31/249 (12.5%)
	5-6	27/142 (19.0%)	27/147 (18.4%)
	≥7	51/172 (29.7%)	62/149 (41.6%)
Missing		92/112 (82.1%)	112/128 (87.5%)

Table s34 90-day mortality by final diagnosis (all participants) (where final diagnosis had more than one diagnosis listed and included a non-infectious diagnosis)

	NEWS2	PCT	UC
Infectious	≤4	39/329 (11.9%)	52/342 (15.2%)
	5-6	50/221 (22.6%)	56/228 (24.6%)
	≥7	95/314 (30.3%)	114/251 (45.4%)
Non-infectious (considered)	≤4	58/759 (7.6%)	63/741 (8.5%)
	5-6	62/548 (11.3%)	84/512 (16.4%)
	≥7	202/707 (28.6%)	212/712 (29.8%)
Missing		92/112 (82.1%)	112/128 (87.5%)

Table s35 Site level heterogeneity by estimating the variance of the random intercept for site in the co-primary outcome models (primary analysis population)

Outcome	Analysis	Site level variance (SE)	CI
28-day mortality	Unadjusted	0.062 (0.033)	90% CI: 0.026, 0.149
	Adjusted	0.074 (0.037)	90% CI: 0.032, 0.170
IV antimicrobial initiation at 3 hours	Unadjusted	0.220 (0.087)	95% CI: 0.102, 0.477
	Adjusted	0.222 (0.088)	95% CI: 0.102, 0.481

IV = intravenous, SE = standard error, CI = confidence interval. Analysis method: two-level logistic regression models. Covariates in unadjusted modes: baseline NEWS2. Covariates in adjusted models: baseline NEWS2, age, gender, Charlson Comorbidity Index.

4. Supplementary Material References

1. legislation.gov.uk. Mental Capacity Act 2005: Section 2. 01 April 2015 2005. https://www.legislation.gov.uk/ukpga/2005/9 (accessed 14th Jan 2025 2025).
2. Euden J, Thomas-Jones E, Aston S, et al. PROcalcitonin and NEWS2 evaluation for Timely identification of sepsis and Optimal use of antibiotics in the emergency department (PRONTO): protocol for a multicentre, open-label, randomised controlled trial. *BMJ Open* 2022; **12**(6): e063424.
3. O'Brien PC, Fleming TR. A multiple testing procedure for clinical trials. *Biometrics* 1979; **35**(3): 549-56.
4. DeMets DL, Lan KK. Interim analysis: the alpha spending function approach. *Stat Med* 1994; **13**(13-14): 1341-52; discussion 53-6.

Appendix A Participant Informed Consent Form

PROcalcitonin and NEWS2 evaluation for Timely identification of sepsis and Optimal use of antibiotics in the Emergency Department

Site ID

PARTICIPANT CONSENT FORM

Chief Investigator: Professor Neil French, University of Liverpool

(Please **initial** each box and sign in full at the bottom of the page)

1. I confirm that I have read and understood the Patient Information Sheet (version 2.1, dated 22.12.2022) for the PRONTO trial. I have had the opportunity to consider the information, ask questions and have had these answered satisfactorily. ☐

2. I understand that I have already entered the trial but do not have to continue to take part. I understand that I can agree to take part in different parts of the trial and will indicate my choice below. I understand *that I am free to withdraw my consent at any time, without giving any reason, without my normal medical care or legal rights being affected*. ☐

3. I understand the trial is randomised and no one has picked which treatment I received. I understand that I was randomised to have either an additional procalcitonin test or standard care. If I was allocated to the treatment arm of the trial, procalcitonin levels in my blood were tested as part of routine blood tests, or via an additional finger prick/collection of very small sample of blood from my vein in the absence of routine blood collection. I consent to the data generated from the procalcitonin test to be used for the purposes of this trial. ☐

4. I understand that information collected during the trial can be used by the study team to look at treatment of sepsis in patients presenting to the emergency department. ☐

5. I understand that relevant information about me will be collected from my medical records and other health and social care-related records and looked at by the research team and responsible practitioners during the study. This information may also be reviewed by regulatory bodies and NHS Trusts where it is relevant to conduct of this study. I give permission for these individuals to have access to my records. ☐

6. I understand that information collected about me (including name and address) will be held at the Centre for Trials Research, Cardiff University according to the 2018 General Data Protection Regulation (GDPR) (EU 2016/679). I understand that this information will be kept securely, and viewed and shared with the PRONTO research team and other approved organisations in accordance with my choices below and that no personal identifiable information will be used in the study report or publications. ☐

7. I agree to continue to take part in this trial. ☐

Please select which aspects you agree to take part in:

8. I agree that information collected as part of the trial and data from my medical records up to this point can be used in the trial. ☐

9. I agree that data from my records can be collected for the 90 days of the study. ☐

10. I agree to be contacted at day 28 and day 90 to ask about my health, wellbeing and any further medical treatment I may have received. I give my consent for a member of the reserch team to contact me by the following methods to complete these surveys: ☐

    Telephone ☐

    Email ☐

    Post ☐

11. I agree that relevant health and social care data about me which is held by other health, government or research organisations, such as NHS Digital or CIPHA, can be shared with the PRONTO research team where appropriate approvals are in place (future data linkage studies). I understand that this could include data covering the year prior to my enrolment in PRONTO, for the duration of PRONTO trial and up to 3 years after the completion of the study. In order for this to occur personal identifiers (such as my NHS number, DOB, Postcode for example) will need to be shared securely with these organisations. ☐

12. I agree that anonymised information collected as part of this trial can be used in other research studies (including with other researchers) which have been approved by appropriate NHS (or equivalent) regulatory bodies. ☐

13. I agree to be invited to an interview about my health experiences, my views on treatment, and what it was like to take part in the PRONTO trial. ☐

Name of Participant: ________________________ Signed:____________ Date:___/___/___

Name of Person taking Consent: ______________________ Signed:____________ Date:___/___/___

Appendix B Statistical Analysis Plan

Short title:	ISAP / SAP Template

**Statistical Analysis Plan for PRONTO: PROcalcitonin and NEWS2 evaluation for Timely identification of sepsis and Optimal use of antibiotics in the emergency department**			
ISRCTN No:	54006056	Version Number:	2.0 (03/12/24)

**<u>Final Plan</u>**
Based on protocol version: 3.2 (25/03/24)

**SAP Revision History**				
Protocol version	Updated SAP version	Section number changed	Description and reason for change	Date changed
3.2	2.0	SAP deviation log	Added in post hoc exploratory analyses	03/12/24

Short title:	ISAP / SAP Template

ROLES AND RESPONSIBILITIES

**Trial Statistician: Jennifer Condie**			
**Role: Research Assistant in Statistics**			
Date:	14/01/25	Signature:	
**Senior Statistician: Dr Philip Pallmann**			
**Role: Principal Research Fellow in Statistics**			
Date:	15/01/25	Signature:	
**Chief Investigators: Professor Neil French**			
**Role: Co-Chief Investigators**			
Date:	15/01/25	Signature:	
**Chief Investigators: Dr Stacy Todd**			
**Role: Co-Chief Investigators**			
Date:	14/01/2025	Signature:	
**Other non-signatory contributor to the SAP: N/A**			
**Role: N/A**			

TABLE OF CONTENTS

1. INTRODUCTION 6
2. BACKGROUND 6
2.1 RATIONALE AND RESEARCH QUESTION 6
2.2 OBJECTIVES 6
3. STUDY MATERIALS 7
3.1 TRIAL DESIGN 7
3.2 RANDOMISATION 9
3.3 SAMPLE SIZE 9
3.4 FRAMEWORK 10
3.5 INTERIM ANALYSES 10
3.5.1 PLANNED SAMPLE SIZE ADJUSTMENT 11
3.5.2 STOPPING RULES 11
3.6 TIMING OF FINAL ANALYSIS 11
3.7 TIMING OF OUTCOME ASSESSMENT 12
4. STATISTICAL PRINCIPLES 13
5. STUDY POPULATION 16
5.1 SCREENING DATA 16
5.2 ELIGIBILITY 16
5.3 RECRUITMENT 16
5.4 WITHDRAWAL/FOLLOW UP 17
5.4.1 LEVEL OF WITHDRAWAL 17

5.4.2 TIMING OF WITHDRAWAL 17
5.4.3 REASONS FOR WITHDRAWAL 18
5.4.4 PRESENTATION OF WITHDRAWAL/LOSS TO FOLLOW-UP 18
5.5 BASELINE PARTICPANT CHARACTERISTICS 18
5.5.1 LIST OF BASELINE DATA 18
5.5.2 DESCRIPTIVE STATISTICS 18
6. ANALYSIS 18
6.1 OUTCOME DEFINITIONS 18
6.1.1 PRIMARY OUTCOME(S) 18
6.1.2 TIMING, UNITS AND DERIVATION OF PRIMARY 19
6.1.3 LIST OF SECONDARY OUTCOMES 19
6.1.4 ORDER OF TESTING 19
6.1.5 TIMING, UNITS AND DERIVATION OF SECONDARIES 20
6.2 ANALYSIS METHODS 20
6.2.1 LIST OF METHODS AND PRESENTATION 20
6.2.2 COVARIATE ADJUSTMENT 26
6.2.3 ASSUMPTION CHECKING 26
6.2.4 ALTERNATIVE METHODS IF DISTRIBTIONAL ASSUMPTIONS NOT MET 26
6.2.5 SENSITIVITY ANALYSES 26
6.2.6 SUBGROUP ANALYSES 27
6.3 MISSING DATA 28
6.4 ADDITIONAL ANALYSES 29
6.5 HARMS 29
6.6 STATISTICAL SOFTWARE 29
7. REFERENCES 30
7.1 NON-STANDARD STATISTICAL METHODS 30
7.2 DATA MANAGEMENT PLAN 31
7.3 TRIAL MASTER FILE AND STATISTICAL MASTER FILE 31
7.4 OTHER SOPS OR GUIDANCE DOCUMENTS 31
8. APPENDICES 34

8.1 Dummy tables.. Error! Bookmark not defined.

1. INTRODUCTION

This statistical analysis plan (SAP) provides guidelines for the final presentation and analysis for the PROcalcitonin and NEWS2 evaluation for Timely identification of sepsis and Optimal use of antibiotics in the emergency department (PRONTO) trial. This plan, along with all other documents relating to the analysis of this trial, will be stored in the Statistical Analysis Master File electronically within the Trial Master File.

Any deviations from the SAP will be recorded and justified in the SAP deviation log (at the end of this document) and the final report. The analysis will be conducted by an appropriately qualified statistician, who will ensure data integrity by adhering to the guidelines set out in the CTR's (Centre for Trials Research) SOPs (Standard Operating Procedures). This SAP will be reviewed by the senior trial statistician (STS) and approved by the Trial Management Group (TMG) before being signed off by the author (Jennifer Condie), STS (Dr Philip Pallmann), and the Chief Investigators (Professor Neil French and Dr Stacy Todd). A copy of the SAP will be sent to the Trial Steering Committee (TSC) and Independent Data Monitoring Committee (IDMC) statisticians for review and amended as appropriate. This SAP includes the quantitative aspects of the analysis; health economics and qualitative analysis plans will be provided separately.

2. BACKGROUND

2.1 RATIONALE AND RESEARCH QUESTION

Full trial details are provided in the PRONTO trial protocol and the protocol paper (Euden et al., 2022).

Sepsis is a common, potentially life-threatening complication of infection. The optimal treatment for sepsis includes early recognition, prompt antibiotics and fluids into a vein (intravenous/IV). Currently, clinicians assess severity in patients in the Emergency Department (ED) with the National Early Warning Score (NEWS2). It is not specific and tends to over-diagnose sepsis leading to over-prescribing of antibiotics and promoting antimicrobial resistance. Adults with suspected sepsis fall into one of three categories: a) those looking ill needing urgent IV antibiotics and fluids within 1 hour, b) those who are unwell, but will not come to harm if IV antibiotics are not administered within 1 hour, allowing time for further assessment prior to starting antibiotics within 3 hours, c) those not critically unwell who may or may not need IV antibiotics. Procalcitonin (PCT), a blood test not widely used in the NHS, helps to identify bacterial infection. The National Institute for Health and Care Excellence (NICE) recommended further research on PCT testing in EDs for guiding antibiotic use in people with suspected sepsis.

2.2 OBJECTIVES

Primary objective: To determine whether the addition of PCT measurement to NEWS2 scoring can lead to a reduction in IV antibiotic initiation in ED patients managed as suspected sepsis, with at least no increase in 28-day mortality compared to NEWS2 scoring alone (in conjunction with local standard care pathways).

Secondary objectives: To determine if the use of PCT and NEWS2 in the assessment of suspected sepsis is:

i. cost-effective
ii. feasible
iii. acceptable to patients and their families

3. STUDY MATERIALS

3.1 TRIAL DESIGN

Parallel, two-arm, open-label, individually randomised controlled trial with two co-primary endpoints, an internal pilot phase, and group-sequential stopping rules for effectiveness. Participants are randomised in a ratio of 1:1 to PCT-guided assessment added to NEWS2 and local standard care, or NEWS2 and local standard care alone. The participant flow diagram (Figure 1) provides further details.

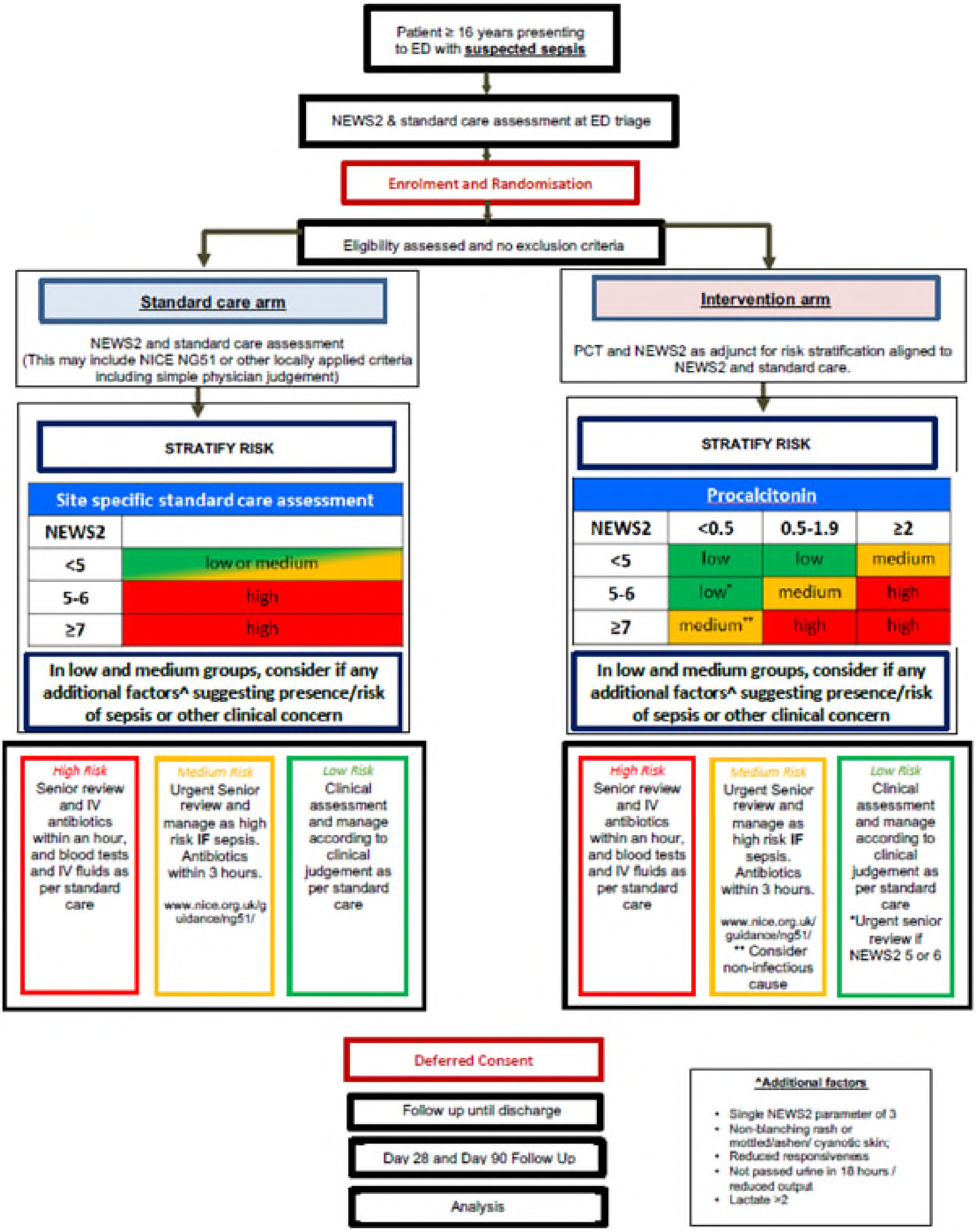

Figure 1: PRONTO trial participant flow diagram.

3.2 RANDOMISATION

Individual patients with suspected sepsis were randomised in a 1:1 ratio to either standard clinical management based on NEWS2 (control), or standard clinical management based on NEWS2 plus PCT-guided assessment (intervention). We used minimisation with NEWS2 score and site as stratification factors and added a random element to reduce the risk of subversion. This was implemented in a secure 24-hour web-based randomisation programme controlled centrally by the Centre for Trials Research.

The first patient was allocated at random. For each subsequent patient the software calculated the covariate sums as explained in Altman & Bland (2005) and determined which arm the patient should be allocated to in order to minimise the covariate imbalance. The patient was then randomised with an 80% chance of being allocated to the arm that minimised the imbalance (and a 20% chance of being allocated to the other arm). If neither arm minimised the imbalance because the covariate sums were equal for both arms (i.e., a state of perfect balance) the patient was allocated at random.

3.3 SAMPLE SIZE

The sample size calculation was based on two co-primary outcomes:

1. 28-day mortality, for which we want to show non-inferiority of the PCT-guided assessment as compared to current standard practice, using an absolute 2.5% non-inferiority margin. Assuming 28-day mortality of 15% in patients managed as suspected sepsis treated in the ED, any increase in 28-day mortality from 15% to not more than 17.5% would be considered non-inferior. For 90% power and one-sided 5% significance level, the sample size required is 7002, assuming there is no difference in 28-day mortality between arms. Our patient focus group were also consulted on the 2.5% non-inferiority margin and felt that this was acceptable if there were mechanisms to monitor trial outcomes, and if this was what was needed to provide a sample size which would ensure the trial could be completed as well as answer the research question.

2. Initiation of antibiotic treatment, for which we want to show superiority. Currently, around 90% of patients managed as suspected sepsis receive antibiotics (Royal Liverpool and Broadgreen University Hospitals NHS Trust, unpublished data). Reducing this by 10 percentage points to 80% would be seen as a success. To detect such an effect with 90% power and a two-sided 5% significance level, the sample size required is 532, which is substantially lower than what is needed for the non-inferiority endpoint. With 7002 patients, we would be able to detect effects as small as a reduction from 90% to 87.6% prescriptions with 90% power.

Accounting for 5% dropout, a fixed-sample design would need a total sample size of 7372.

We planned to conduct one interim analysis (after 50% of patients provided data) with options to stop the trial early using group-sequential boundaries based on O'Brien-Fleming type alpha spending (O'Brien & Fleming, 1979, DeMets & Lan, 1994). We used a hierarchical approach (Figure 2) to recommend stopping for effectiveness if:

- the PCT-guided assessment was non-inferior in terms of 28-day mortality and superior in terms of initiation of antibiotics, or
- the PCT-guided assessment was superior in terms of 28-day mortality (i.e., a significant reduction to less than 15%).

The group-sequential design increased the total maximum sample size (in case the study was not stopped after the interim analysis) by just over 4% to 7676 (inflated for 5% dropout). The sample sizes were calculated using PROC POWER and PROC SEQDESIGN in SAS version 9.4 (SAS Institute Inc., Cary, NC, USA).

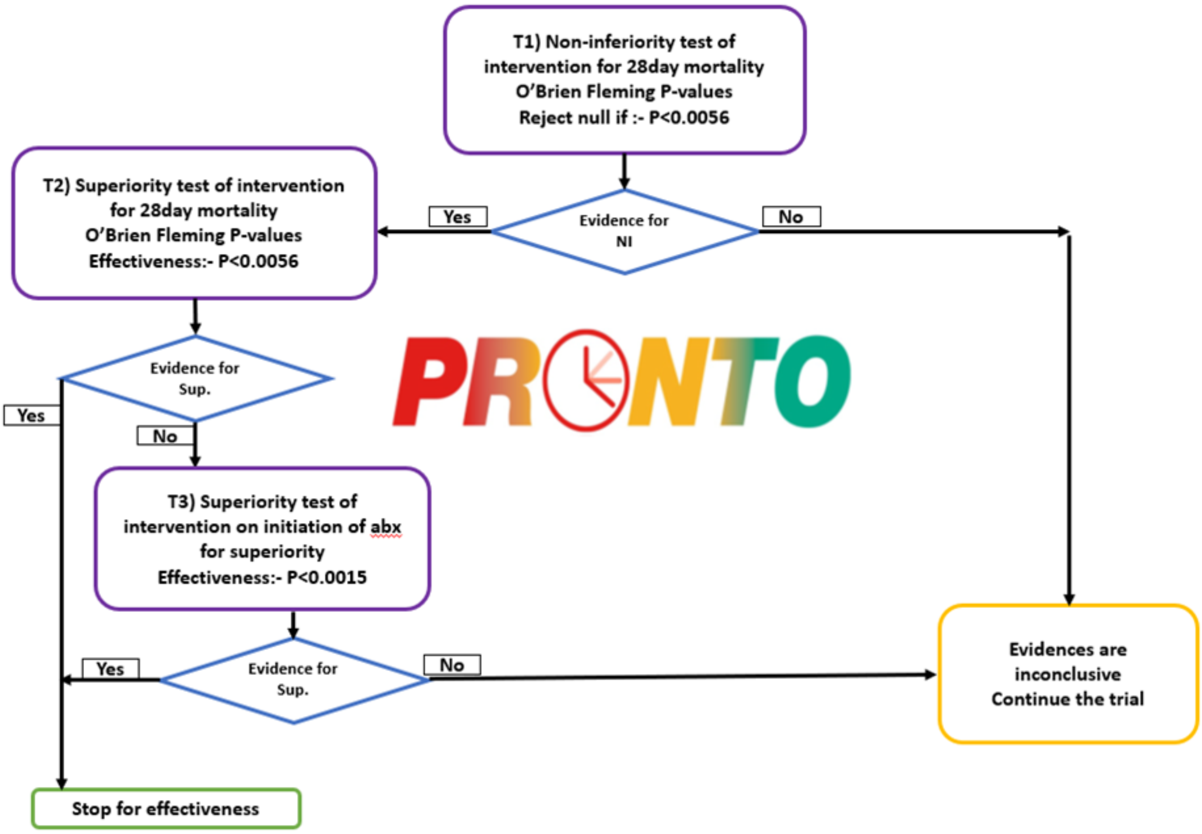

Figure 2: Overview of decision-making at the interim analysis of the co-primary outcomes.

3.4 FRAMEWORK

The effect of 28-day mortality will be investigated for non-inferiority of the PCT-guided assessment as compared to current standard practice, using an absolute 2.5% non-inferiority margin. For measuring the effect of initiation of antibiotics treatment, we want to show superiority.

3.5 INTERIM ANALYSES

A planned interim analysis was conducted after 43% (3040/7002) of participants had been recruited and followed up for 28 days. A second, unplanned interim analysis was requested by the Independent Data Monitoring Committee (IDMC) and conducted after 57% (3973/7002) of participants had been recruited and followed up for 28 days.

A full description of the interim analysis strategy and stopping rules is provided in the interim SAP.

3.5.1 PLANNED SAMPLE SIZE ADJUSTMENT

Not applicable.

3.5.2 STOPPING RULES

The planned interim analysis cut-offs were fixed on 50% information, but the actual analysis used 43% (3040 patients). As described in the interim SAP, we have re-estimated the stopping boundaries accordingly (Table 1). The exact stopping criteria for each test (T1-T3, see Figure 2) and associated operating characteristics of the design are listed below:

Table 1: Modified boundaries for the interim analysis

		**Interim analysis***	**Final analysis**
**Test 1: non-inferiority test for 28-day mortality**<br><br>**Test 2: superiority test for 28-day mortality**	Information rate	0.430	1.000
	Effectiveness boundary (test statistic)	2.770	1.654
	One-sided local significance level	0.0028	0.0491
	Exit probability under the null<br>(i.e., cumulative **one-sided** alpha spent)	0.0028	0.0500
	Exit probability under the alternative (i.e., cumulative power)	0.198	0.900
**Test 3: superiority test for IV antibiotic initiation**	Information rate	0.430	1.000
	Effectiveness boundary (test statistic)	3.225	1.964
	Two-sided local significance level	0.0013	0.0496
	Exit probability under the null<br>(i.e., cumulative **two-sided** alpha spent)	0.0013	0.0500
	Exit probability under the alternative (i.e., cumulative power)	0.1362	>0.999
* We followed a conservative approach of adjusting the level of information to 43%, which is based on test 1 sample size estimation (used in the study).			

3.6 TIMING OF FINAL ANALYSIS

Data collection will be completed by November 2023. Data cleaning will take place between November 2023 and March 2024. Data analysis will take place between April and July 2024 and be published in the autumn of 2024.

3.7 TIMING OF OUTCOME ASSESSMENT

Outcome data will be recorded daily by the research nurse for all recruited participants (up to and including day 28, or until discharge). Patient reported outcome data (health-related quality of life and resource use questionnaires) will be recorded at day 28 and day 90, with the exception of those recruited within the last 3 months of the study (to maximise recruitment, we stopped collecting these questionnaires on the 29/04/22 so they have not been collected for all participants). Research nurses will review observation and medication charts, and medical notes for all recruited participants to collect the data described in Table 2 below:

Table 2: Outcome data collection

Outcome	Data source	Type of data	Frequency	By whom
**Antibiotic (Abx) initiation**	Observation (Obs) charts/medical notes/drug charts	Time of initiation, Abx type, dose, duration	Admission/daily	Research Nurse
**Abx use (IV and oral) in-patient**	Obs charts/medical notes/drug charts	Abx type, dose, duration	Daily	Research Nurse
**Abx use (IV and oral) post discharge up to 28 days**	Obs charts/medical notes/drug charts/patient report/GP record	Abx type, dose, duration	Up to 28 days	Research Nurse
**Adverse events**	Obs charts/medical notes	Date, type	Daily	Research Nurse
**Intensive care unit (ICU) usage**	Medical notes	Date, details of admission/discharge to ICU	Daily	Research Nurse
**COVID diagnosis**	Medical notes	Date, clinical or laboratory confirmed	Up to 28 days	Research Nurse
**Unscheduled readmissions**	Medical notes	ICU readmissions, readmissions post discharge	Daily	Research Nurse
**Mortality**	Medical notes	Date, description	Up to 90 days	Research Nurse
**Discharge**	Medical notes	Date, description	Up to 90 days	Research Nurse

**Serious adverse drug reactions (ADRs)**	Medical notes	ADR(s)	Daily	Research Nurse
**Health utility**	Patient reported	-	Day 28 and day 90	EQ-5D/5L, patient reported questionnaire, collected by telephone or by post
**Health-related quality of life (EQ-5D/5L)**	Patient reported	-	Day 28 and day 90	Patient reported, collected by telephone, or by post
**Resource use**	Patient reported	Direct medical costs and resource use	Day 28 and day 90	Patient reported, collected by telephone, or by post

4. STATISTICAL PRINCIPLES

4.1 LEVELS OF CONFIDENCE AND P-VALUES

The trial follows the group-sequential approach with one interim analysis; thus, p-values will be adjusted using the O'Brien-Fleming type alpha spending approach. For the hypothesis testing, any p-value less than the adjusted level of significance at the final analysis (Table 1 in Section 3.5.2) will be considered sufficient evidence to reject the hypothesis.

We will consider the 95% confidence limit (corresponding to a 5% significance level) as a benchmark for all analyses. We will estimate a two-sided 90% confidence interval (corresponding to a 95% one-sided confidence level) to assess the non-inferiority of the 28-day mortality outcome, and a two-sided 95% confidence interval to assess the superiority of the IV antibiotic initiation outcome. We will report both unadjusted and OBF adjusted confidence intervals for both co-primary outcomes.

We will also report bias-adjusted confidence intervals for group sequential designs, calculated using Jennison and Turnbull's repeated CI approach (Jennison & Turnbull, 1989).

Results will be presented in line with the CONSORT statement and its extensions for non-inferiority (Piaggio et al., 2012) and adaptive designs (Dimairo et al., 2020).

4.1.1 ADJUSTMENT FOR MULTIPLICITY

We have used the O'Brien-Fleming type alpha spending method to adjust the interim analysis and final analysis level of significance. Adjustment for multiplicity of endpoints is not required because they are co-primary, therefore the resulting test procedure is an intersection-union test (Offen et al., 2007).

4.2 ADHERENCE AND PROTOCOL DEVIATIONS

4.2.1 DEFINITION AND ASSESSMENT OF ADHERENCE

Adherence to the algorithm will be recorded on the CRF and will capture instances where the treating clinician overrules the algorithm if they feel it is appropriate to do so. The ultimate responsibility for clinical care of the patient lies with the treating clinician; therefore, the cut-off boundaries for initiation times of antibiotics are not mandatory but are recommended guidance to aid clinical decision-making. The trial aims to assess whether the use of PCT can improve decision-making about which patients receive antibiotics and in what time period. Deviations from the algorithm will not be recorded as protocol violations.

4.2.2 PRESENTATION OF ADHERENCE

Descriptive statistics on adherence will be presented in a table, overall and by trial arm. The proportion of non-adherence and reason for non-adherence will be reported overall and as group-wise relative frequencies and percentages.

4.2.3 DEFINITION OF PROTOCOL DEVIATION

A protocol deviation occurs when the participant, study coordinator or investigator fails to adhere to significant protocol requirements, including eligibility violations, deviation from intervention or other non-adherence to the protocol. Due to the nature of the trial, the treating clinicians are allowed to overrule the algorithm when they feel it is appropriate. Protocol deviations will be classified as a deviation, protocol violation or serious breach and the impact on participants' rights, safety, wellbeing, and data integrity will be classified as major, minor or no impact. We will also record whether the deviation requires follow-up, and the PI will determine if a violation results in withdrawal of a participant.

4.2.4 PRESENTATION OF PROTOCOL DEVIATIONS

The number and percentage of patients with major and minor protocol deviations will be summarised by treatment group with details of the type of deviation provided. Deviations that affect data integrity will be summarised in the final report.

4.3 ANALYSIS POPULATION

The primary analysis population will include all participants with a completed 'record of consent' form (completed by participants with capacity, a personal consultee if a participant lacks capacity, or a nominated consultee if a personal consultee cannot be identified, or if the participant died prior to obtaining consent) regardless of protocol deviations and adherence, and according to their randomised allocations (intention to treat), with complete data for both co-primary outcomes. A

participant is considered to have complete 28-day mortality data if they have either been confirmed to have died within 28 days or they are known to have been alive at day 28. A sensitivity analysis will be performed including all participants for whom consent has been obtained with complete data for at least one of the co-primary outcomes.

A secondary/sensitivity analysis will estimate the complier average causal effect (CACE) to account for departures from the randomised intervention. For the purposes of this sensitivity analysis, we will define different analysis populations depending on the level of adherence with the PCT-guided algorithm (Figure 3):

- Patients randomised to PCT-guided care in whom a PCT test is done, and a PCT result is available
- Patients randomised to PCT-guided care in whom a PCT test is done, a PCT result is available, and the clinician has seen the PCT result
- Patients randomised to PCT-guided care in whom a PCT test is done, a PCT result is available, the clinician has seen the PCT result and followed the algorithm exactly.

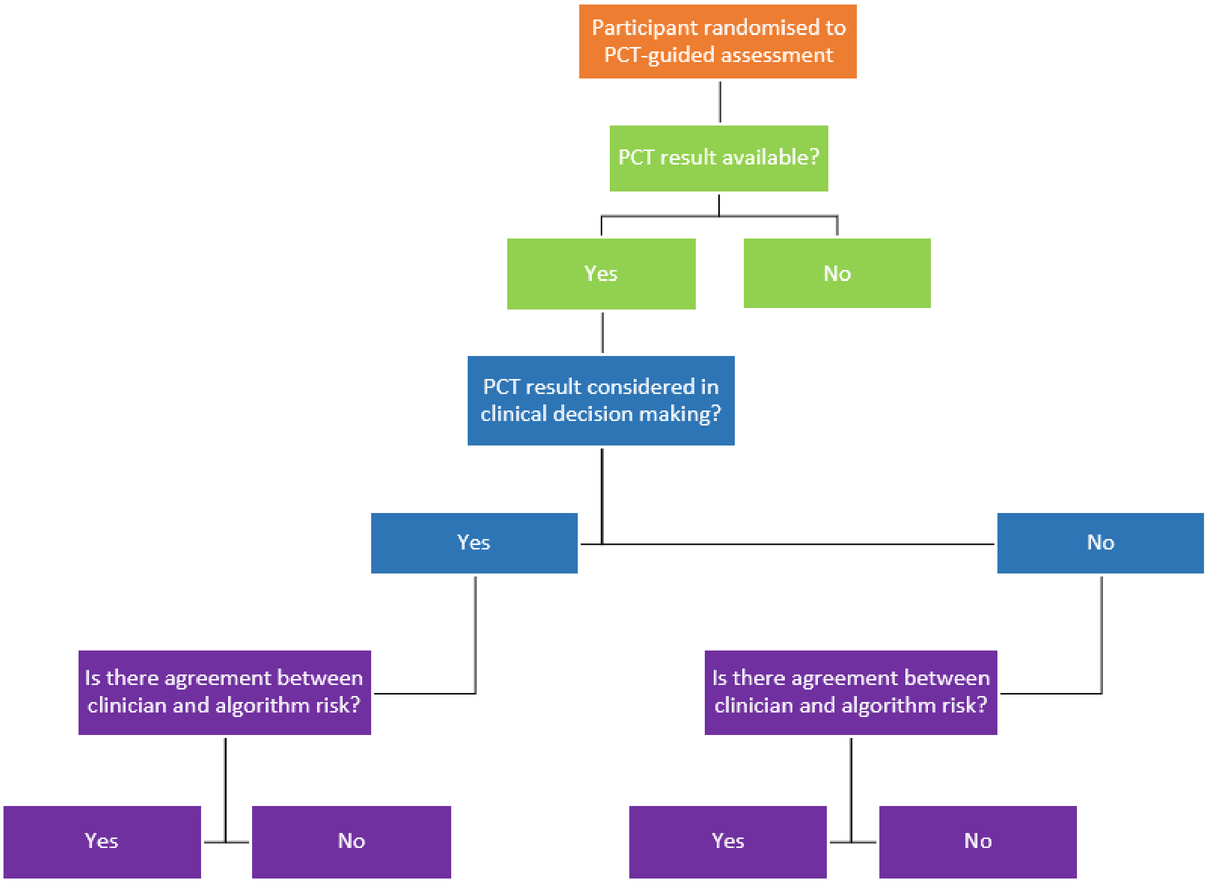

Figure 3: Flow diagram defining the different analysis populations in participants randomised to PCT-guided care

We will include a 4 by 4 table comparing the outcomes of the clinical risk assessment and the risk stratification/algorithm (Appendix Table A1).

5. STUDY POPULATION

5.1 SCREENING DATA

Patients with suspected sepsis will be identified at ED triage. After initial NEWS2 and assessment according to the current standard of care the eligibility criteria will be assessed and if no exclusion criteria apply, patients will be enrolled into the trial and randomised. A screening log of all eligible and randomised patients will be kept at each site so that any biases from differential recruitment will be detected. Tables will present the following summaries (overall and by study site): the number of days recruiting, number of patients screened, number of patients recruited, number of patients recruited per day, number of screened patients not recruited, and the reason for non-recruitment.

5.2 ELIGIBILITY

Participants are eligible for the trial if they meet all the following inclusion criteria and none of the exclusion criteria apply.

Inclusion criteria

- Patients ≥16 years presenting to the ED with suspected sepsis.

Exclusion criteria

- Currently on IV antibiotics.
- Current use of any chemotherapy agent associated with myeloablation/suppression.
- History of solid organ transplantation, allogeneic bone marrow, or stem cell transplantation within 3 months prior to consent.
- Patients requiring urgent surgical intervention.
- Presence of an advance directive to withhold life-sustaining treatment (patients not wishing to receive cardiopulmonary resuscitation (CPR) may qualify provided they receive all other resuscitative measures e.g., respiratory support, fluid resuscitation).

5.3 RECRUITMENT

As a deferred consent model is being used, patients and their relatives will be informed that a study is ongoing, but a lengthy consent discussion will not be had so as not to delay treatment. Should the patient or consultee wish not to take part at this point, then the decision should be respected, and the patient should not be enrolled into the trial. Patients who have given verbal consent will be

randomised regardless of baseline NEWS2. The process is described in the participant flow diagram (PRONTO Protocol Version 3.1 dated 23/10/23; Section 3; Figure 1).

5.4 TIERED CONSENT PROCESS

Due to time constraints in managing suspected sepsis, patients will be randomised into either the standard care arm or interventional arm on diagnosis with suspected sepsis prior to consent being obtained. The participant will be approached within 72 hours of randomisation to complete the formal informed consent process. The process is described in the participant consent flowchart (PRONTO Protocol Version 3.1 dated 23/10/23; Section 9.3; Figure 2). During this process participants can consent to different aspects of the trial using a tiered consent approach.

Participants can agree to the following aspects of the study:

1. Information collected as part of the trial and data from medical records from the date of randomisation up to the date of consent can be used in the trial.
2. Data from medical records can be collected from the date of randomisation to 90 days after this date.
3. Participant to be contacted by research staff at day 28 and day 90 to ask about health, wellbeing and any further medical treatment they may have received.

5.5 WITHDRAWAL/FOLLOW UP

If a patient does not wish to take part in any aspects of the trial, they will be withdrawn from the study and all clinical data up until that point will be removed from the study database. Participants have the right to withdraw consent for the use of clinical data collected in any aspect of the trial at any time. A participant's care will not be affected at any time by declining to participate or withdrawing from the trial.

5.5.1 LEVEL OF WITHDRAWAL

Some participants may wish to withdraw the use of the data upon first approach for deferred consent, following the intervention. If a participant provides deferred consent at this stage but subsequently withdraws from the trial, clear distinction must be made as to what aspect of the trial the participant is withdrawing from. These aspects could be:

1. Partial withdrawal from further data collection (questionnaires, clinical assessments)
2. Complete withdrawal from further data collection
3. Withdrawal of permission to use data already collected.

5.5.2 TIMING OF WITHDRAWAL

The numbers (with reasons) of losses to follow-up (dropouts and withdrawals) over the course of the trial (baseline, randomisation, treatment phase, day 28 and day 90 follow-up) will be presented in a CONSORT flow diagram.

5.5.3 REASONS FOR WITHDRAWAL

Participants who consent and subsequently withdraw are invited to complete a withdrawal form. If they decline, the withdrawal form should be completed by the researcher/clinician based on information provided by the participant. Participants will be identified as lost to follow-up if it is not possible to contact them directly for 4 weeks post day 90.

5.5.4 PRESENTATION OF WITHDRAWAL/LOSS TO FOLLOW-UP

We will report frequencies and percentages of participant withdrawal, overall and by trial arm, broken down by level, timing, and (where available) reason for withdrawal.

5.6 BASELINE PARTICPANT CHARACTERISTICS

5.6.1 LIST OF BASELINE DATA

- Age
- Gender
- Ethnicity
- Timing of initial assessments and ED admission
- Duration of symptoms
- Initial diagnosis
- Initial treatment
- History of oral antibiotic use in 14 days prior to admission
- Comorbidities (Charlson Comorbidity Index)
- COVID-19 status
- C-reactive protein (CRP) at baseline

5.6.2 DESCRIPTIVE STATISTICS

Participant characteristics will be summarised as frequencies and percentages, means and standard deviations, or medians and interquartile ranges, depending on the type of variable, for all randomised participants, overall and by trial arm, as well as for analysis populations as defined in Section 4.3, and by risk category based on NEWS2 score at baseline (≤4, 5-6 and ≥7).

6. ANALYSIS

6.1 OUTCOME DEFINITIONS

6.1.1 PRIMARY OUTCOME(S)

The study will use the following as co-primary outcomes:

- IV antimicrobial initiation at 3 hours (binary outcome)
- 28-day mortality (binary outcome)

Decisions about effectiveness using these co-primary outcomes will be made based on Table 3.

Table 3: determining whether the intervention is effective using the co-primary outcomes.

	Reduced antibiotic initiation	Same or more antibiotic initiation
Decreased mortality	Effective	Effective
Equivalent mortality	Effective	Not effective
Increased mortality	Not effective / harmful	Not effective / harmful

6.1.2 TIMING, UNITS AND DERIVATION OF PRIMARY

We will assess 28-day mortality from the date of randomisation, and IV antimicrobial initiation within 3 hours of ED admission. Both outcomes are recorded in binary format.

6.1.3 LIST OF SECONDARY OUTCOMES

1. Time until initiation of IV antibiotic therapy
2. Late IV antibiotic initiation – antibiotics commenced after 3 hours
3. Number of days on IV antibiotics (during admission and total over the first 28 days)
4. Number of days on any antibiotics (during admission and total over the first 28 days)
5. Number of days on broad spectrum antibiotics (IV and oral), defined by number of days on an 'Watch/Reserve' group antibiotic as defined by WHO AWaRe Classification Database (during admission and total over the first 28 days)
6. Critical care admission (ICU or HDU) – at any point during hospital admission
7. Length of ICU/HDU stay (overnight stays)
8. Length of hospital stay (overnight stays)
9. Adverse antibiotic outcomes (including *C. difficile* cases and hospital acquired infections (HAIs))
10. Readmission to hospital within 90 days (defined as readmission due to original diagnosis as per CRF)
11. Mortality within 90 days (and time until death)
12. Health utility (EQ-5D-5L) at 28 and 90 days
13. Health resource usage (described in the health economics analysis plan)
14. Feasibility of implementing PCT testing alongside NEWS2 scoring in EDs (described in the qualitative analysis plan)
15. Acceptability of implementing PCT testing alongside NEWS2 scoring in EDs, to patients, carers, and clinicians (described in the qualitative analysis plan)

6.1.4 ORDER OF TESTING

Not applicable.

6.1.5 TIMING, UNITS AND DERIVATION OF SECONDARIES

Individual timing and measurements of secondary outcomes are described in the table below:

Table 4: Timing and measurement of secondary outcomes

Outcome	Measure	Time frame
**Antibiotic initiation**	Time until initiation<br>Late IV antibiotic initiation (after 3 hours) (yes/no)	In ED/hospital
**Antibiotic usage**	No of days on IV antibiotics*<br>No of days on all antibiotics*<br>No of days on broad spectrum antibiotics (Watch/Reserve)*	In hospital and total over first 28 days
**Critical care usage (ICU and HDU)**	Admitted to ICU or HDU (yes/no)<br>No of overnight stays in ICU and HDU	In hospital
**Hospital stay**	No of overnight stays in hospital	In hospital
**Antibiotic adverse outcomes**	Anticipated drug reactions include diarrhoea, *C. difficile*, acute kidney injury, hearing loss, etc.	In hospital
**Readmission to hospital**	People with one or more readmissions	After discharge within 90 days
**Mortality**	Mortality within 90 days<br>No of days until death*	90 days
**Health utility**	EQ-5D-5L	28 and 90 days

*Days will be counted if an event occurs at any time between 00:00 and 23:59.

6.2 ANALYSIS METHODS

6.2.1 LIST OF METHODS AND PRESENTATION

6.2.1.1 BASELINE DEMOGRAPHICS ANALYSIS

Baseline data (e.g., age, gender, comorbidities, etc.) will be summarised by trial arm using appropriate descriptive statistics (section 5.5.2) and for those who completed follow-up compared to those lost to follow-up.

6.2.1.2 PRIMARY OUTCOME ANALYSIS

We will use complete case analysis for the co-primary outcomes, meaning that we will only include participants with valid responses to both co-primary outcomes in the primary analysis. We will fit separate two-level logistic regression models (patients nested within sites) to model both co-primary outcomes, controlling for baseline NEWS2 score (minimisation variable). Results will be reported as risk differences with corresponding confidence intervals (calculated via the delta method (Norton et al., 2013)); a two-sided 90% interval for 28-day mortality (OBF adjusted to 90.18%), and a two-sided

95% interval for IV antibiotic initiation at 3 hours (OBF adjusted to 95.04%). Non-inferiority in 28-day mortality will be concluded if the upper bound of the confidence interval is below +2.5% on the risk difference scale.

The individual analyses for the co-primary outcomes and corresponding inferences are described in the table below:

Table 5: Inferences for analyses of co-primary outcomes

**Test**	**Outcome**	**Hypothesis test and statistical method used***	**Inference**
**1**	28-day mortality	Non-inferiority test with a margin of 2.5%<br><br>H_0: $p_2 - p_1 \geq 0.025$	O'Brien-Fleming alpha spending (one-sided alpha = 0.05)<br><br>Reject null if P < 0.0491<br><br>Critical value = 1.654
**2**	28-day mortality	Superiority test<br><br>H_0: $p_1 - p_2 \leq 0$	O'Brien-Fleming alpha spending (one-sided alpha = 0.05)<br><br>Reject null if P < 0.0491<br><br>Critical value = 1.654
**3**	IV antibiotic initiation at 3 hours	Superiority test<br><br>H_0: $p_1 - p_2 \neq 0$	O'Brien-Fleming alpha spending (two-sided alpha = 0.05)<br><br>Reject null if P < 0.0496<br><br>Critical value = 1.964
*p_1 and p_2 represent the outcome proportions in the control and intervention arm, respectively.			

It has been recognised that the option to stop the trial early in sequential designs introduces bias to the standard maximum likelihood estimator (MLE) (Cox, 1952). Therefore, we will also calculate unconditional bias-adjusted point estimates. We will derive the bias-adjusted maximum likelihood estimates proposed by Whitehead, by subtracting an estimate of the bias from the MLE (Whitehead, 1986). We will also calculate the uniformly minimum variance unbiased estimator (UMVUE) using the Rao-Blackwell technique. We will calculate the unconditional bias-adjusted point estimates, as we want to determine the point estimates regardless of the stage at which the trial stops. We are interested in the bias as averaged over all possible stopping times, weighted by the respective stage-wise stopping probabilities (Robertson et al., 2023, Grayling & Wason, 2022).

Table 6: Summary of analyses of primary outcomes

**Outcome**	**Analysis**	**Covariates**
	Primary analysis:	Trial arm and baseline NEWS2

**IV antimicrobial initiation at 3 hours (superiority)**	Two-level logistic regression	Trial arm, baseline NEWS2, age, number of comorbidities and gender
	Secondary analysis: Two-level logistic regression with bias-adjusted point estimates	Trial arm and baseline NEWS2
		Trial arm, baseline NEWS2, age, number of comorbidities and gender
	Subgroup analyses: Two-level logistic regression (interaction test by model comparison)	Trial arm, baseline NEWS2, and the organ system of the infection (lower urinary tract, lower respiratory, intra-abdominal, bacteraemia, skin, soft tissues, etc.)
		Trial arm, baseline NEWS2, and risk category based on NEWS2 score at baseline (≤4, 5-6 and ≥7)
		Trial arm, baseline NEWS2, and managed as suspected COVID-19 during admission (yes/no)
		Trial arm, baseline NEWS2, and has a positive COVID-19 test result +/- 5 days from admission (yes/no)
		Trial arm, baseline NEWS2, and PCT machine used at the time of recruitment (BRAHMS PCT-direct/PathFast BRAHMS PCT)
		Trial arm, baseline NEWS2, and recruitment date (before 01/12/21, between 01/12/21 and 30/11/22, after 30/11/22)
		Trial arm, baseline NEWS2, and level of site ED crowding (upper, middle and lower tercile of national monthly figures)
	Complier average causal effect (CACE)	Trial arm, baseline NEWS2, and intervention adherence
**28-day mortality (non-inferiority)**	*Primary analysis:* Two-level logistic regression	Trial arm and baseline NEWS2
		Trial arm, baseline NEWS2, age, number of comorbidities and gender
	Secondary analysis: Two-level logistic regression with bias-adjusted point estimates	Trial arm and baseline NEWS2
		Trial arm, baseline NEWS2, age, number of comorbidities and gender
	Subgroup analyses:	Trial arm, baseline NEWS2, and the organ system of the infection (lower urinary tract, lower respiratory, intra-

	Two-level logistic regression (interaction test by model comparison)	abdominal, bacteraemia, skin, soft tissues, etc.)
		Trial arm, baseline NEWS2, and risk category based on NEWS2 score at baseline (≤4, 5-6 and ≥7)
		Trial arm, baseline NEWS2, and managed as suspected COVID-19 during admission (yes/no)
		Trial arm, baseline NEWS2, and has a positive COVID-19 test result +/- 5 days from admission (yes/no)
		Trial arm, baseline NEWS2, and PCT machine used at the time of recruitment (BRAHMS PCT-direct/PathFast BRAHMS PCT)
		Trial arm, baseline NEWS2, and recruitment date (before 01/12/21, between 01/12/21 and 30/11/22, after 30/11/22)
		Trial arm, baseline NEWS2, and level of site ED crowding (upper, middle and lower tercile of national monthly figures)
	Complier average causal effect (CACE)	Trial arm, baseline NEWS2, and intervention adherence

6.2.1.3 SECONDARY OUTCOME ANALYSIS

All secondary analyses will be performed on an intention to treat basis using the primary analysis population as defined in section 4.3, utilising two-level models to allow for patients nested within sites.

Table 7 – Summary of secondary analyses

	Outcome	Analysis	Covariates
1	Time until initiation of IV antibiotic therapy	We will present the result as a groupwise boxplot with median and range of number of hours. Cox regression analysis will be used, with the results reported as hazard ratios (HR) and 95% confidence intervals. We will also produce a Kaplan Meier plot and cumulative incidence plot.	a) Trial arm and baseline NEWS2<br>b) Trial arm, baseline NEWS2, age, number of comorbidities and gender

2	Late IV antibiotic initiation – antibiotics commenced after 3 hours	We will report the groupwise frequency and percentage of late initiations. Logistic regression will be used, with the results reported as odds ratios (OR) and 95% confidence intervals.	a) Trial arm and baseline NEWS2<br>b) Trial arm, baseline NEWS2, age, number of comorbidities and gender
3	Number of days on IV antibiotics (during admission)<br>Number of days on IV antibiotics (total over the first 28 days)	We will present the result as a groupwise boxplot with median and range of number of days. Poisson regression analysis will be used, with results reported as incidence rate ratios (IRR) and 95% confidence intervals.	a) Trial arm and baseline NEWS2<br>b) Trial arm, baseline NEWS2, age, number of comorbidities and gender
4	Number of days on any antibiotic (during admission)<br>Number of days on any antibiotic (total over the first 28 days)	We will present the results as a groupwise boxplot with median and range of number of days. Poisson regression analysis will be used, with results reported as IRRs and 95% confidence intervals.	a) Trial arm and baseline NEWS2<br>b) Trial arm, baseline NEWS2, age, number of comorbidities and gender
5	Number of days on broad-spectrum antibiotics (IV and oral) (during admission)<br>Number of days on broad-spectrum antibiotics (IV and oral) (total over the first 28 days)	We will represent the result as a groupwise boxplot with median and range of number of days. Poisson regression analysis will be used, with results reported as IRRs and 95% confidence intervals.	a) Trial arm and baseline NEWS2<br>b) Trial arm, baseline NEWS2, age, number of comorbidities and gender
6	Critical care admission (ICU or HDU) – at any point during admission	We will report the groupwise frequency and percentage of ICU and HDU admissions. Logistic regression will be used, with results reported as ORs and 95% confidence intervals.	a) Trial arm and baseline NEWS2<br>b) Trial arm, baseline NEWS2, age, number of comorbidities and gender
7	Length of critical care stay (overnight stays)	We will represent the result as a groupwise boxplot with median and range of number of days. Poisson regression analysis will be used, with results reported as IRRs and 95% confidence intervals.	a) Trial arm and baseline NEWS2<br>b) Trial arm, baseline NEWS2, age, number of comorbidities and gender

8	Length of hospital stay (overnight stays)	We will represent the result as a groupwise boxplot with median and range of number of days. Poisson regression analysis will be used, with results reported as IRRs and 95% confidence intervals.	a) Trial arm and baseline NEWS2<br>b) Trial arm, baseline NEWS2, age, number of comorbidities and gender
9	Adverse antibiotic outcomes	We will report the groupwise frequency and percentage of adverse outcomes events, overall and broken down by type of adverse event. Logistic regression will be used, with results reported as ORs and 95% confidence intervals.	a) Trial arm and baseline NEWS2<br>b) Trial arm, baseline NEWS2, age, number of comorbidities and gender
10	Readmission to the hospital within 90 days	We will report the groupwise frequency and percentage of readmissions. Logistic regression will be used, with results reported as ORs and 95% confidence intervals.	a) Trial arm and baseline NEWS2<br>b) Trial arm, baseline NEWS2, age, number of comorbidities and gender
11	Mortality within 90 days	We will report the groupwise frequency and percentage of deaths. Logistic regression will be used, with results reported as ORs and 95% confidence intervals.	a) Trial arm and baseline NEWS2<br>b) Trial arm, baseline NEWS2, age, number of comorbidities and gender
	Mortality within 90 days – days until death	Additionally, we will represent the result as groupwise a boxplot with median and range of number of days until death. Cox regression analysis will be used, with results reported as HR and 95% confidence intervals. We will also produce a Kaplan Meier plot and a cumulative incidence plot.	a) Trial arm and baseline NEWS2<br>b) Trial arm, baseline NEWS2, age, number of comorbidities and gender
12*	Health utility (EQ-5D-5L) at 28 days – summary index score (0-1)	We will report the mean (SD) of the score. A linear regression analysis will be used to test the difference between the groups.	a) Trial arm and baseline NEWS2<br>b) Trial arm, baseline NEWS2, age, number of comorbidities and gender
	Health utility (EQ-5D-5L) at 28 days – visual analogue scale (VAS) (0-100)		
	Health utility (EQ-5D-5L) at 90 days - summary index score (0-1)		

	Health utility (EQ-5D-5L) at 90 days – VAS (0-100)		
* For health utility (EQ-5D-5L) analyses, the current NICE guidelines suggest using the mapping function developed by van Hout et al. (2012). We will follow NICE guidelines at the time of analysis.			

6.2.2 COVARIATE ADJUSTMENT

Other covariates of potential interest, including age, comorbidities and gender, will be adjusted for in secondary analyses together with minimisation factors. Subgroup analyses are described in section 6.2.6.

6.2.3 ASSUMPTION CHECKING

Modelling and distributional assumptions will be checked prior to analysis and reporting. Specifically, time-to-event models will be tested for the proportional hazard assumption, Poisson regression models will be assessed for overdispersion and baseline NEWS2 score will be assessed for non-linearity.

6.2.4 ALTERNATIVE METHODS IF DISTRIBTIONAL ASSUMPTIONS NOT MET

If the data does not meet the distributional assumptions of the analyses described above, variables will be transformed if possible. If transformations do not improve the distributions, the model choice may vary from what is described above. For example, if the effect of baseline NEWS2 is distinctly non-linear we may account for it using higher-order terms or splines. If the number of days on antibiotics/in critical care do not meet the Poisson regression assumption that the variance is equal to the mean, we may use negative binomial or quasi-Poisson models to account for the overdispersion. A time interaction term may be added to Cox regression if the proportional hazards assumption is not met. Additionally, if the proportional hazards assumption is not met, we may consider restricted mean survival analysis, or possibly the Fine-Gray model if the assumption is badly violated. If censoring is an issue, rather than using boxplots, we will use the median and centiles from the survival distribution. Any changes will be fully documented.

6.2.5 SENSITIVITY ANALYSES

The primary analysis described in section 6.2.1 uses the joint complete case population for both co-primary outcomes. As a sensitivity analysis we will repeat the analysis using the complete case populations of each of the co-primary outcomes individually (i.e., including participants with valid responses to at least one of the co-primary outcomes) using the same methods as described in section 6.2.1.

A sensitivity analysis will estimate the CACE to account for departures from the randomised intervention. For the purposes of this sensitivity analysis, we will define different analysis populations depending on the level of adherence to the PCT-guided algorithm (Figure 3):

- Patients randomised to PCT-guided care in whom a PCT test is done, and a PCT result is available
- Patients randomised to PCT-guided care in whom a PCT test is done, a PCT result is available, and the clinician has seen the PCT result
- Patients randomised to PCT-guided care in whom a PCT test is done, a PCT result is available, the clinician has seen the PCT result and followed the algorithm exactly.

The non-inferiority margin is defined using a fixed risk difference of 2.5%. If the observed 28-day mortality in the control arm deviates from the 15% (by more than 3% points above or below) assumed in the sample size calculation, we will additionally repeat the primary analysis with the non-inferiority margin modified according to the power-stabilising arcsine transformation proposed by Quartagno et al. (2020 & 2023).

Also, the protocol allows patients to be recruited more than once into the study. If the proportion of repeatedly randomised patients exceeds 5%, we will perform a sensitivity analysis excluding repeat episodes.

6.2.6 SUBGROUP ANALYSES

We will conduct seven separate subgroup analyses, stratified by:

- The organ system of the infection (initial working diagnosis):
    - Upper respiratory tract infection
    - Skin/soft tissue infection
    - Urinary tract infection/urosepsis
    - Central nervous system infection
    - Gastrointestinal tract/abdominal infection
    - Bone/joint/muscle infection
    - Lower respiratory tract infection/community-acquired pneumonia
    - Sepsis (unknown source)
    - Other infections
    - Not infection
- Risk category based on NEWS2 score at baseline:
    - ≤4
    - 5-6
    - ≥7
- Managed as suspected COVID-19 during admission:
    - Yes
    - No
- Positive COVID-19 test result +/- 5 days from admission:
    - Yes
    - No
- PCT machine used at time of recruitment:
    - BRAHMS PCT-direct

- PathFast BRAHMS PCT
- Recruitment date:
  - Before 1st December 2021
  - Between 1st December 2021 and 30th November 2022
  - After 30th November 2022
- Level of site ED crowding (percentage of patients who spent >12 hours from decision to admit to admission) at time of randomisation:
  - Upper tercile of national monthly figures
  - Middle tercile of national monthly figures
  - Bottom tercile of national monthly figures

We will investigate whether the treatment effect varies between subgroups by including the subgroup variable as a covariate in the main analysis model, both with and without a treatment-arm interaction term. The models with and without the interaction term will be compared using a likelihood-ratio test (LRT). We will report the LRT χ^2 statistic and illustrate the direction of the subgroup effect using forest or interaction plots.

We will also assess whether adherence to the intervention varies according to levels of ED crowding at the time of randomisation. We will conduct a two-level logistic regression (patients nested within sites) with algorithm adherence (yes/no) as the outcome variable and the level of ED crowding as the explanatory variable, controlling for baseline NEWS2 score (minimisation variable). NHS England publishes monthly A&E attendances and emergency admissions data. We will calculate the percentage of emergency admission patients who spent >12 hours from decision to admit to admission per month per site and compare these to the national monthly figures. We will rerun the co-primary analyses for subgroups of the least, middle, and most crowded EDs. As an exploratory analysis we may include ED crowding as an additional covariate and include time varying interaction terms. If the data allows, we may also explore the effect of daily ED crowding levels on the co-primary outcomes for a subgroup of sites.

6.3 MISSING DATA

During data entry, validations have been written into the system to minimise the amount of missing data, however, missing data may still occur. The frequency and percentage of missing values will be reported overall and by arm. If missing primary outcome data is greater than 5% (as accounted for in the sample size calculation), we will conduct sensitivity analyses using multiple imputations (separately for both randomisation groups) (Sullivan et al., 2018). Missing data will be investigated for cause and extent and multiple imputation. The assumption of missingness at random (MAR) will be tested by analysing each baseline covariate in a separate logistic regression model to determine which (if any) are associated with the missingness of the primary outcome, and the associated p-values will be reported alongside the summary statistics. Missing observations will be replaced by multiple imputations by chained equations (MICE). A sensitivity analysis will be conducted on the primary analysis, including any baseline factors that were found to be associated with the

missingness of the primary outcome. Any changes to the assumptions made in the primary analysis will be considered in a sensitivity analysis.

6.4 ADDITIONAL ANALYSES

We will compare the initial working diagnosis versus the final diagnosis by arm.

We may perform a linear regression analysis on an intention to treat basis using the primary analysis population as defined in section 4.3, utilising two-level models to allow for patients nested within sites, to explore the differences in Daily Defined Doses of antibiotics by arm.

The health economics and qualitative analysis plans are detailed separately from the SAP.

6.5 HARMS

The trial population comprises very sick adults, and hospitalisation is normal in this population. Events such as prolongation of existing hospitalisation, life threatening events and death are also expected in this population and are recorded as part of outcome data collection and therefore are not subject to expedited reporting on an SAE form.

For the purposes of this trial the following events will not require reporting as SAEs:

- Death
- Life threatening event
- Hospitalisation or prolongation of hospitalisation
- Admission to ICU
- Non-serious AEs potentially attributable to PCT test and step-down approach will be collected as part of routine follow-up at 28 days.
- Other non-serious AEs will not be collected.

These events will be recorded in the participant's notes and on the relevant CRFs.

The following will be reported as SAEs within 24 hours:

- Events resulting in persistent or significant disability or incapacity
- Congenital anomalies or birth defects

The frequency (percentage) of AEs and SAEs will be tabulated overall and by trial arm and compared using a chi-square test.

6.6 STATISTICAL SOFTWARE

The data will be extracted and imported into Stata (version 17.0 or higher). All analyses will be carried out in Stata (StataCorp LLC, College Station, TX, USA).

7. REFERENCES

7.1 NON-STANDARD STATISTICAL METHODS

Altman DG, Bland JM (2005) Treatment allocation by minimisation. *BMJ*, **330**, 843.

Cox, DR (1952). A note on the sequential estimation of means. *Mathematical Proceedings of the Cambridge Philosophical Society*, **48**, 447-450.

DeMets DL, Lan KK (1994) Interim analysis: the alpha spending function approach. *Statistics in Medicine*, **13**, 1341-1352.

Dimairo M, Pallmann P, Wason J, et al. (2020) The adaptive designs CONSORT extension (ACE) statement: a checklist with explanation and elaboration guideline for reporting randomised trials that use an adaptive design. *Trials*, **21**, 528.

Euden J, Thomas-Jones E, Aston S, et al. (2022) PROcalcitonin and NEWS2 evaluation for Timely identification of sepsis and Optimal use of antibiotics in the emergency department (PRONTO): protocol for a multicentre, open-label, randomised controlled trial. *BMJ Open*, **12**, e063424.

Grayling MJ, Wason JM (2023) Point estimation following a two-stage group sequential trial. *Statistical Methods in Medical Research*, **32**, 287-304.

Jennison C, Turnbull BW (1989) Interim analyses: the repeated confidence interval approach. *Journal of the Royal Statistical Society: Series B (Methodological)*, **51**, 305-334.

Morris TP, Walker AS, Williamson EJ, White IR (2022) Planning a method for covariate adjustment in individually randomized trials: a practical guide. *Trials*, **23**, 328.

Norton EC, Miller MM, Kleinman LC (2013) Computing adjusted risk ratios and risk differences in Stata. *Stata Journal*, **13**, 492-509.

O'Brien PC, Fleming TR (1979) A multiple testing procedure for clinical trials. *Biometrics*, **35**, 549-556.

Offen W, Chuang-Stein C, Dmitrienko A, et al. (2007) Multiple co-primary endpoints: medical and statistical solutions: a report from the Multiple Endpoints Expert Team of the Pharmaceutical Research and Manufacturers of America. *Drug Information Journal*, **41**, 31-46.

Piaggio G, Elbourne DR, Pocock SJ, Evans SJ, Altman DG, CONSORT Group (2012) Reporting of noninferiority and equivalence randomized trials: extension of the CONSORT 2010 statement. *JAMA*, **308**, 2594-2604.

Quartagno M, Chan M, Turkova A, Ford D, White IR (2023) The Smooth Away From Expected (SAFE) non-inferiority frontier: theory and implementation with an application to the D3 trial. *Trials*, **24**, 556.

Quartagno M, Walker AS, Babiker AG, et al. (2020) Handling an uncertain control group event risk in non-inferiority trials: non-inferiority frontiers and the power-stabilising transformation. *Trials*, **21**, 145.

Robertson DS, Choodari-Oskooei B, Dimairo M, Flight L, Pallmann P, Jaki T (2023) Point estimation for adaptive trial designs I: A methodological review. *Statistics in Medicine*, **42**, 122-145.

Robertson DS, Choodari-Oskooei B, Dimairo M, Flight L, Pallmann P, Jaki T (2023) Point estimation for adaptive trial designs II: practical considerations and guidance. *Statistics in Medicine*, **42**, 2496-2520.

Sullivan TR, White IR, Salter AB, Ryan P, Lee KJ (2018) Should multiple imputation be the method of choice for handling missing data in randomized trials? *Statistical Methods in Medical Research*, **27**, 2610-2626.

Van Hout B, Janssen MF, Feng YS, et al. (2012) Interim scoring for the EQ-5D-5L: mapping the EQ-5D-5L to EQ-5D-3L value sets. *Value in Health*, **15**, 708-715.

Whitehead J (1986) On the bias of maximum likelihood estimation following a sequential test. *Biometrika*, **73**, 573-581.

7.2 DATA MANAGEMENT PLAN

Data management plan: P:\152310305\PRONTO\eTMF\8.0 Data Management\8.1 Data Management \DOCUMENTS\DMP

7.3 TRIAL MASTER FILE AND STATISTICAL MASTER FILE

Trial master file: P:\152310305\PRONTO\eTMF

Statistical master file: P:\152310305\PRONTO\eTMF\8.0 Data Management\8.5 Statistics

7.4 OTHER SOPS OR GUIDANCE DOCUMENTS

Randomisation plan: P:\152310305\PRONTO\eTMF\8.0 Data Management\8.5 Statistics\ Randomisation

Interim SAP: P:\152310305\PRONTO\eTMF\8.0 Data Management\8.5 Statistics\Statistical Analysis Plan\Interim SAP

SAP DEVIATION LOG

**Document number:**		**Document version:**	
**Reason for deviation:**			

Deviations from the planned analyses:

- For the ED subgroup analysis, we have used the site's % of participants waiting greater than >12hours from decision to admit at the time of randomisation, rather than the tercile, to reflect the increasing ED crowding
- We have not calculated the MLE or UMVUE estimates described in section 6.2.1.2 as the IDMC decided to continue the trial after the interim analysis even though the trial could have stopped according to the stopping criteria described in section 3.5.2

The following post-hoc exploratory analyses have been conducted on the following dates to understand the mechanism behind the results:

24/06/24:

- Descriptive tabulation of antibiotics and broad-spectrum antibiotics by arm
- Descriptive tabulation of ventilation and vasopressor by arm
- Descriptive tabulation of the participant timeline/pathway by arm
- Descriptive tabulation of the co-primary outcomes (28-day mortality and IV abx within 3-hours) by arm and NEWS2 score
- Descriptive tabulation of the algorithm risk vs clinical risk assessment by arm and NEWS2

11/07/24:

- Breakdown of amoxicillin and co-amoxiclav by IV/oral in the descriptive table of antibiotics by arm
- Descriptive tabulation of non-antibiotics (e.g., antivirals, anti-parasitic etc.) prescribed by arm
- Descriptive tabulation of the 10 most frequent combinations of first 2 abx prescribed simultaneously
- Descriptive summary (median [IQR]) of the number of days on broad-spectrum abx per participant per arm
- Descriptive tabulation of the proportion of participants who received two narrow spectrum abx simultaneously as their first abx
- Descriptive tabulation of 28-day mortality by algorithm risk category by arm
- Descriptive summary of the mean days on any abx by algorithm risk category by arm
- Explore whether mortality (adjusted for NEWS2) differed by site using post estimation of random effects

15/08/24:

- Descriptive tabulation of the first prescription given by arm
- Post-hoc subgroup analysis (using same methods described in section 6.2.6) of time to IV (secondary outcome #1) by NEWS2
- Poisson regression analysis of days on IV/any abx in first 14 days
- Logistic regression analysis of whether IV abx was initiated within 12 hours

- Logistic regression analysis of whether IV abx was initiated within 7-days
- Descriptive summaries of the following, to explore whether the treatment pathway may have changed between the arms
    - Time from triage (+/- randomization) to prescription of antibiotics
    - Time from triage (+/- randomization) to administration of antibiotics
    - Time between prescription and administration of antibiotics
    - % blood cultures taken in first 24 hours (No other measure in CRF)
    - Broad spectrum Abx (Watch/Reserve) vs narrow spectrum (Access)
    - Initial Diagnosis Infection vs Not Infection
    - Cause of Death
    - Time to HDU/ITU admission
    - Broad spectrum Abx (Watch/Reserve) vs narrow spectrum (Access)
    - Abx choice – Compare distribution of No Antibiotics> PO>IV Narrow>IV broad
    - % Microbiology tests in first 24 hours (Blood cultures only)
    - % Microbiology tests in first 24 hours (Any)
    - % Any antimicrobial resistance detected, if yes which by %
    - Duration of antibiotics (initial course started within 24 hours of attendance)
    - Length of hospital stay
    - % No antibiotics vs any antibiotics
    - % Late course of antibiotics (2-28 days)
    - Time to late antibiotics
    - % Readmitted to hospital
- Descriptive summary of the days on no abx, oral, iv broad, iv narrow, combination for each NEWS score and arm

05/09/24

- Tabulate first prescription within 24 hours from triage by arm
- Tabulate arm by final diagnosis with mortality in the cells – infection vs non infection
- Tabulate deaths in withdrawals by arm

19/09/24

- Tabulate 28-day mortality in different algorithm adherence groups – including the discordant groups (low clinical risk & high algorithm risk and vice versa) (unexpected vs expected PCT result)
- A logistic regression analysis of trial arm's effect on being alive and not readmitted at 90 days
- Subgroup analysis (as described in section 6.2.6) of grading of clinician conducting assessment
- Subgroup analysis (as described in section 6.2.6) of index of deprivation decile
- Tabulate time between 1st and 2nd assessment overall, by arm and by NEWS2
- Time series plot of ED crowding by site
- Subgroup analysis (as described in section 6.2.6) of initial diagnosis of LRTI/CAP vs all other initial diagnoses

- Subgroup analysis (as described in section 6.2.6) of initial diagnosis of respiratory infection vs all other initial diagnoses

02/12/24

- Descriptive comparison of initial diagnosis and final diagnosis by arm using Sankey diagrams

09/12/24

- Descriptive tabulation of 28-day morality by arm by type of abx received within 12 hours

8. APPENDICES

8.1 Dummy tables

8.1.1 Primary outcomes

Table A1 – Adherence to PCT-guided algorithm

	**Clinical risk assessment**				
**Algorithm**	Risk	Low	Low/medium	Medium	High
	Low	N =			
	Low/medium				
	Medium				
	High				

Table A2 – Analysis populations defined by adherence to intervention.

	**Level**	**n/N (%)**
**PCT test result available**	Yes	
	No	
	Missing	
**PCT test result considered in clinical decision making**	Yes	

	No	
	Missing	
**Algorithm adhered to**	Yes	
	No	
	Missing	

Table A2 – Primary outcome analysis results

**Outcome**	**Analysis**	**Treatment effect estimate [95% CI]**	**p-value**
**IV antimicrobial initiation at 3 hours (superiority)(a)**	Primary	RD =	
	Adjusted for covariates	RD =	
	Bias-adjusted	RD =	
	CACE	RD =	
**28-day mortality (non-inferiority) (a)**	Primary	RD =	
	Adjusted for covariates	RD =	
	Bias-adjusted	RD =	
	CACE	RD =	

CI = confidence interval, RD = risk difference.
Analysis method: (a) multilevel logistic regression.
Confidence intervals are two-sided for IV initiation and one-sided for 28-day mortality.
Covariates in all models: baseline NEWS2.

8.1.2 Secondary outcomes

Table A3 – Secondary outcome analysis results

	**Outcome**	**Analysis**	**Treatment effect estimate [95% CI]**	**p-value**
**1**	Time until initiation of IV antibiotic therapy(a)	Primary	HR =	
		Adjusted for covariates	HR =	
**2**	Late IV antibiotic initiation – antibiotics commenced after 3 hours(b)	Primary	OR =	
		Adjusted for covariates	OR =	

**3**	Number of days on IV antibiotics (during admission) [(a)]	Primary	IRR =	
		Adjusted for covariates	IRR =	
	Number of days on IV antibiotics (total over first 28 days) [(a)]	Primary	IRR =	
		Adjusted for covariates	IRR =	
**4**	Number of days on any antibiotic (during admission) [(a)]	Primary	IRR =	
		Adjusted for covariates	IRR =	
	Number of days on any antibiotic (total over first 28 days) [(a)]	Primary	IRR =	
		Adjusted for covariates	IRR =	
**5**	Number of days on broad-spectrum antibiotics (IV and oral) (during admission) [(a)]	Primary	IRR =	
		Adjusted for covariates	IRR =	
	Number of days on broad-spectrum antibiotics (IV and oral) (total over the first 28 days) [(a)]	Primary	IRR =	
		Adjusted for covariates	IRR =	
**6**	ICU or HDU admission – at any point during admission[(b)]	Primary	OR =	
		Adjusted for covariates	OR =	
**7**	Length of ICU/HDU stay[(a)]	Primary	IRR =	
		Adjusted for covariates	IRR =	
**8**	Length of hospital stay[(a)]	Primary	IRR =	
		Adjusted for covariates	IRR =	
**9**	Adverse antibiotic outcomes[(b)]	Primary	OR =	
		Adjusted for covariates	OR =	
**10**	Readmission to the hospital within 90 days[(b)]	Primary	OR =	
		Adjusted for covariates	OR =	
**11**	Mortality within 90 days[(b)]	Primary	OR =	
		Adjusted for covariates	OR =	
	Mortality within 90 days - time until death[(a)]	Primary	HR =	
		Adjusted for covariates	HR =	
**12**	Health utility (EQ-5D-5L) at 28 days[(c)]	Primary	MD =	
		Adjusted for covariates	MD =	
		Primary	MD =	

	Health utility (EQ-5D-5L) at 28 days[c]	Adjusted for covariates	MD =	
	Health utility (EQ-5D-5L) at 90 days[c]	Primary	MD =	
		Adjusted for covariates	MD =	
	Health utility (EQ-5D-5L) at 90 days[c]	Primary	MD =	
		Adjusted for covariates	MD =	

CI = confidence interval, HR = hazard ratio, OR = odds ratio, MD = mean difference.
Analysis method: (a) cox regression, (b) logistic regression, (c) linear regression
Covariates in all models: baseline NEWS2.

8.1.3 Subgroup analyses

Table A4 – Subgroup analyses of primary outcomes

Outcome	Subgroups	LRT χ^2 (df)	p-value
**IV antimicrobial initiation at 3 hours (superiority)[a]**	The organ system of the infection		
	Risk category based on NEWS2 score at baseline		
	Managed as suspected COVID-19 during admission		
	Positive COVID-19 test result +/- 5 days from admission		
	PCT machine used		
	Recruitment date		
	Level of ED crowding		
**28-day mortality (non-inferiority) [a]**	The organ system of the infection		
	Risk category based on NEWS2 score at baseline		
	Managed as suspected COVID-19 during admission		
	Positive COVID-19 test result +/- 5 days from admission		
	PCT machine used		
	Recruitment date		
	Level of ED crowding		

Analysis method: (a) multilevel logistic regression. Interaction tests by model comparison.
Covariates in all models: baseline NEWS2.
